# Decoupling Photochemical and Photothermal Effects Using Molecular Motors Enables Fast, Intelligent, and Life‐Like Motions in Soft Materials

**DOI:** 10.1002/adma.74045

**Published:** 2026-07-16

**Authors:** Guiying Long, Jinyu Sheng, Alexander Ryabchun, Ben L. Feringa

**Affiliations:** ^1^ Stratingh Institute for Chemistry University of Groningen Groningen The Netherlands; ^2^ SCNU‐UG International Joint Laboratory of Molecular Science and Displays National Center for International Re‐Search on Green Optoelectronics South China Normal University Guangzhou China; ^3^ College of Chemistry Chemical Engineering and Materials Science Soochow University Suzhou Jiangsu China

**Keywords:** liquid crystal polymer network, molecular motor, photochemical effect, photothermal effect, soft robotics

## Abstract

The development of synthetic smart materials that perform on‐demand tasks in complex environments, while triggered noninvasively, is a major goal to develop the next generation of soft robotics. However, fast and complex locomotion with high spatial‐temporal precision using external stimuli controlling shape in such systems remains a major challenge. Here, we describe a light‐responsive soft material based on liquid crystal polymer networks (LCPNs) with variable stiffness comprising highly efficient light‐driven molecular rotary motors and dye molecules. This system selectively responds to light of specific wavelengths, enabling highly programmable and complex motions, such as jumping, rotating, and climbing. The rapid response of molecular motor triggered by UV‐light (photochemical effect) and the heat generated by dye upon red‐light irradiation (photothermal effect), can be decoupled and orthogonally controlled, due to unique features of the molecular motor. Our study shows how cooperativity and amplification of molecular motion can lead to the rapid actuation of synthetic materials, which offers novel molecular tools and materials engineering perspectives for the development of intelligent soft robotics.

## Graphical abstract

A light‐responsive liquid crystal (LC) polymer network integrating molecular rotary motors and photothermal converters enables the decoupling of photochemical and photothermal effects. Selective wavelength activation allows orthogonal control over persistent shape encoding and fast reversible motion, enabling programmable soft robots capable of complex locomotion behaviors.

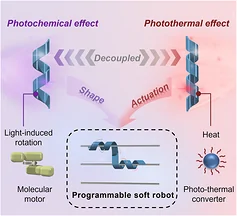

## 1 Introduction

Biology has long served as a source of inspiration for developing increasingly sophisticated artificial intelligent materials. Mechanical functions, motility, and adaptive behavior are important and fundamental natural traits that stimulate the emergence of actuation materials. Smart soft matter systems, capable of large shape deformations and autonomous motion in response to external stimuli, have shown significant potential in various fields, including artificial muscles, sensors, wearable devices, and healthcare, being a major incentive for the design of future intelligent materials [1, 2, 3, 4, 5, 6, 7, 8]. Artificial soft actuators are a class of functional materials capable of precise control of motion, which have recently attracted considerable attention by the scientific community [9, 10]. Light‐actuated systems are particularly appealing for their remote (contactless) nature and tunability in terms of intensity, wavelength, and exposure time with high spatial‐temporal precision [11, 12, 13, 14]. The key to the targeted control of such materials lies in the ability to coordinate and amplify light‐triggered molecular motions across multiple length scales, particularly from the nano‐dimensions to the macroscopic level, effectively converting light energy into mechanical work [1, 2, 3, 15]. Liquid crystal (LC) materials, with their highly ordered molecular arrangement, exhibiting collective molecular motion and rapid responses under external stimulation, are arguably among the most ideal responsive soft materials to develop actuators, in addition to biomimetic hydrogel materials [4, 16, 17, 18, 19, 20, 21, 22].

Light‐responsive liquid crystal polymer networks (LCPNs, also referred to as LCNs in the literature) are of particular interest as these mesogenic‐based crosslinked polymeric soft materials which in recent years have exhibited remarkable actuation and shape‐change behavior upon illumination [17, 23, 24, 25]. The key design for reversible light‐driven deformation relies on the incorporation of photoswitch units including azobenzene derivatives [26, 27, 28, 29, 30, 31], diarylethenes [32, 33, 34], hydrazones [35], or molecular motors [36, 37, 38, 39, 40, 41], all of which undergo specific structural transformations upon exposure to light of specific wavelengths. However, the conversion of light energy into mechanical work due to operation of switches and motors (photochemical effect) is often compromised or even dominated by photothermal effects, which not only leads to the waste of light energy but more importantly might interfere with the precise control over the materials responsive function. Although our recent work [41] demonstrated that the first‐generation molecular motors can drive macroscopic deformations exclusively due to photochemical transformation, the majority of current light‐responsive LC polymer systems are unable to distinguish and separate photochemical and photothermal effects [14, 26, 27, 28, 37, 38, 39, 40], which not only limits precise control over actuation but also hampers potential applications as well as a deeper understanding of light‐to‐mechanical energy conversion mechanisms. Therefore, decoupling the photothermal effect from the photochemically driven macroscopic phenomena is crucial to understand the role of light and demonstrating smart actuation systems with enhanced photo‐efficiency achieving high spatial and temporal precision. Smart hydrogel‐based actuator systems can mitigate thermal effects, although shape encoding might be compromised by their inherent isotropic nature [3, 4, 16, 42, 43]. The development of smart LCPN systems that combine fast programmable and intelligent behavior with high precision motility using molecular motors for light‐driven soft matter and devices provides major opportunities.

**FIGURE 1:**
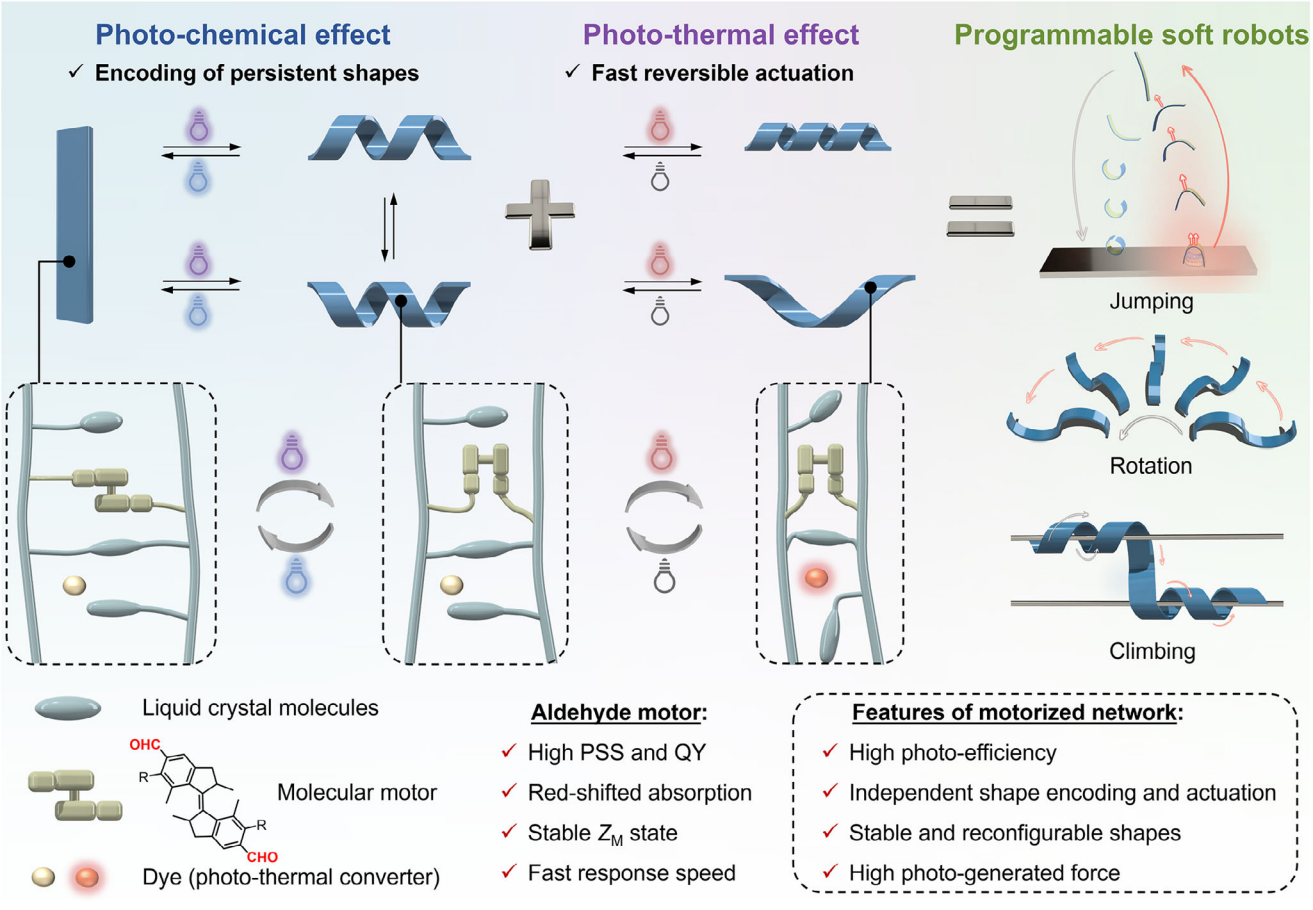
Liquid crystal polymer network energized by molecular rotary motors with boosted performance demonstrating their potential applications in programmable soft robotics. Illustration of the principle of decoupling of photochemical and photothermal effects in motorized LCPNs. The rotation of the molecular motor builds up internal strains and therefore accounts for photochemical shape encoding. The photothermal effect is due to the incorporated dye molecules and is responsible for fast reversible actuation. A variety of persistent shapes including helical ones can be interconverted as a result of the molecular motor operation while not being affected by photothermal actuation. The interplay of these effects enables complex mechanical movement relevant for future programmable soft robotics such as jumping, rotation, and climbing.

In the present study, the design and fabrication of highly‐efficient light‐responsive smart soft matter systems are reported based on tunable LCPNs in which we could decouple and orthogonally regulate photochemical and photothermal effects to achieve life‐like locomotion behavior (Figure 1). The heart of this system consists of our recently discovered highly photo‐efficient aldehyde functionalized molecular motor (MDA) [44] and a robust photothermal convertor, DB‐14 dye (1,4‐bis(methylamino)anthracene‐9,10‐dione). Specifically, MDA exhibits excellent photoconversion (quantitative conversion in the photostationary state, PSS), high photoisomerization quantum yield (QY up to 80%), and large geometrical difference between various distinct states, enabling fast and precise encoding of macroscopic shapes of LCPNs under low‐energy UV light exposure. Independently, DB‐14 can efficiently harvest energy from red light and convert it into heat, reducing the molecular order within the LCPNs, thereby facilitating rapid and reversible actuation. In addition, we studied three LCPNs with variable stiffness to understand the interplay between photochemical and photothermal effects and to reveal the key parameter for shape‐shifting and light‐generated force. This study provides fundamental insights into the respective contributions of photochemical and photothermal effects and as well the actuation mechanism of motorized polymer materials. The concept of purposeful decoupling of both mechanisms is further applied for programmable shape encoding and actuation behavior with remarkable performance under light stimulation including jumping, rotation, and climbing. This unique design strategy not only offers fundamental guidance toward smart soft materials enabling complex motions but also represents a significant conceptual step beyond existing approaches implementing conventional photoswitches, paving the way for the next generation of high‐performance light‐driven smart materials and future programmable soft robotics.

## 2 Results and Discussion

### 2.1 Material Design

**FIGURE 2:**
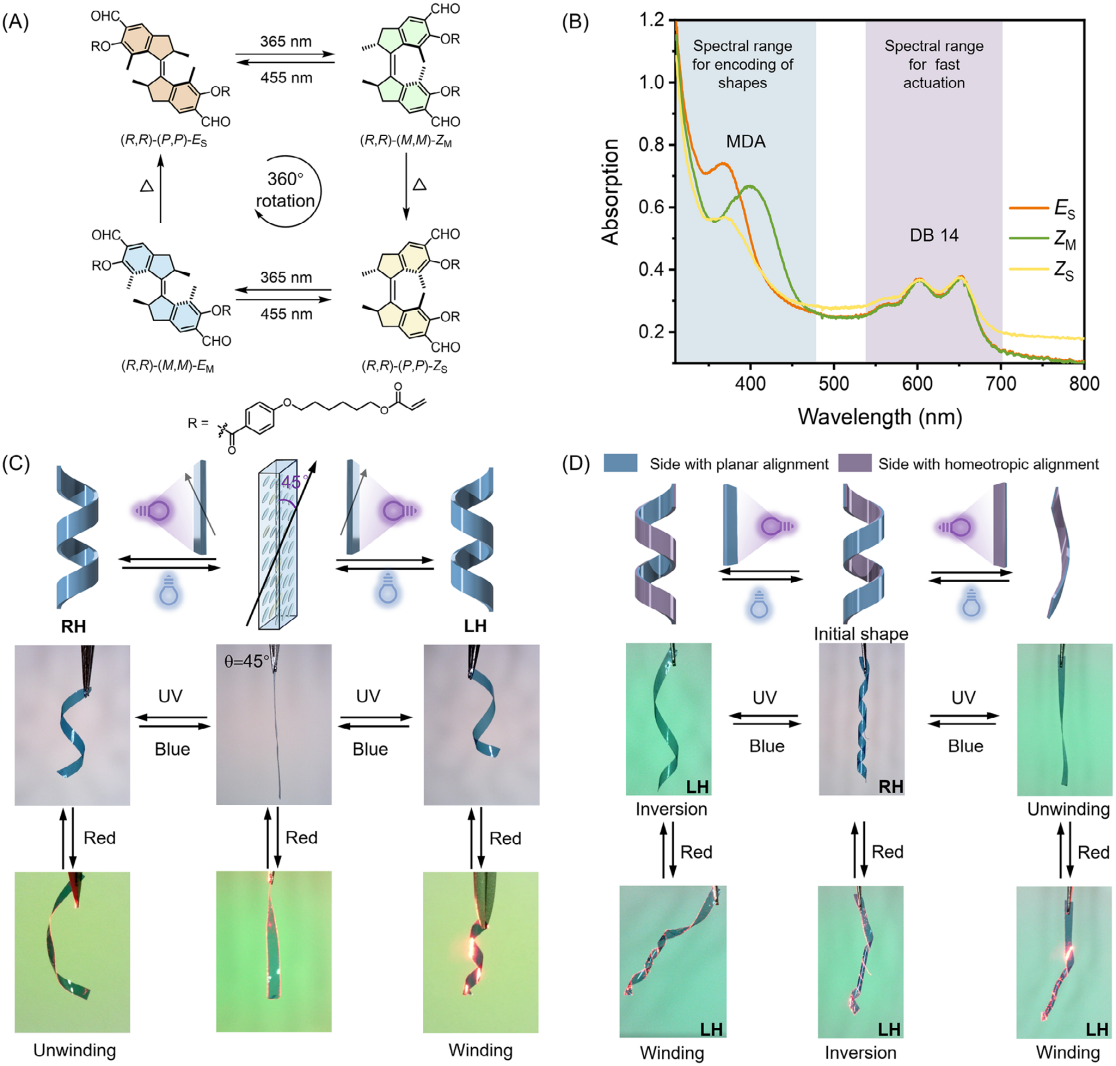
(A) Full rotational cycle of molecular motor MDA, which consists of two photochemical steps of *E*/*Z* isomerization followed thermal relaxation (thermal helix inversion, THI). Only one enantiomer is shown for simplicity. (B) Absorbance spectra of the **LCPN 2** film containing MDA and DB‐14. The film with motor in *E*_S_ state was irradiated with 365 nm UV light for 10 min to reach *Z*_M_ state followed by annealing at 120°C for 2 h to accelerate formation of *Z*_S_ state. (C) Demonstration of decoupling of photochemical and photothermal effects in an **LCPN 2** ribbon with a unidirectional planar LC structure (*θ =* 45°). Both, left‐handed (LH) and right‐handed (RH) helical shapes can be programmed by choosing which side of the ribbon is exposed to UV light. The symmetry breaking is the result of the motor generating mechanical strain only from one side causing folding and twisting from an initially flat shape into a helical one. Helical shapes with opposite handedness demonstrate opposite actuation behavior upon irradiation with red light. This behavior results from the modulus gradient established during polymerization, where the side of the material facing the polymerization light source has a higher crosslinking density. In LH helices, the inner side has a lower modulus, making the molecular order more susceptible to photothermal disruption, leading to contraction, whereas RH helices show opposite behavior. (D) Demonstration of decoupling of photochemical and photothermal effects in an **LCPN 1** ribbon with homeo‐planar LC structure (*θ =* 135°). Initially the RH helical shape of the ribbon originates from interplay between splay alignment and cutting angle. However, the strain generated by molecular motors rotation upon UV irradiation from the side with planar molecular alignment can outcompete asymmetry caused by splay alignment resulting in inverting handedness of the helical ribbon. UV exposure from the side of the ribbon with vertical alignment leads to almost complete unfolding of the initially helical shape. In all cases, the photothermal effect upon red light stimulation winds the ribbons into tight LH helices. It originates from the temperature increase due to the photothermal effect and associated reduction of the LC order within the network. This reduction leads to contraction and expansion of the film at planar and homeotropic sides, respectively, which destabilizes the original helical structure. Notably, UV‐stimulated shapes were retained and reversible under blue light, whereas red‐light‐induced deformations recovered immediately after ceasing light exposure. The intensities of UV, blue and red light were 1.2, 423, and 480 mW/cm^2^, respectively. UV exposure time was 3 and 15 min for blue light.

In this study, we applied first‐generation molecular motors [41, 44, 45, 46, 47] featuring aldehyde groups, causing a red shift in absorption and a remarkably enhanced performance. The bis‐aldehyde motor showed excellent photo‐efficiency with nearly complete conversion in each step of a rotary cycle and high stability (long half‐life) of the *Z*_M_ state (Figure 2A). Acrylate terminal groups were incorporated to facilitate covalent crosslinking of motor into the LCPN. Important to highlight here a significant practical advantage: the red‐shifted operational window of this motor design. While prior analogue [41] required transparent and stable LC building blocks specifically synthesized for the deep UV region, the current system supports a wide variety of commercially available LC materials. The chemical structures of LCPN components, the synthetic routes, and characterization of motors MDA and control compound (MMA) are provided in Table S1 and Figures S1–S10. A C6 aliphatic spacer between the acrylate units and motor core provided flexibility and sufficient free space for rotation within the polymer matrix. A phenyl ring, linked via an ester bond, improved compatibility of the motor with the LC components while enhancing the change in molecular length during the distinct isomerization steps comprising the rotary cycle (Figure 2A). This design gives the rotary motor outstanding photochemical performance and shape changes and combined with high structural stability, making it particularly suited for integration into LCPNs.

These light‐driven molecular motors (MDA and MMA) were synthesized and then copolymerized as covalent crosslinkers with LC monomers to form LCPNs, enabling the efficient transfer of molecular motions to the macroscopic scale. The content of the MDA was set at 3 wt% as in the previous reports [35, 41, 48]. Furthermore, the dye DB‐14 [29], a photothermal converter that absorbs red light and converts it into heat, was introduced into the LC material. To control the elastic modulus of the LCPNs, a non‐polymerizable low molar mass LC mixture (E7), was used. In addition, a monoacrylate‐based aldehyde motor (MMA) was designed as a control compound, which unlike the MDA carries an acrylate group only at one end (Figure S1 and Table S1).

### 2.2 Rotatory Behavior of Molecular Motor in Solution

The molecular motor MDA undergoes a four‐step unidirectional rotation under external stimuli, completing a full 360° cycle. The optimized geometries of four motor states are shown in Table S4. This cycle includes two photochemical isomerization steps, each followed by a thermal helical inversion (THI) step (Figure 2A), as confirmed by ^1^H‐NMR and UV–vis spectroscopy.

The isomer ratio upon photochemical conversion was quantified by ^1^H‐NMR, yielding a *Z*_M_/*E*_S_ ratio of 97:3 and a *Z*_S_/*Z*_M_ ratio exceeding 99:1 (Figure S11). The remarkable, highly efficient isomerization process results in a PSS ratio exceeding that of any previously reported motors [3]. UV–vis spectroscopy studies showed that the absorbance maximum of the motor shifted to 363 nm as compared to 300 nm for the unsubstituted motor [41], which allowed us to use commercially available LC components for the fabrication of LCPNs, which drastically increased the potency of such motors for various applications. In addition, *E*_S_‐MDA undergoes photoisomerization to form the *Z*_M_‐MDA upon UV irradiation (365 nm), and this process is reversible under blue light irradiation (455 nm) (Figure S12B,C). The THI process (*Z*_M_→*Z*_S_) was also monitored in real time using UV–vis spectroscopy, indicating a half‐life of ∼438 h for *Z*_M,_ (Figure S13), testimony that *Z*_M_‐MDA is relatively stable. Similar studies were conducted using the control compound MMA and the properties of MMA in solution closely resemble those of MDA (Figures S12 and S13). Additionally, the quantum yield (QY) of the photochemical isomerization of MDA (*E*_S_→*Z*_M_) was determined to be ∼71% (Figure S14), demonstrating that molecular motor MDA exhibits significantly higher efficiency compared to first‐generation unsubstituted molecular motors [41].

### 2.3 Rotatory Behavior of Molecular Motor in LCPNs

We anticipate that the efficiency of molecular motion transduction by the motor is influenced by the rigidity of its environment [38, 39, 41, 49, 50, 51]. To investigate this, LCPNs were used with increasing elastic modulus values achieved by varying the amount of E7. The compositions of the corresponding monomeric mixtures and mechanical properties of the resulted networks are compiled in Tables S1–S3, respectively. The detailed mechanical performance of these systems will be discussed in the next section. Based on their elastic modulus, we distinguish three types of networks: soft (**LCPN 1**), medium (**LCPN 2**), and stiff (**LCPN 3**). This approach enabled us to systematically compare the driving effects of MDA on the LCPNs. For details of sample preparation and characterization, see Figures S15 and S16.

To confirm that MDA maintained its rotary motion when integrated into LCPN as a cross‐linker, UV–vis measurements were conducted using **LCPN 2**. Figure 2B (orange line) shows two initial absorption bands at 368 nm (*E*_S_‐MDA) and 600–660 nm (DB‐14). Upon UV irradiation, the 368 nm band decreases while a new 400 nm band appears, indicating MDA photoisomerization (green line). Meanwhile, the absorption band of DB‐14 remains unchanged, confirming that UV light does not affect the dye. Subsequently, the 400 nm band blue‐shifts to 370 nm due to the THI process (yellow line). A similar study was performed on the control sample containing MMA (Figures S17B and S18C–E). The results indicate that the unidirectional rotational behavior of MDA is preserved within the LCPN material. Notably, the half‐life data for MDA in solution (438 h), **LCPN 2** (1452 h), and MMA in the control sample (627 h), suggests that covalent incorporation into the LCPN reduces the rotational frequency of motor. Furthermore, the separation of absorption bands between MDA and DB‐14 (Figure 2B) enables selective operation: UV light (365 nm) and blue light (455 nm) can drive the molecular motor, while red light (660 nm) can activate DB‐14 to generate heat.

To understand the orientation of molecular motors relative to the surrounding LC fragments in the network, we performed TD‐DFT calculations and polarized UV–vis spectroscopy measurements. TD‐DFT calculations indicate that MDA undergoes significant molecular contraction upon photoisomerization (22.2 Å→8.9 Å), while maintaining nearly the same dimensions before and after THI (8.9 Å→9.1 Å) (Figure S19A). Figure S19B reveals that *E*_S_‐MDA is primarily aligned along the LC molecules when embedded in the network. Upon photoisomerization, *Z*_M_‐MDA predominantly adopts an orientation perpendicular to the LC alignment direction.

### 2.4 Shape Encoding and Actuation of LCPNs

As described above the molecular motors are co‐aligned with LC fragments and capable of rotation in the network. Therefore, the conventional surface‐induced LC alignment techniques can synchronize (direct) motors rotation in space which enables precise programming the specific shape of the LCPNs. We anticipate that the motor isomerization induces molecular geometrical changes (*E*_S_→*Z*_M_), which pull the network along the alignment direction, resulting in the buildup of mechanical stresses. This stress can be released during network deformation [38, 39, 40, 52] making its precise control crucial for shape encoding.

To demonstrate that MDA embedded in LCPN can induce macroscopic deformation under light stimulation and to investigate its actuation effect, we exploited **LCPN 2** unless otherwise stated. Under low‐intensity UV irradiation (1.2 mW/cm^2^), MDA isomerization caused the ribbon to bend toward the light, while blue light restored this process, with no noticeable fatigue over multiple cycles (Figure S23). Additionally, given the long half‐life of the *Z*_M_ state (1452 h) and its geometric similarity to the *Z*_S_ state, effectively “locking” the network shape, results in the unique feature of shape stability (Figure S24). This behavior contrasts sharply with azobenzene‐based systems, which typically relax back to their original shape over time (accelerated by heat) [14, 29, 53, 54]. It is worth noting a fundamental distinction between the current first‐generation molecular motor and previously reported second—generation systems [38, 39]. These motors undergo deformation under UV light intensities over 80 times lower than their counterparts. Notably, the resulting shape is retained after the light is removed, whereas second generation motor‐based networks revert to their original state. This suggests that in the latter case, photothermal and photochemical effects contribute simultaneously, preventing the encoding of persistent shapes or the decoupling of these distinct mechanisms.

As previously shown, motor isomerization determines the network shape. Other critical factors influencing shape are the mechanical properties of the network, particularly the anisotropy of the elastic modulus, which arises from LC structures (e.g., planar, splay, and twist). Here, we address how molecular alignment and motor‐induced strain gradients influence the network shape. For parallel‐aligned LCPNs, ribbons with a cutting angle (*θ*) of 0° exhibit bending deformation toward the UV light source due to uniaxial pulling forces asserted on the exposed surface of the ribbon. When the cutting direction forms an angle with the alignment direction, local contraction becomes non‐uniform, leading to a global twisting (Figures S22A and S22B). Furthermore, we investigated the photothermal effect of **LCPN 2** and its interplay with the photochemical response. Under red‐light irradiation, a strong photothermal effect rapidly increases the system's temperature (Figure S31D), reducing the molecular order. As shown in Figure 2C, a ribbon with *θ* = 45° exhibits slight twisting under red‐light, while a LH helix formed under UV light winds rapidly upon red‐light exposure (Video S1). In contrast, a ribbon cut at *θ* = −45° and folded into a RH helix under UV light unwinds under red‐light (Video S2). The deformations are fully reversible upon stopping red‐light irradiation. Prolonged red‐light activation does not cause any further shape change or further heat accumulation due to relatively low thickness of the ribbons (25 µm) and high surface area‐to‐volume ratio. Notably, the shape of the ribbons can be reversible encode under UV‐blue light irradiation, while red light‐induced actuation driven by the photothermal effect does not alter the preprogrammed polymer shape, confirming the effective decoupling of these phenomena. Besides our current design integrating decoupling within a single material, it can also be achieved by spatially or chemically separating photoactive components to enable orthogonal light processing. Li et al. demonstrated this different approach by incorporating a push‐pull azobenzene and an arylazopyrazole into separate LC network materials assembled within one device [55]. Their distinct spectral properties allowed the azobenzene to drive photothermal actuation, while the arylazopyrazole triggered photochemical actuation.

To verify that shape change of the ribbon occurs due to MDA rotation, we prepared a control network similar to **LCPN 2** but containing monoacrylate MMA lacking cross‐linking abilities and therefore cannot pull the network. The control material exhibited no shape change under UV light but maintained reversible actuation under red light (Figure S25). This confirms that actuation in MDA‐based LCPNs under UV irradiation originates from pulling of the network by the motor rotation and associated molecular shape changes, while deformation under red light results from a temperature‐induced reduction in LC order.

As noted earlier, the LC configuration is another important determinant of polymer ribbon shape. To examine how it can be used with motorized networks to encode shape and actuation behavior, we used a soft **LCPN 1** film with splay alignment (fabrication details in Section s7). The anisotropic stress distribution from splay alignment caused the ribbon to fold into a specific shape due to nonuniform deformation, highlighting the intricate relationship between LC configuration and mechanical behavior [17]. Accordingly, when **LCPN 1** films were cut at different angles, the internal stress gradient across the thickness led to asymmetric stress release, forming various shapes (Figures S22C and S22D). Investigated the photothermal and photochemical effects of the system, we demonstrate here for the first time how light‐generated stress can overcome the inherent internal strain in the homeo‐planar structure, thereby reversing the macroscopic chirality (the ribbons exhibit RH→LH helical chiral inversion when UV irradiation is applied to the planar‐aligned side, and flat deformations when UV irradiation is applied on homeo‐aligned side, Figure 2D). However, the actuation mode under red light illumination is still fundamentally governed by the LC configuration, so that the films exhibit tightening toward the LH helix (Videos S3–S5). This highlights the fact that photochemical and photothermal effects play their distinctive roles in the LCPN system.

**FIGURE 3:**
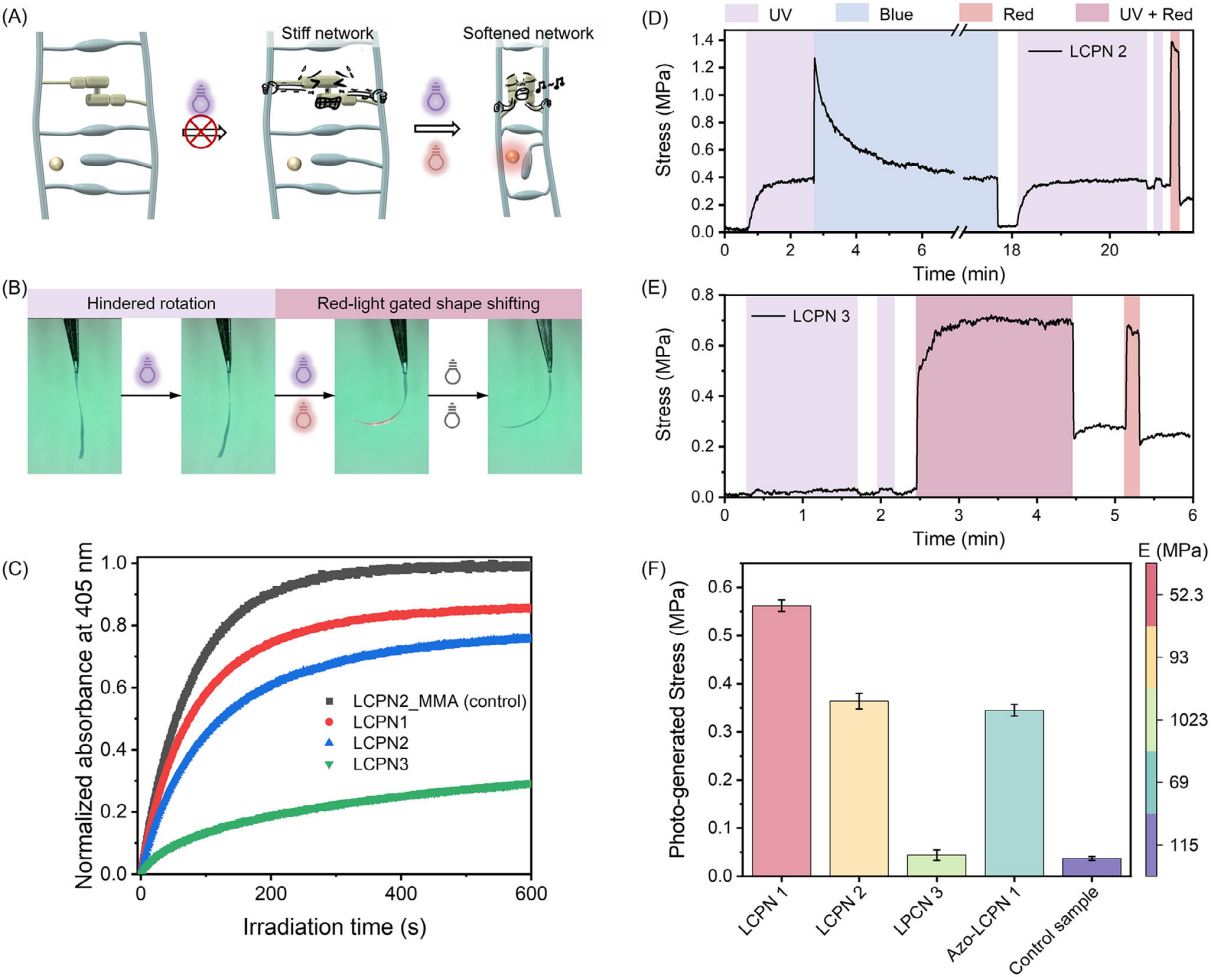
(A) Schematic illustration showing that in **LCPN 3**, the rotation of molecular motors under UV irradiation is hindered by the rigid polymer network. Upon simultaneous red‐light irradiation, **LCPN 3** undergoes thermal softening, allowing motor rotation under UV light, accompanied by corresponding shape changes in the film (B) After removing all stimuli, the deformed shape is retained, indicating that the shape is due to the photochemical effect of motor rotation. (C) UV–vis spectral traces of the photochemical process in different LCPN systems under UV irradiation. The normalized kinetic curves compare the relative proportion and rate of photoconversion (*E*_S_→*Z*_M_) for molecular motors embedded in different networks. Mechanical stress generated in **LCPN 2** (D) and **LCPN 3** (E) ribbons upon light stimulation with different wavelengths. UV and blue light irradiation induce a gradual accumulation and subsequent release of stress in the materials, attributed to the photochemical rotation of molecular motors. Conversely, the rapid increase of stress upon red light irradiation is linked to a photothermal effect, wherein light absorbed by the dye DB‐14 dissipates as heat. (F) Maximal UV light‐generated mechanical stress across all tested networks with the color code corresponding to the elastic modulus of each network (as indicated on the right). Error bars represent the standard error, derived from at least three measurements. Stress/force measurements were conducted under isometric conditions at constant strain (∼0.5%) applied to the ribbons. The intensities of UV, blue, and red light were 18.5, 423.0, 479.4 mW/cm^2^, respectively.

So far, we have extensively discussed the relatively soft networks incorporating E7 as a plasticizer. However, for some practical applications, motors/switches need to operate within rigid polymer networks, making it essential to explore their performance in stiffer matrices. To this end, we fabricated and studied **LCPN 3** (composition in Table S1, fabrication details in Section S7). Since its glass transition temperature (*T*_g_) is above r.t. (34°C, Figure S15F), it is hypothesized that UV stimulation induces mechanical stress in the molecular motors, but the excessive network rigidity prevents macroscopic deformation [29]. As shown in Figure 3B, ribbons cut along the alignment direction exhibited negligible deformation after UV exposure. In order to release the accumulated internal light‐induced stress, we heated (70°C) the network to soften it (please note that all LCPNs here do not exhibit isotropization, Figure S15). To our surprise, we didn't observe any significant shape change. However, when the ribbon was exposed to UV light at this elevated temperature, significant curling was observed (Figure S27). This behavioral difference suggests that the rigid network might partially hinder motor rotation.

To further investigate the impact of network stiffness on motor photoisomerization, we prepared thin films of the networks between two quartz substrates and monitored the motor isomerization (absorption band at 405 nm, corresponding to *Z*_M_‐MDA formation) using UV–vis spectroscopy. We used our control network containing motor attached only from one side (MMA) to normalize the absorbance value assuming that in this case rotation of the motor is not hindered. Kinetic curves reveal that the conversion in the control sample was the fastest (Figure 3C, black line). However, when the motor is incorporated as a crosslinker or when the network rigidity increases, the conversion rates significantly decreased. For example, only about one‐fourth of the motors rotated in **LCPN 3** (Figure 3C, green line) after 10 min of irradiation. The origin of such hindered rotation is not yet clear but might originate from a large elastic modulus (Table S2 and Figure S30) and lack of free volume in the networks having high glass transition temperature, as it was recently highlighted for azobenzene‐containing LCPNs [56]. Importantly, the heating **LCPN 3** softened the network, effectively “unlocking” the motor rotation evidenced by the increase of the photoconversion (Figure S28) which is consistent with shape programming experiments. Thus, tunable rigidity of the networks represents here a mechanical gate for shape shifting. These results are at contrast with those previously reported for azobenzene‐containing LCPNs of similar rigidity, where photothermal effect “exposed” accumulated strains originated from photochemical transformation of azobenzene, thereby creating a synergetic contribution of both mechanisms [29]. It is essential to highlight that network rigidity imposes constraints on motor rotation, a factor that must be given consideration during the design process of motorized polymer materials. This phenomenon can also be a deliberate design paradigm which would allow circumventing accidental shape‐shifting of soft actuators or unintended isomerization during storage or under ambient UV exposure.

### 2.5 Mechanical Forces Originated From Motor Rotation

To gain a deeper understanding of the processes underlying light‐induced motion of polymer films, extensive mechanical measurements were conducted. Stress‐strain curves for all studied systems were recorded using a tensile tester. Analysis of these curves allowed us to determine the elastic moduli of **LCPN 1–3** as 52, 93, and 1023 MPa, respectively (Table S2). These results indicate that the content of E7 determines significant differences in mechanical stiffness, with higher E7 concentrations leading to lower elastic moduli. We anticipate that mechanical properties of the networks play an important role in the operation of molecular motors.

Upon UV irradiation, molecular motors are expected to convert light energy into mechanical work, inducing macroscopic shape changes. To investigate this phenomenon, we conducted systematic studies under varying material compositions and irradiation conditions, comparing the results with LCPNs containing conventional azobenzene photoswitches. Azo derivative used was a diacrylate‐terminated crosslinker and was incorporated at an identical concentration of 3 wt% to be topologically and chemically identical to the MDA LCPN system. To measure the photo‐induced force and stress, LCPN ribbons were fixed at both ends and subjected to a pre‐strain (∼0.5%), while a load cell continuously recorded the mechanical force generated upon irradiation [48]. Based on preliminary tests at various UV light intensities (Figures S31A and S32A), 18.5 mW/cm^2^ was defined as the optimal UV intensity. The intensity is sufficiently powerful to activate as many motors as possible, while simultaneously generating a low thermal contribution (typically below 10%), which enables the moment of switching on/off the light to be clearly discerned directly on the stress curves. The values of photochemically generated stress/force were obtained by calculating the difference between total recorder stress and its photothermal contributions. Notably, the UV intensity 1.2 mW/cm^2^ used for shape encoding experiments was significantly lower than the threshold for the photothermal effects, confirming that shape encoding was driven solely by a photochemical effect. An additional control experiment was conducted in cold (5°C) water, which demonstrated the efficiency of shape‐shifting in response to UV light (Figure S26).

Figure 3D illustrates the typical stress response of the **LCPN 2** film. UV irradiation induced a gradual stress increase, reaching equilibrium after 2 min. Upon switching to blue light, stress increased rapidly due to photothermal effects (temperature rises to 34.4°C, as shown in Figure S32B), then gradually decreased to an equilibrium value over 15 min. After turning off the blue light, the stress returned to its initial value, indicating that the recovery of molecular motor (*Z*_M_→*E*_S_), fully released the UV‐induced stress. When exposed to red light, the film exhibited an almost instantaneous (approx. 1 s, Figure S34) stress increase due to rapid temperature rise as a result of photothermal effects, followed by complete stress recovery after turning off the irradiation. Similar experiments on **LCPN 1** produced consistent results (Figure S31B). Further analysis of **LCPN 2** demonstrated that higher red‐light intensity generated greater stress (Figure S31C), and the stress‐temperature relationship followed a linear trend with light intensity (Figure S31D), confirming that red‐light‐induced mechanical stress was primarily thermally driven. In the case of **LCPN 3** (Figure 3E), minimal mechanical stress variation under UV light was observed due to higher stiffness of the network and hindered motor rotation as we discussed above. In other words, the photochemical effect (motor rotation) is “locked” by the mechanical state of the network. However, the switching on of red light led to a significant stress increase, as the photothermal effect (temperature increase) softened the polymer film, thereby “unlocking” constrained motor rotation. That establishes AND‐gate where two stimuli (red and UV light) are necessary to yield macroscopic shape change (output). This effect was further corroborated by the observation that residual stress remained significantly higher than the initial value after irradiation ceased. Additionally, control experiments revealed that **MMA** could not generate mechanical stress, thus failing to induce deformation under UV irradiation (Figure S31E).

The significant influence of network stiffness, used as a control factor for mechanical action and motion, on motor rotation was further validated by comparing the mechanical stress generated by MDA motors embedded in **LCPN 1–3**. As shown in Figure S33, increasing E7 content, which softens the network, resulted in a marked increase in stress, reaching a maximum of 0.55 MPa (∼27 mN). These findings confirm that incorporating E7 reduces motor rotation constraints, thereby enhancing mechanical response, consistent with UV–vis spectroscopy studies.

Furthermore, the photoinduced stress generated by MDA motors was compared with that of conventional azobenzene photo‐switches. As depicted in Figure S33, the UV‐induced stress in **LCPN 1** (red line) was nearly twice that of **Azo‐LCPN 1** (blue line). This difference arises because azobenzene undergoes *trans*–*cis* isomerization under UV irradiation, during which its molecular length decreases from approximately 9.0 to 5.5 Å [57]. In contrast, MDA motors exhibit a much more pronounced conformational change, with their molecular length varying from about 22.2 to 8.9 Å upon UV exposure (Figure S33). This substantially larger geometric change (nearly four times that of azobenzene) induces greater perturbation within the polymer network, thereby leading to more significant macroscopic deformation and higher stress output in MDA‐based LCPN systems. The bar graph in Figure 3F further presents specific stress values alongside network elastic moduli, with detailed photochemical stress contributions for all samples provided in Table S3. Notably, the stress induced by the photochemical reaction in **LCPN 1** (0.52 MPa) and **LCPN 2** (0.32 MPa) was 17 and 11 times higher, respectively, than that of LCPN containing conventional first‐generation motors (0.03 MPa) [41]. These results indicate that MDA‐based systems generate significantly higher stress to drive deformation compared to conventional photoswitches or previously reported motors under similar experimental conditions. Therefore, this novel dialdehyde motor with unique photochemical performance is confirmed as a highly effective responsive unit, making it particularly suitable to achieve high levels of mechanical control in soft actuators.

### 2.6 Programmable Soft Robots

**FIGURE 4:**
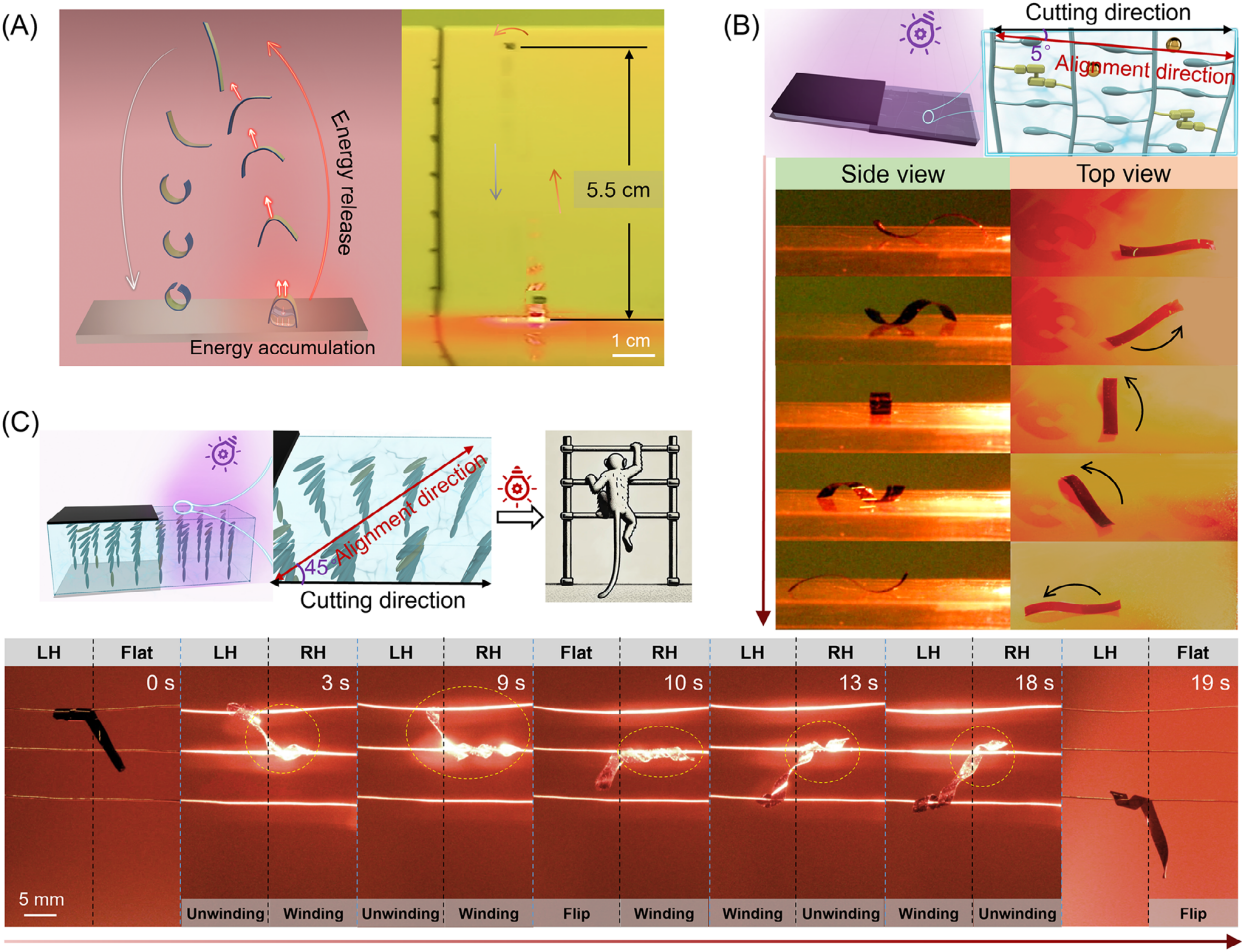
Application of MDA‐based LCPN actuators in soft robotics. (A) A splay‐aligned LCPN film with a cutting angle of 0° forms a ring shape with a diameter of 1.57 mm (film size: 5 × 2 mm) and exhibits a jumping motion under red light irradiation. The left panel illustrates the energy accumulation and release mechanism driving the jump, while the right panel presents the trajectory of the jumping film. (B) A planar‐aligned LCPN film (15 × 2 mm) with a cut angle of 5° undergoes rotational motion under the synergistic effect of photochemical and photothermal. The upper section depicts the shape encoding by irradiation LCPN film with UV light through the mask. The lower section presents both the side and top views of rotational trajectory of the actuator under red light. (C) A splay‐aligned LCPN film (25 × 2 mm) with a cut angle of 45° exhibits climbing motion under the synergistic effect of photochemical and photothermal stimulation. The upper panel illustrates the LC structure and shape encoding upon UV irradiation, where molecular motor‐induced modulus variation, coupled with molecular alignment, and enables complex deformations. Under red light, the robot undergoes a climbing down motion resembling a monkey's climbing behavior. The lower section presents the trajectory of the film under red light actuation. The yellow dashed circle indicates the red‐light‐irradiated area. The intensity of the UV light and red light were 1.2 and 479.4 mW/cm^2^, respectively.

Building on the systematic investigation of the motorized LCPN systems, we demonstrate here its potential to achieve complex motions with potential for future applications in soft robotics. We selected the **LCPN 1** system for further experimental validation. First, a splay‐aligned LCPN film (0° cutting angle) was prepared, initially curving due to an internal stress gradient. Compressing its ends formed a closed ring. Under red light, the actuator underwent a two‐step response: first, the ring‐shaped actuator tended to undergo a flipping deformation due to photothermal effects, leading to energy accumulation. Once reaching a critical energy threshold, the compressed ends suddenly open, initiating the second phase energy release. This instantaneous energy discharge propelled the actuator to jump 5.5 cm (35 BL, body length) within 80 ms, demonstrating the rapid actuation capability of the system under photothermal stimulation (Figure 4A and Video S6). Next, a parallel‐aligned LCPN film with a cutting angle of 5° was fabricated in order to show the synergistic contribution of photochemical and photothermal effects. The film was first subjected to shape programming via UV exposure using a masking technique. The film was divided into two regions, with the upper surface of one section and the lower surface of the other section selectively exposed to UV light (Figure 4B, top panel, and Videos S7, S8). The molecular motor‐induced actuation resulted in a wavy deformation. Subsequently, when one end of the film was subjected to red light, a slight angular mismatch between the long axis and the alignment direction of the film induced a twisted deformation. As a result, under continuous red‐light irradiation, the film exhibited rotational motion about the non‐irradiated end. Finally, to achieve more complex biomimetic locomotion, highlighting the system's potential for soft robotics, we prepared a splay‐aligned LCPN film with a 45° cutting angle. Similar to the previous experiment, a masking technique was used to divide the film into two sections. As shown in Figure 4C (top panel), UV irradiation on the right side counteracted intrinsic alignment stress, flattening it while the left side retained its LH helical state. The actuator, wrapped around a wire, was exposed to localized red‐light irradiation, triggering RH helical winding in the right portion and securing it onto a second wire. The subsequent exposure to red‐light irradiation of the left side caused the spring to unwind while the spring on the right side tightened, ultimately leading to a flipping motion. When irradiation stopped on the left side, it returned to its LH helical state and wrapped around a third wire. Gradually reducing red light intensity on the right section induced progressive unwinding, culminating in another flipping event (Figure 4C, bottom panel). Controlled by light, this actuator successfully climbed down from the first to the third wire (Video S9), mimicking the coiling of climbing plants or primate grasping movements. This demonstration highlights the amazing potential of high‐efficiency molecular motors MDA in LCPN systems, showcasing their ability to decouple photochemical and photothermal effects and achieve programmable, adaptive, and biomimetic motion for soft robotic applications.

## 3 Conclusions

In this study a soft LCPN system is presented based on a novel high‐performance molecular machine, that is a rotary motor, capable of decoupling photochemical and photothermal effects for precise control of fast shape programming and actuation. The aldehyde‐functionalized molecular motor undergoes a conformational change under UV stimulation, which is amplified to macroscopic deformation, enabling shape encoding for the system. Meanwhile, the incorporation of a dye molecule facilitates red light‐driven disruption of the molecular order within the LCPN. Tuning stiffness of LCPNs (**LCPN 1–3**) allows diverse and on demand motion patterns. Mechanical tests confirm that the designed system exhibits remarkably enhanced mechanical properties with respect to previous reported systems. Overall, this work shows developments of an LCPN system that integrates efficient light energy conversion, independent shape encoding, programmable actuation, high stability and reversibility, reconfigurability, and exceptional mechanical performance. This artificial system emphasizes the importance of decoupling photothermal and chemical effects in amplifying molecular motion and demonstrates that fast complex motions, including jumping, rotation, and climbing can be achieved using molecular motors. These advancements will inspire the future development of advanced adaptive soft robotics and pave new avenues for the next generation of smart materials.

## 4 Methods

### 4.1 Synthesis

The synthetic routes of motors MDA and MMA, as well as the structural characterization of all intermediates involved (^1^H‐NMR, ^1^ ^3^C‐NMR, and HRMS), are provided in Sections S2 and S3.

### 4.2 Spectroscopic Measurements

NMR spectra of the rotational cycles of the motor in solution were collected on a Varian Unity Plus spectrometer (^1^H: 500 MHz, ^1^ ^3^C: 126 MHz). UV–vis absorption spectra of samples in solution were measured on a Hewlett–Packard 8454 diode array spectrometer in a 1 cm quartz cuvette. In situ irradiation experiments were performed using fiber‐coupled Thorlabs LEDs (M365FP1, M455F3). UV–vis spectra of LCPN films were recorded using an AVASPEC‐ULS2048CL‐EVO (Avantes) spectrometer. Polarized UV–vis spectra were collected using the same instrument equipped with a Glan–Taylor polarizer mounted on a rotation stage (ELL14K, Thorlabs), allowing adjustment of the polarization angle in 10° increments. Differential scanning calorimetry (DSC, TA instrument DSC Q1000) measurements were performed with two thermal cycles and the second heating curves were analyzed. For sample **LCPN 1**, the DSC measurements before and after polymerization were carried out over a temperature range from −50°C to 120°C; for **LCPN 2** and **LCPN 3**, the corresponding range was −20°C to 150°C. The heating and cooling rates were both set to 5°C/min, and the samples were held for 5 min at the maximum and minimum temperatures. Optical imaging and phase behavior studies were performed using polarized optical microscope (POM) Eclipse LV100N‐POL (Nikon, Japan), equipped with a heating stage (HS82, Mettler–Toledo). A red color filter was employed to prevent photopolymerization of the LC mixture under visible light. The phase transition behavior of the LC mixtures was monitored during heating and cooling between 25°C and 100°C at a rate of 5°C/min.

### 4.3 Samples Preparation

LC layers were prepared by using home‐made 25µm‐thick glass cell with polyimide aligning layers. Details are provided in Section S6. Motorized LCPNs were produced by blue light (*λ* = 470 nm) photopolymerization of monomeric mixtures (compositions are shown in Table S1) introduced into LC cell, followed by thermal annealing. Detailed protocol for LCPNs fabrication is provided in Section S7.

### 4.4 Shape Encoding of LCPN Films

The prepared LCPN films were cut along or at a specific angle to the alignment direction into ribbons (2 × 10 mm) and placed flat on glass slides for UV irradiation (365 nm). The distance between the light source and the sample was fixed at 15 cm, with a beam diameter of 4 cm and an intensity of 1.2 mW/cm^2^. After irradiation, the films were peeled off from the slides to obtain shape‐encoded LCPN films. To facilitate shape morphing of **LCPN 3**, which has a glass transition temperature above room temperature, the film was removed from the substrate after UV irradiation and heated (70°C for 5 min) to soften the polymer network.

### 4.5 Mechanical Measurements

Elastic modulus of the LCPNs was calculated from the recorded stress‐strain curves using a Modular Force Stage (MFS, Linkam). A constant extension rate of 50 µm/s was used. All polymer ribbons (thickness of 25 µm) were cut along the LC alignment direction to ensure stretching was performed parallel to the molecular orientation. Thickness was selected to ensure that all wavelengths could effectively address the entire volume of the material. To study photo‐generated forces, the ribbons were subjected to a pre‐strain (∼0.5%) and were irradiated with light of different wavelengths (365, 455, and 660 nm) using mounted LEDs (Thorlabs). The photo‐generated force of the film was monitored in real time during irradiation.

## Conflicts of Interest

The authors declare no conflicts of interest.

## Supporting information




**Supporting File 1**: adma74045‐sup‐0001‐SuppMat.pdf.

**Supporting File 2**:adma74045‐sup‐0002‐VideoS1.mp4.

**Supporting File 3**:adma74045‐sup‐0003‐VideoS2.mp4.

**Supporting File 4**:adma74045‐sup‐0004‐VideoS3.mp4.

**Supporting File 5**:adma74045‐sup‐0005‐VideoS4.mp4.

**Supporting File 6**:adma74045‐sup‐0006‐VideoS5.mp4.

**Supporting File 7**:adma74045‐sup‐0007‐VideoS6.mp4.

**Supporting File 8**:adma74045‐sup‐0008‐VideoS7.mp4.

**Supporting File 9**:adma74045‐sup‐0009‐VideoS8.mp4.

**Supporting File 10**:adma74045‐sup‐0010‐VideoS9.mp4.

## Data Availability

All data supporting the findings is provided in the text and the Supporting Information file.
