# Supplementary material for: Decoupling Photochemical and Photothermal Effects Using Molecular Motors Enables Fast, Intelligent, and Life‐Like Motions in Soft Materials
Source: Adv Mater. 2026 Jul 16;38(47):e74045. doi: 10.1002/adma.74045 (PMC13496098; doi:10.1002/adma.74045)
Supplement: Supplementary file 1 [file ADMA-38-e74045-s006.pdf]

Decoupling Photochemical and Photothermal Effects using Photo-efficient Molecular Motors Enables Fast, Intelligent and Life-like Motions in Soft Materials

Guiying Long [1,2], Jinyu Sheng [3], Alexander Ryabchun*[1], Ben L. Feringa*[1,2]

[1] Stratingh Institute for Chemistry, University of Groningen, Nijenborgh 3, 9747 AG Groningen, The Netherlands.

[2] SCNU-UG International Joint Laboratory of Molecular Science and Displays, National Center for International Re-search on Green Optoelectronics, South China Normal University, Guangzhou 510006, China.

[3] College of Chemistry, Chemical Engineering and Materials Science, Soochow University, Suzhou, Jiangsu 215123, China.

*Corresponding authors: Dr. A. Ryabchun (a.ryabchun@rug.nl), Prof. B. L. Feringa (b.l.feringa@rug.nl)

Table of contents

1 General information 1

2 Synthetic routes for the aldehyde motor MDA and MMA 2

3 Characterization of compounds 5

4 ^{1}H-NMR studies of MDA 9

5 UV-vis studies of motor in solution 10

5.1 Photochemical isomerization processes 10

5.2 Thermal Helical Inversion process 10

5.3 Photoisomerization quantum yield 11

6 Preparation of LC cells 13

7 Preparation and characterization of LC mixtures and LCPNs 14

7.1 DSC characterization of LC mixtures as well as LCPNs. 15

7.2 POM images of **LCPN 1-3** 15

8 UV-vis study of the isomerization behavior of MDA and MMA in LCPN films 16

8.1 Photochemical isomerization process in LCPNs 16

8.2 Thermal Helical Inversion process in LCPNs 16

9 TD-DFT Calculation for MDA 17

10 Alignment of molecular motor in LCPNs 18

11 Photo-responsiveness behavior of **LCPN 1-3** 19

12 Shape encoding of **LCPN 1** and **LCPN 2** 20

13 Fatigue test 21

14 Shape stability 22

15 Control experiments 22

16 Light and heat shape encoding process in **LCPN 3** 23

17 UV-vis spectra of LCPN 3 under UV irradiation 24

18 Mechanical properties of LCPNs 24

18.1 Elastic moduli of LCPNs 25

18.2 Photo-generated force 25

19 Optimized geometries of four motor states 29

20 Supplementary videos 1-9: 37

21 References 37

1 General information

All reagents from commercial sources such as Aldrich, TCI, or Merck were used as received. Anhydrous DCM, THF, toluene was obtained from a solvent purification system (MBRAUN SPS systems, MBSPS-800). Flash column chromatography was performed using silica gel (SiO2) purchased from Merck (type 9385, 230-400 mesh) or on a Büchi Reveleris purification system with Büchi cartridges. High resolution mass spectrometry (ESI or APCI-MS) was performed on a LTQ Orbitrap XL spectrometer with ESI or APCI ionization. NMR spectra were recorded on Varian AMX400 (^{1}H: 400 MHz, ^{13}C: 101 MHz), Varian Unity Plus (^{1}H: 500 MHz, ^{13}C: 126 MHz) and Bruker Innova600 (^{1}H: 600 MHz, ^{13}C: 151 MHz) spectrometers. Chemical shifts are quoted in parts per million (ppm) relative to the residual solvent signal (for $CDCl_3$ δ 7.26 for ^{1}H, δ 77.16 for ^{13}C and for CD_2Cl_2 δ 5.32 for ^{1}H, δ 53.84 for ^{13}C). For ^{1}H-NMR spectroscopy, the splitting pattern of peaks is designated as follows: s (singlet), d (doublet), t (triplet), m (multiplet), br (broad), or dd (doublet of doublets). UV-vis absorption spectra were measured on a Hewlett-Packard 8454 diode array spectrometer in a 1 cm quartz cuvette. All the irradiation experiments in solutions were performed using Thorlab LEDs (M365FP1, M455F3). Solvents used for spectroscopic studies were of spectroscopic grade (UVASOL, Merck) or from a solvent purification system. Polarized light absorbance spectra were recorded by AVASPEC-ULS2048CL-EVO (Avantes) spectrometer equipped with Glan-Taylor polarized on a rotational mount (ELL14K, Thorlabs). Irradiation of liquid crystal samples were performed using mounted LEDs (Throlab) at 365 nm, 455 nm and 660 nm. Light intensities were measured with light powermeter PM100A (Thorlabs). Optical imaging and measurements were performed using polarized optical microscope (POM) Eclipse LV100N-POL (Nikon, Japan) and Nis-Element-D software, equipped by a Microscope Hot-stage System HS82 (Mettler Toledo). Differential Scanning Calorimetry (DSC) were carried out on a TA instrument DSC Q1000 in a dry nitrogen atmosphere. All mechanical property measurements of the materials were conducted using Modular Force Stage (MFS) of Linkam. The basic MFS system consists of an MFS stage, a Base, a Force Beam, a T9X System Controller, and LINK software. For thermal imaging IR camera (FLIR, E5) was used.

2 Synthetic routes for the aldehyde motor MDA and MMA

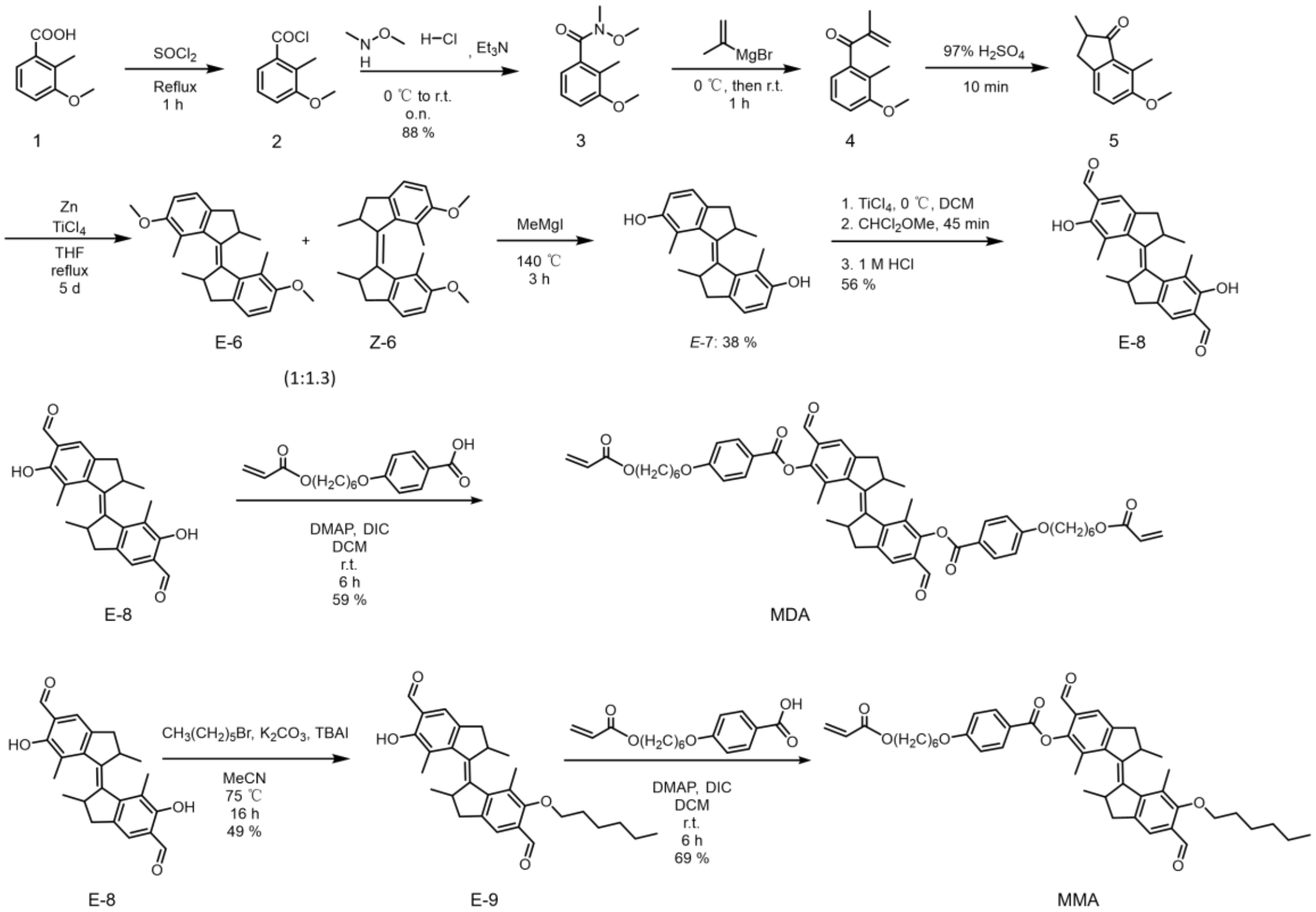

Figure S1 Synthetic routes for the light-driven rotary molecular motors **MDA** and **MMA.**

N,3-dimethoxy-N,2-dimethylbenzamide

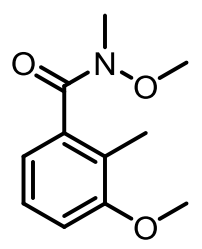

Compound **3** was synthesized following the procedure in ref 1. ^{1}H-NMR (400 MHz, Chloroform-d) δ 7.17 (t, J = 7.9 Hz, 1H), 6.85 (d, J = 8.1 Hz, 2H), 3.83 (s, 3H), 3.37 (m, J = 40.6 Hz, 6H), 2.17 (s, 3H).

1-(3-methoxy-2-methylphenyl)-2-methylprop-2-en-1-one

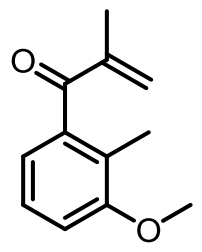

Compound **4** was synthesized following the procedure in ref 1. ^{1}H-NMR (400 MHz, Chloroform-d) δ 7.17 (ddq, J = 8.2, 7.6, 0.6 Hz, 1H), 6.88 (dt, J = 8.2, 0.7 Hz, 1H), 6.80 (dd, J = 7.6, 1.1 Hz, 1H), 5.97 (p, J = 1.4 Hz, 1H), 5.60 (p, J = 0.9 Hz, 1H), 3.84 (s, 3H), 2.10 (d, J = 0.5 Hz, 3H), 2.03 (dd, J = 1.5, 0.9 Hz, 3H).

6-methoxy-2,7-dimethyl-2,3-dihydro-1H-inden-1-one

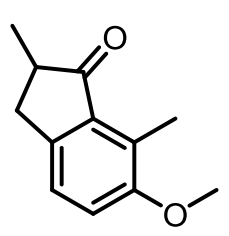

Compound **5** was synthesized following the procedure in ref 1. ^{1}H-NMR (400 MHz, Chloroform-*d*) δ 7.19 (dq, J = 8.2, 0.8 Hz, 1H), 7.07 (d, J = 8.2 Hz, 1H), 3.84 (s, 3H), 3.25 (ddd, J = 16.4, 7.9, 1.0 Hz, 1H), 2.71 – 2.62 (m, 1H), 2.59 (ddd, J = 16.4, 4.3, 1.0 Hz, 1H), 2.53 (s, 3H), 1.28 (d, J = 7.3 Hz, 3H).

6,6'-dimethoxy-2,2',7,7'-tetramethyl-2,2',3,3'-tetrahydro-1,1'-biindenylidene

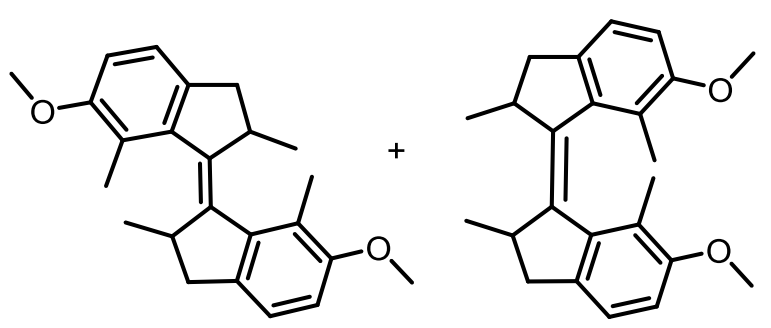

Compound *E*-**6** and *Z*-**6** were synthesized following the procedure in ref 1. ^{1}H-NMR (400 MHz, Chloroform-d) δ 7.02 (dd, J = 14.3, 8.0 Hz, 2H), 6.70 (dd, J = 8.0, 4.8 Hz, 2H), 3.86 (s, 3H), 3.79 (s, 3H), 3.34 (p, J = 6.7 Hz, 1H), 3.18 (dd, J = 14.3, 6.2 Hz,

1H), 2.90 (p, *J* = 6.3 Hz, 1H), 2.74 (dd, *J* = 13.9, 5.7 Hz, 1H), 2.41 (d, *J* = 14.3 Hz, 1H), 2.33 (s, 3H), 2.18 (d, *J* = 13.9 Hz, 1H), 1.41 (s, 3H), 1.09 (dd, *J* = 10.0, 6.6 Hz, 6H).

(*E*)-2,2',7,7'-tetramethyl-2,2',3,3'-tetrahydro-[1,1'-biindenylidene]-6,6'-diol

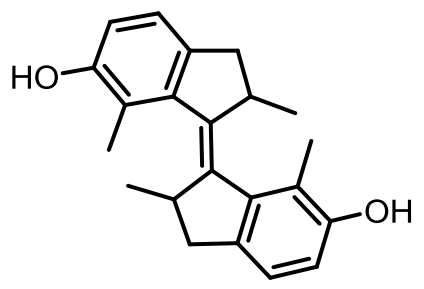

Compound *E*-**7** was synthesized following the procedure in ref 1. ^{1}H-NMR (400 MHz, Chloroform-d) δ 6.93 (d, *J* = 7.8 Hz, 2H), 6.66 (d, *J* = 7.8 Hz, 2H), 4.64 (s, 2H), 2.95 – 2.84 (m, 2H), 2.74 (dd, *J* = 13.9, 5.6 Hz, 2H), 2.36 (s, 6H), 2.18 (d, *J* = 13.9 Hz, 2H), 1.11 (d, *J* = 13.9 Hz, 6H).

(*Z*)-2,2',7,7'-tetramethyl-2,2',3,3'-tetrahydro-[1,1'-biindenylidene]-6,6'-diol

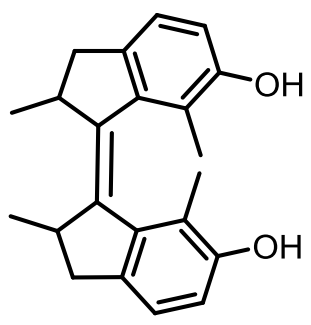

Compound *Z*-**7** was synthesized following the procedure in ref 1. ^{1}H-NMR (400 MHz, Chloroform-d) δ 6.98 (d, *J* = 7.9 Hz, 2H), 6.66 (d, *J* = 7.8 Hz, 2H), 4.47 (s, 2H), 3.33 (p, *J* = 6.7 Hz, 2H), 3.17 (dd, *J* = 14.4, 6.2 Hz, 2H), 2.40 (d, *J* = 14.4 Hz, 2H), 1.46 (s, 6H), 1.08 (d, *J* = 6.8 Hz, 6H).

(*E*)-6,6'-dihydroxy-2,2',7,7'-tetramethyl-2,2',3,3'-tetrahydro-[1,1'-biindenylidene]-5,5'-dicarbaldehyde

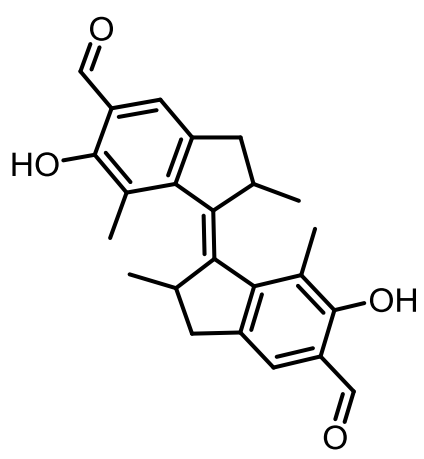

Compound *E*-**8** was synthesized following the procedure in ref 1. ^{1}H-NMR (400 MHz, Chloroform-*d*) δ 11.57 (d, *J* = 0.9 Hz, 1H), 9.83 (d, *J* = 1.5 Hz, 1H), 7.24 (s, 1H), 2.93 (p, *J* = 6.4 Hz, 1H), 2.75 (dd, *J* = 14.1, 5.7 Hz, 1H), 2.38 (s, 3H), 2.31 (d, *J* = 14.1 Hz, 1H), 1.13 (d, *J* = 6.5 Hz, 3H).

(*E*)-6-(hexyloxy)-6'-hydroxy-2,2',7,7'-tetramethyl-2,2',3,3'-tetrahydro-[1,1'-biindenylidene]-5,5'-dicarbaldehyde (*E*-9)

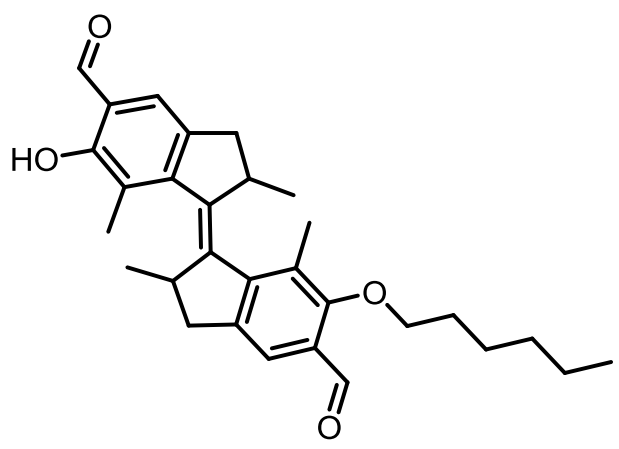

To a solution of *E*-**8** (62 mg, 0.16 mmol) in MeCN (10 mL), K_2CO_3 (69 mg, 0.5 mmol), tetrabutylammonium iodide (184.6 mg, 0.5 mmol) and 1-bromohexane (27 mg, 0.16 mmol) were added. The mixture was stirred at 75 ℃ under nitrogen for 16 h. After cooling to room temperature, the mixture was acidified using a saturated aqueous NH_4Cl solution to pH = 6-7. The product was extracted with EtOAc (3 × 50 mL). The combined organic layers were washed with brine (100 mL), then dried over Na_2CO_3. The solvent was removed in vacuo and the residue was purified by column chromatography (SiO_2, pentane: EtOAc= 14:1) to yield motor *E*-**9** (37 mg, 0.08 mmol, 49%) as yellow solids.

^{1}H-NMR (400 MHz, $CDCl_3$) δ 11.58 (s, 1H), 10.37 (s, 1H), 9.82 (s, 1H), 7.56 (s, 1H), 7.24 (s, 1H), 4.07 (dt, *J* = 9.2, 6.6 Hz, 1H), 3.91 (dt, *J* = 9.2, 6.6 Hz, 1H), 2.91 (dp, *J* = 18.6, 6.4 Hz, 3H), 2.80 – 2.76 (m, 2H), 2.42 (s, 4H), 2.37 (s, 4H), 1.89 (dt, *J* = 15.4, 6.6 Hz, 3H), 1.55 (s, 4H), 1.42 – 1.36 (m, 5H), 1.27 (d, *J* = 11.6 Hz, 2H), 1.14 (d, *J* = 6.5 Hz, 4H), 1.10 (d, *J* = 6.5 Hz, 4H), 0.96 – 0.91 (m, 4H). ^{13}C-NMR (101 MHz, $CDCl_3$) δ 195.8, 190.4, 161.1, 160.2, 149.5, 149.1, 144.6, 143.4, 139.9, 134.4, 128.7, 128.1, 126.5, 123.1, 121.8, 118.6, 76.5, 43.1, 42.9, 40.0, 39.4, 31.7, 30.2, 25.7, 22.6, 18.48, 18.46, 16.3, 15.4, 14.0. HRMS (ESI, m/z) calculated for $C_{30}H_{37}O_4$ $[M^{+}H]^{+}$: 461.2686, found 461.2685.

(*E*)-5,5'-diformyl-6'-(hexyloxy)-2,2',7,7'-tetramethyl-2,2',3,3'-tetrahydro-[1,1'-biindenylidene]-6-yl 4-((6-(acryloyloxy)hexyl)oxy)benzoate (MMA)

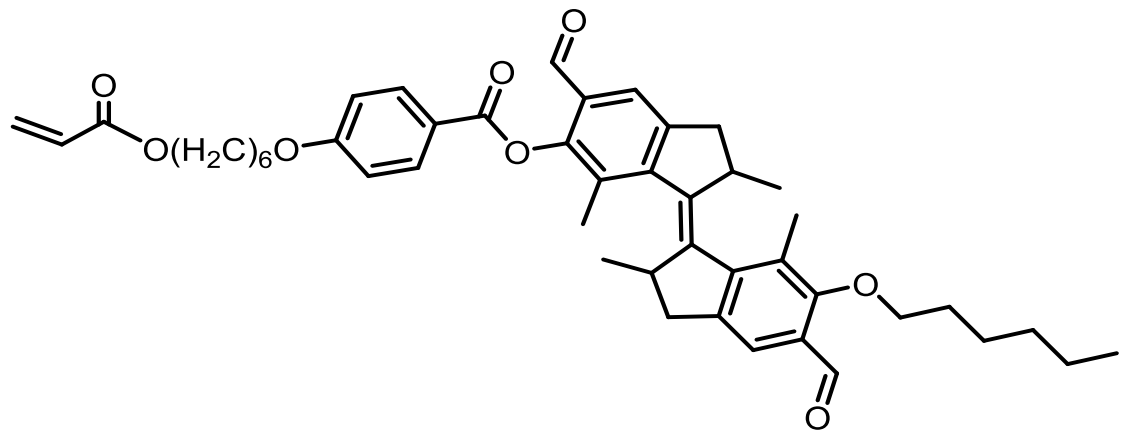

Compound *E*-9 (16 mg, 0.03 mmol, 1.0 equiv.), 4-((6-(acryloyloxy)hexyl)oxy)benzoic acid (15.2 mg, 0.05 mmol, 1.5 equiv.), DMAP (5.2 mg, 0.04 mmol, 1.4 equiv.) were dissolved in dry-DCM (3 mL) under a N_2 atmosphere. N,N′-diisopropylcarbodiimide (DIC) (61 mg, 0.48 mmol, 16.0 equiv.) was added to the solution, and the mixture was stirred at room temperature for 6 h. The mixture was quenched with water (100 mL) and the product was extracted with EtOAc (3 × 10 mL). The combined organic layers were washed with brine, then dried over Na_2SO_4. The solvent was removed in vacuo and the residue was purified by column chromatography (SiO2, pentane: EtOAc=5:1) to yield **MMA** as a yellow solid (15 mg, 0.02 mmol, 59%).

^{1}H-NMR (400 MHz, $CDCl_3$) δ 10.38 (s, 1H), 10.13 (s, 1H), 8.23 (d, *J* = 8.8 Hz, 2H), 7.67 (s, 1H), 7.57 (s, 1H), 7.02 (d, *J* = 8.8 Hz, 2H), 6.41 (dt, *J* = 17.4, 1.2 Hz, 1H), 6.13 (dd, *J* = 17.3, 10.5 Hz, 1H), 5.83 (dt, *J* = 10.4, 1.1 Hz, 1H), 4.19 (t, *J* = 6.6 Hz, 2H), 4.08 (t, *J* = 6.2 Hz, 3H), 3.96 – 3.87 (m, 1H), 2.93 (p, *J* = 6.3 Hz, 2H), 2.83 (ddd, *J* = 20.9, 14.4, 5.7 Hz, 2H), 2.42 (s, 4H), 2.34 (s, 4H), 1.93 – 1.82 (m, 5H), 1.74 (p, *J* = 6.9 Hz, 3H), 1.60 – 1.45 (m, 9H), 1.39 (td, *J* = 7.4, 6.6, 3.1 Hz, 4H), 1.30 – 1.22 (m, 2H), 1.17 (d, *J* = 6.4 Hz, 3H), 1.07 (d, *J* = 6.4 Hz, 3H), 0.96 – 0.91 (m, 4H). ^{13}C-NMR (151 MHz, $CDCl_3$) δ 190.4, 188.7, 166.4, 164.7, 163.9, 161.1, 151.3, 149.0, 148.6, 144.5, 143.1, 142.0, 139.9, 132.6, 130.6, 128.7, 128.6, 128.4, 128.2, 127.4, 123.2, 121.8, 120.5, 114.6, 76.5, 68.2, 64.5, 43.3, 43.1, 40.1, 31.7, 30.2, 29.0, 28.6, 25.8, 25.73, 25.71, 23.5, 22.6, 18.59, 18.56, 16.4, 16.3, 14.1 HRMS (ESI, m/z) calculated for $C_{46}H_{54}O_8Na$ $[M^+Na]^+$: 757.3711, found 757.3701.

(*E*)-5,5'-diformyl-2,2',7,7'-tetramethyl-2,2',3,3'-tetrahydro-[1,1'-biindenylidene]-6,6'-diyl bis(4-((6-(acryloyloxy)hexyl)oxy)benzoate) (MDA)

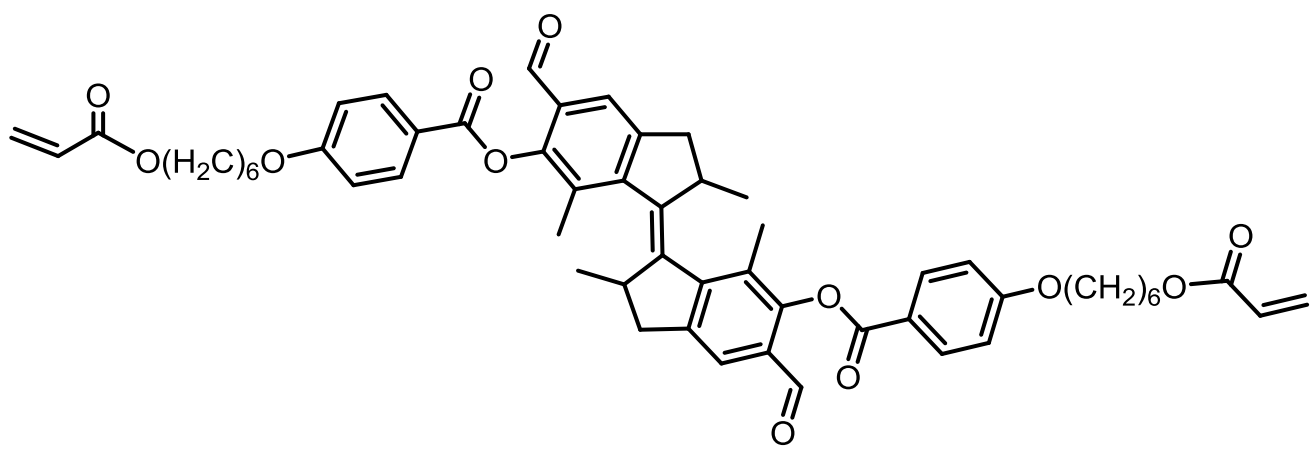

Compound *E*-8 (80 mg, 0.21 mmol, 1.0 equiv.), 4-((6-(acryloyloxy)hexyl)oxy)benzoic acid (156 mg, 0.535 mmol, 2.5 equiv.), DMAP (37 mg, 0.3 mmol, 1.4 equiv.) were dissolved in dry-DCM (5 mL) under a N_2 atmosphere. DIC (430 mg, 3.4 mmol, 16.0 equiv.) was added to the solution, and the mixture was stirred at room temperature for 6 h. The mixture was quenched with water (100 mL) and the product was extracted with EtOAc (3 × 50 mL). The combined organic layers were washed with brine, then dried over Na_2SO_4. The solvent was removed in vacuo and the residue was purified by column chromatography (SiO2, pentane: EtOAc=2:1) to yield M-1 as a yellow solid (136 mg, 0.15 mmol, 69%). ^{1}H-NMR (400 MHz, Chloroform-d) δ 10.13 (s, 1H), 8.23 (d, *J* = 8.9 Hz, 2H), 7.67 (s, 1H), 7.02 (d, *J* = 8.9 Hz, 2H), 6.41 (dd, *J* = 17.3, 1.5 Hz, 1H), 6.13 (dd, *J* = 17.3, 10.4 Hz, 1H), 5.83 (dd, *J* = 10.4, 1.5 Hz, 1H), 4.19 (t, *J* = 6.6 Hz, 2H), 4.08 (t, *J* = 6.4 Hz, 2H), 2.98 (p, *J* = 6.4 Hz, 1H), 2.89 (dd, *J* = 14.7, 5.7 Hz, 1H), 2.40 (d, *J* = 14.6 Hz, 1H), 2.35 (s, 3H), 1.92 – 1.81 (m, 2H), 1.74 (p, *J* = 6.8 Hz, 2H), 1.59 – 1.41 (m, 6H), 1.12 (d, *J* = 6.4 Hz, 3H). ^{13}C-NMR (101 MHz, cdcl3) δ 188.7, 166.3, 164.6, 163.9, 151.2, 148.4, 143.8, 142.1, 132.5, 130.6, 128.6, 128.4, 127.5, 123.3, 120.5, 114.6, 68.2, 64.5, 43.1, 40.2, 29.0, 28.5, 25.71, 25.68, 18.6, 16.3. HRMS (ESI, m/z) calculated for $C_{56}H_{64}O_{12}N$ $[M^+NH_4]^+$: 942.4423, found 942.4422.

3 Characterization of compounds

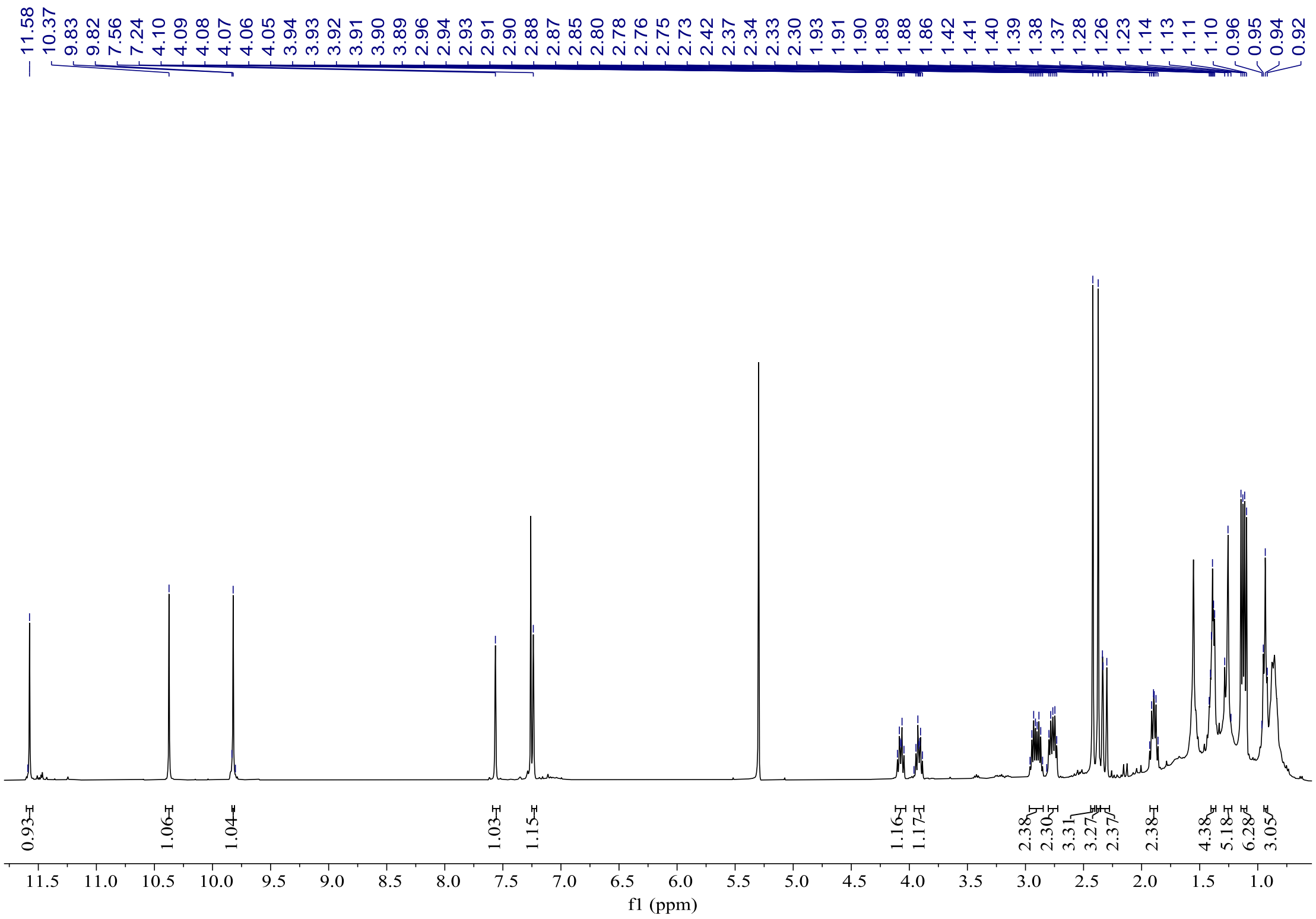

Figure S2 ^{1}H-NMR spectrum of compound *E*-9 in $CDCl_3$.

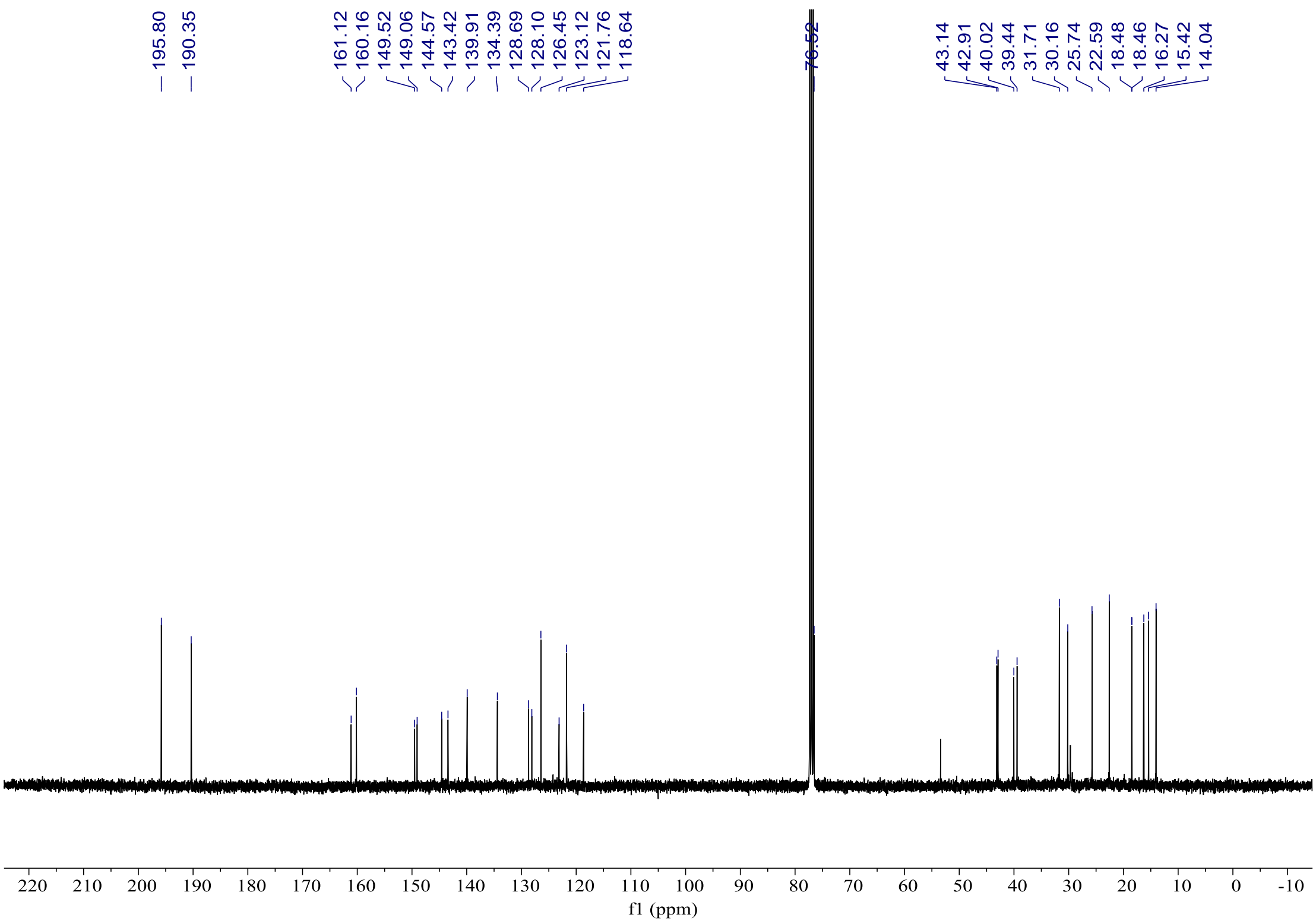

Figure S3 ^{13}C-NMR spectrum of compound *E*-**9** in $CDCl_3$.

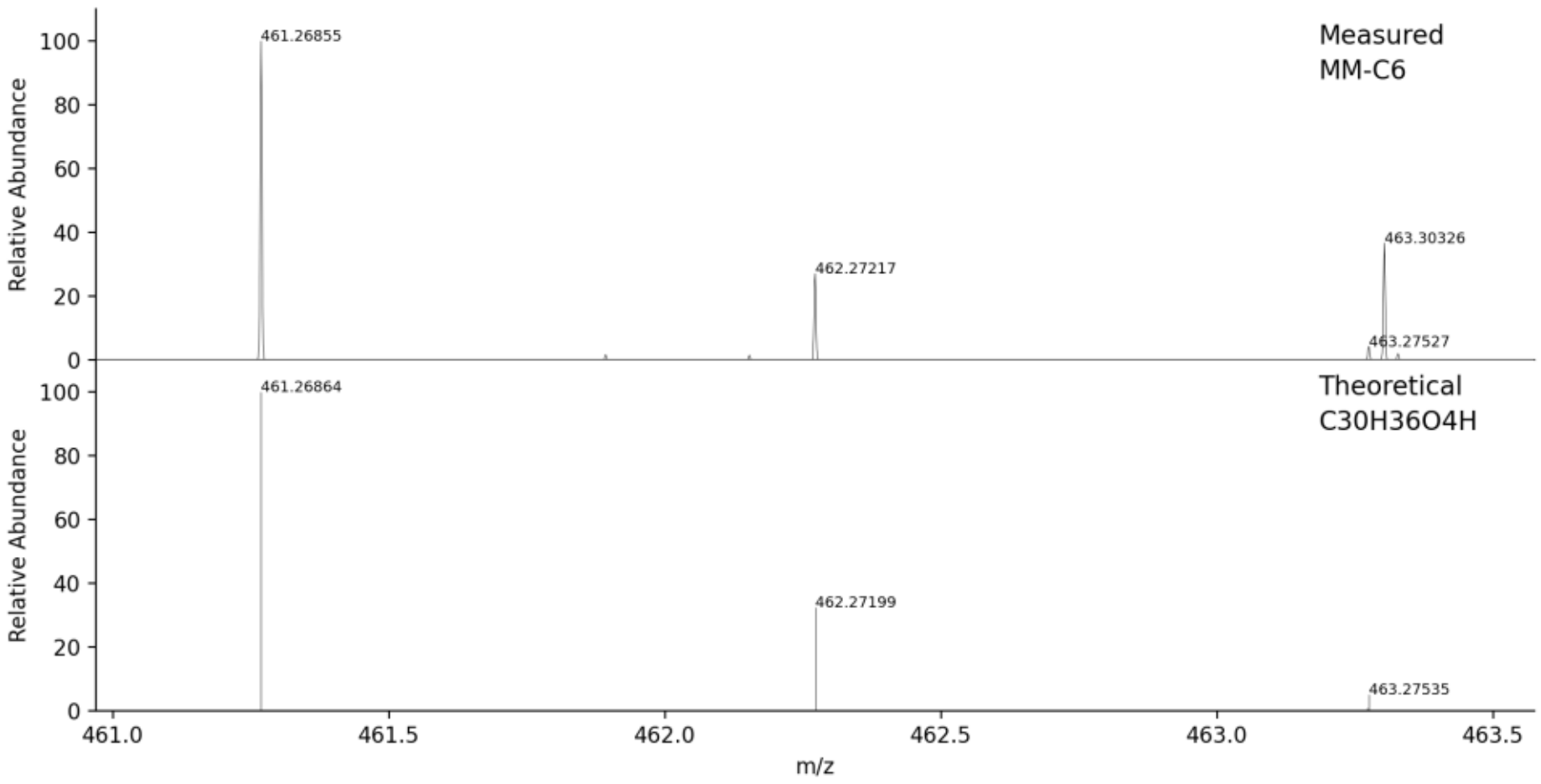

Figure S4 HRMS spectrum of compound *E*-**9**.

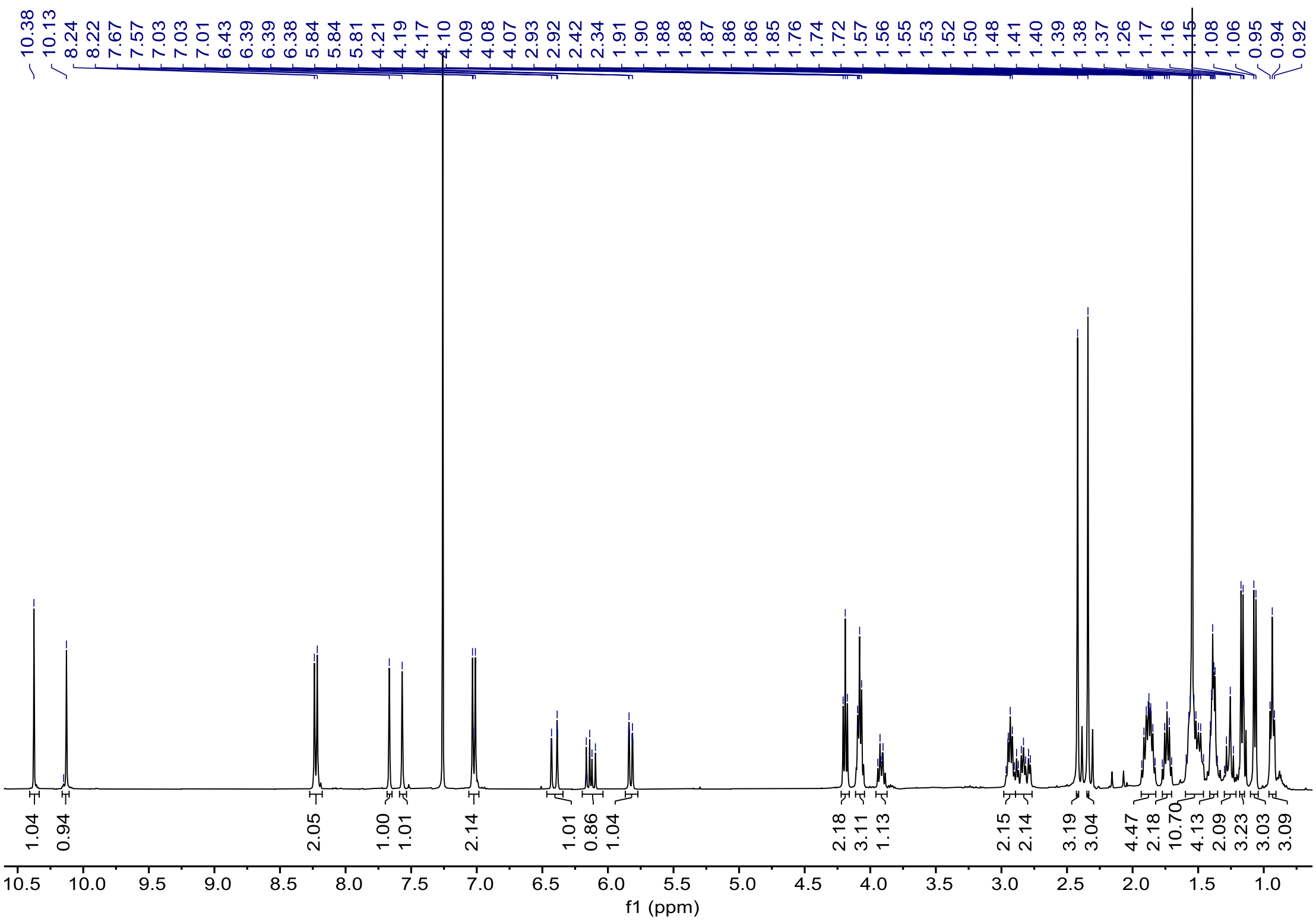

Figure S5 ^{1}H-NMR spectrum of compound **MMA** in $CDCl_3$.

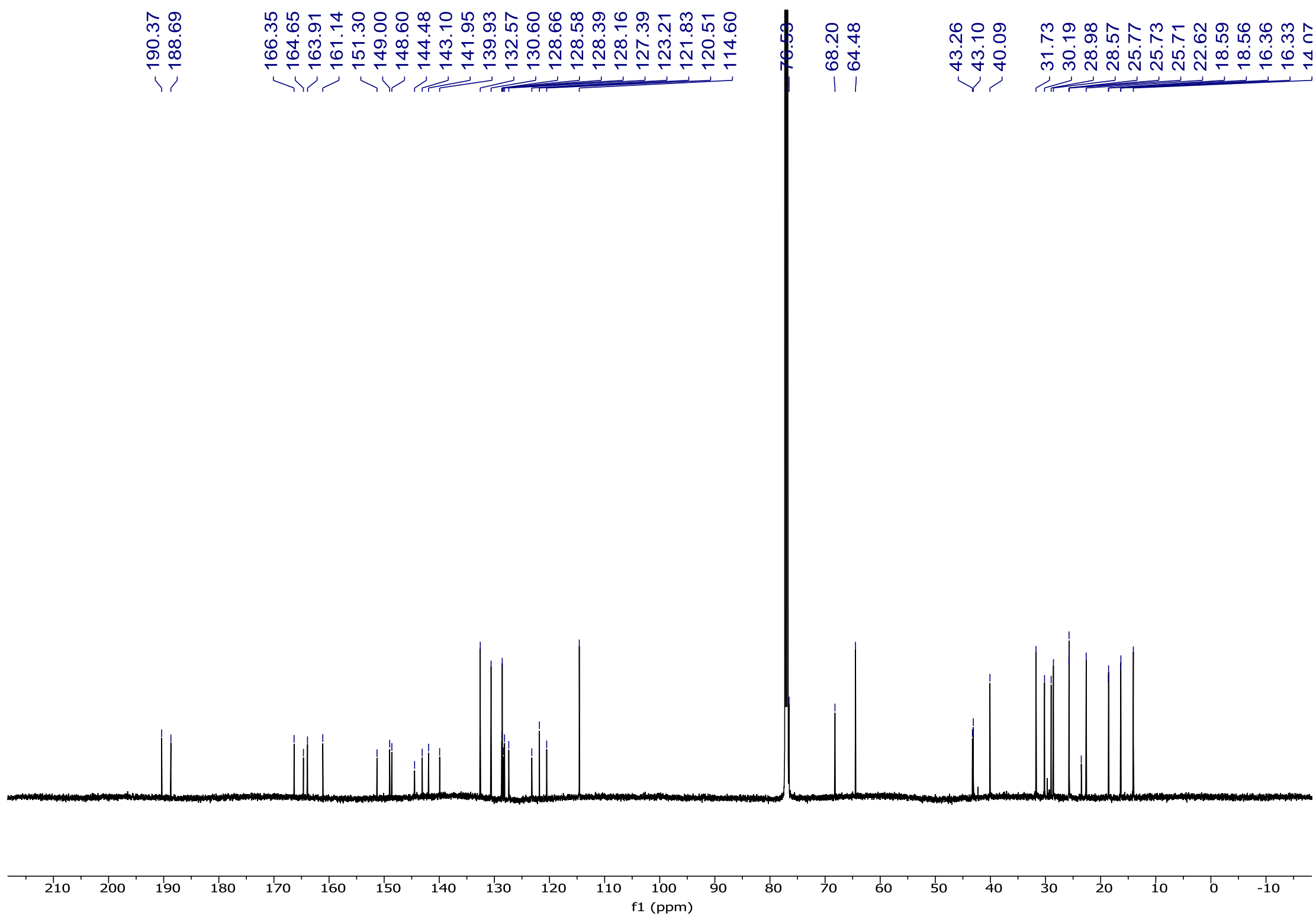

Figure S6 ^{13}C-NMR spectrum of compound **MMA** in $CDCl_3$.

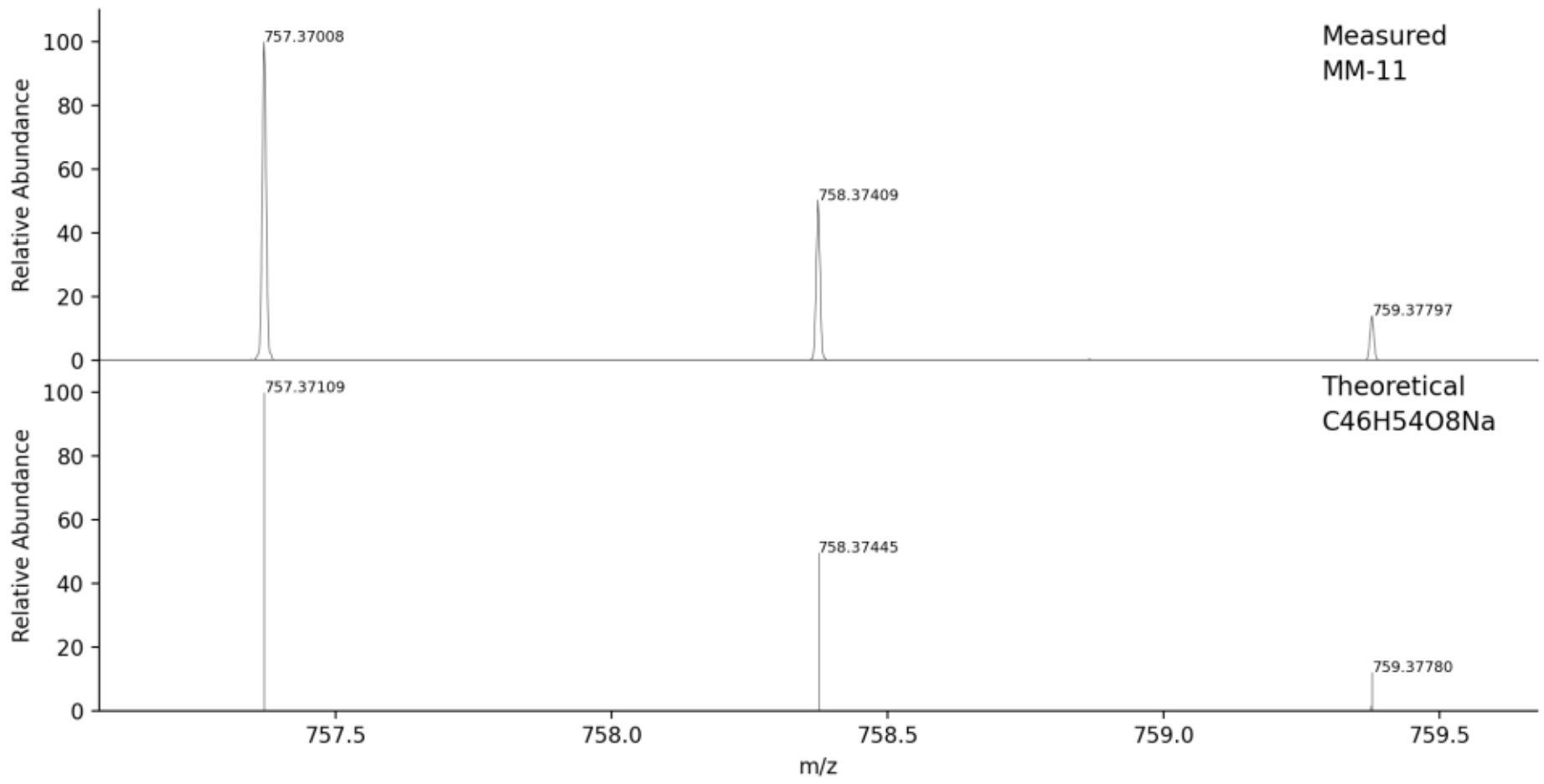

Figure S7 HRMS spectrum of compound **MMA**.

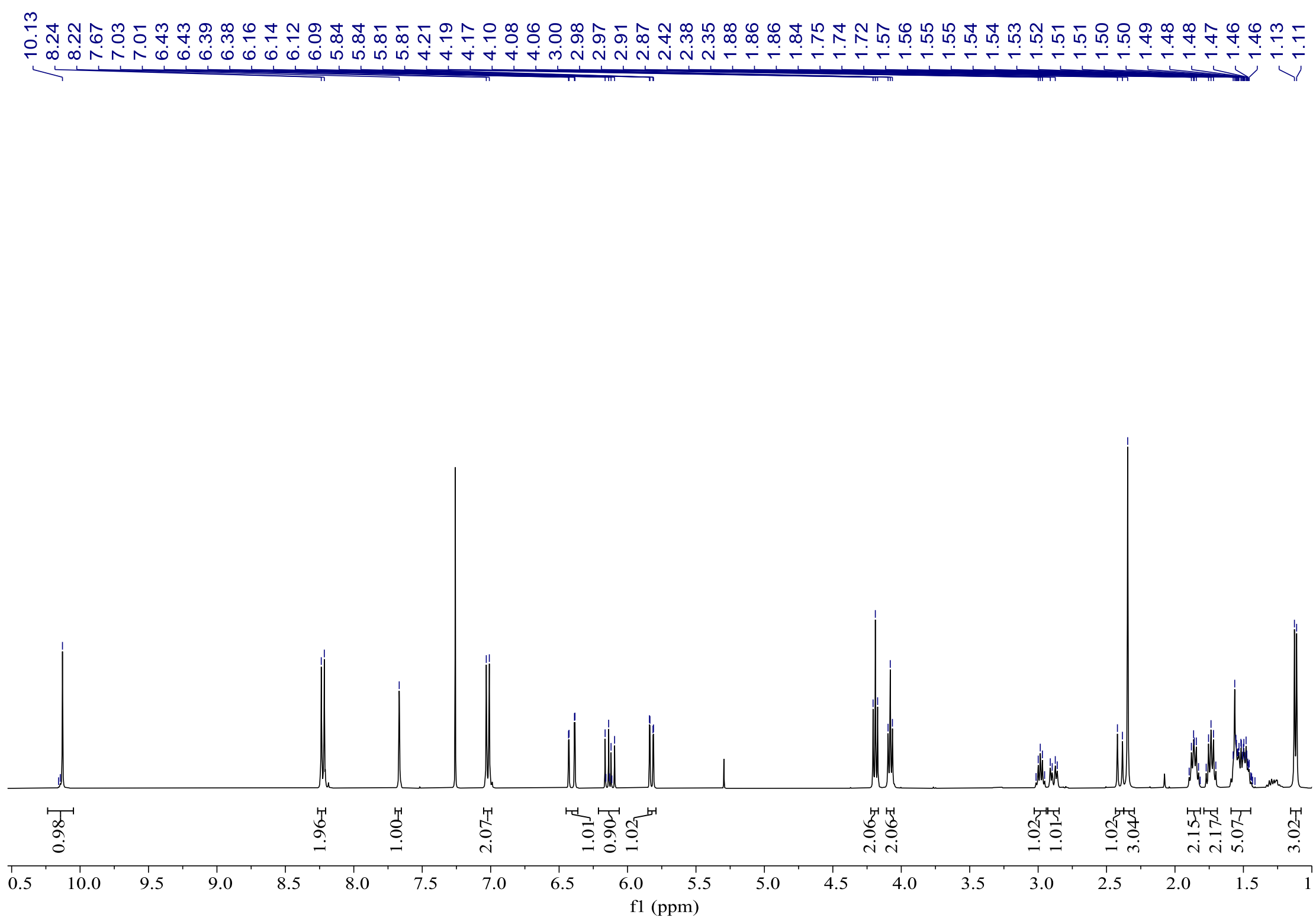

Figure S8 ^{1}H-NMR spectrum of compound **MDA** in $CDCl_3$.

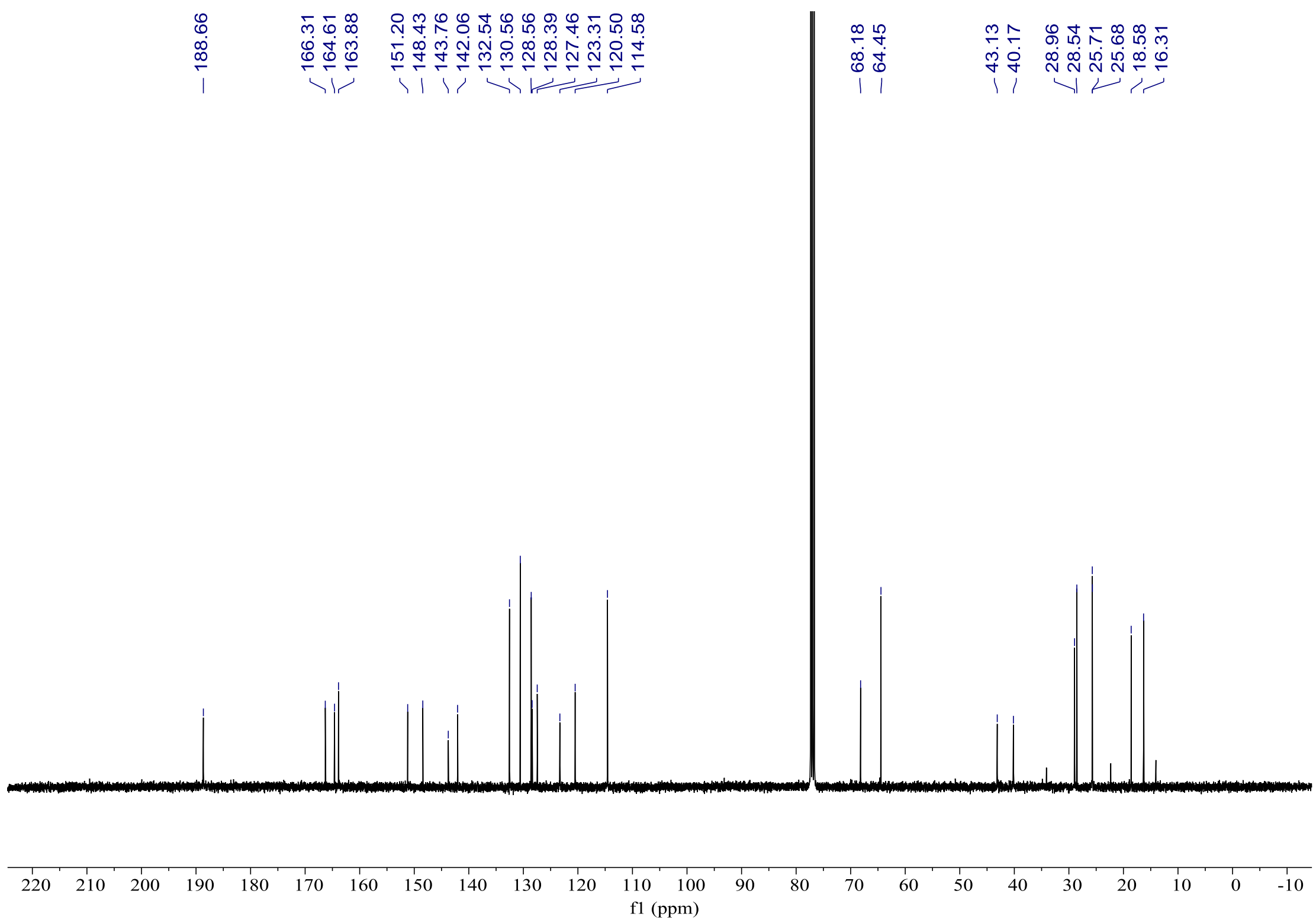

Figure S9 ^{13}C-NMR spectrum of compound **MDA** in $CDCl_3$.

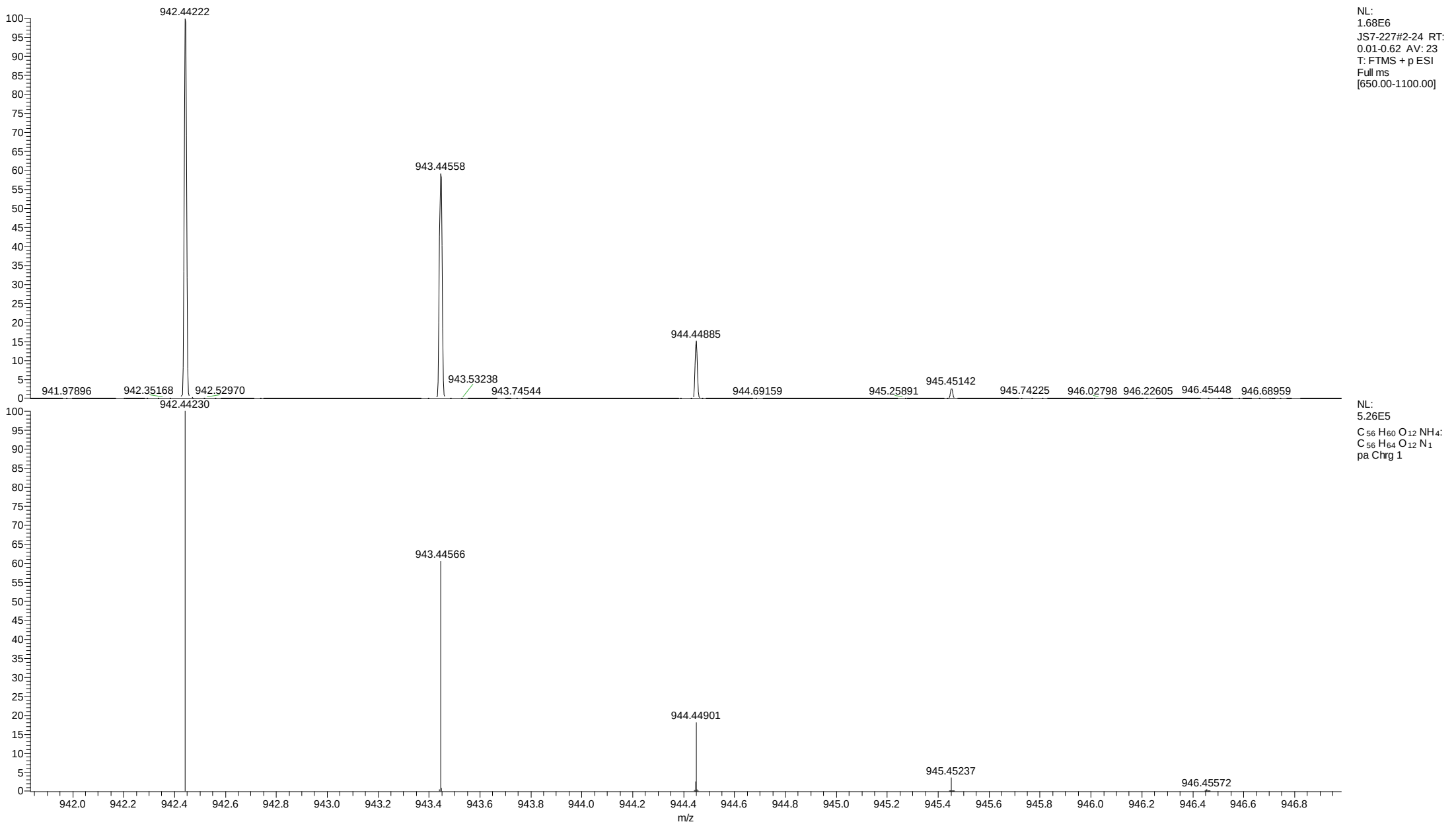

Figure S10 HRMS spectrum of compound **MDA**.

4 ^{1}H-NMR studies of MDA

The prominent characteristic signal peaks of the first-generation motor molecule are the protons Ha, Hb, and Hc. The protons Ha, Hb, and Hc correspond to signals at 3.01 ppm, 2.96 ppm, and 2.48 ppm, respectively, as indicated in orange, due to different chemical environments resulting from the conformation of the five-membered ring. The sample was then irradiated with UV light (λ = 365 nm) at room temperature until it reached the photostationary state (PSS), resulting in a significant change in the spectra, indicating the formation of a new isomer identified as Z_M (green). The new absorptions at 3.53 ppm, 3.36 ppm, and 2.67 ppm were attributed to the Ha, Hb, and Hc protons of Z_M, respectively. The isomer conversion ratio was quantified by integration, yielding a ratio of Z_M to E_S of 97:3. The sample was then kept in the dark at 38 ℃ for 5d, resulting in further shifts of the characteristic absortptions Ha, Hb, and Hc shifted to 3.53 ppm, 3.36 ppm, and 2.67 ppm, respectively (yellow). This indicates that the THI occurred, rotating Z_M to Z_S. Analysis of the spectra revealed that the ratio of Z_S to Z_M exceeded 99:1.

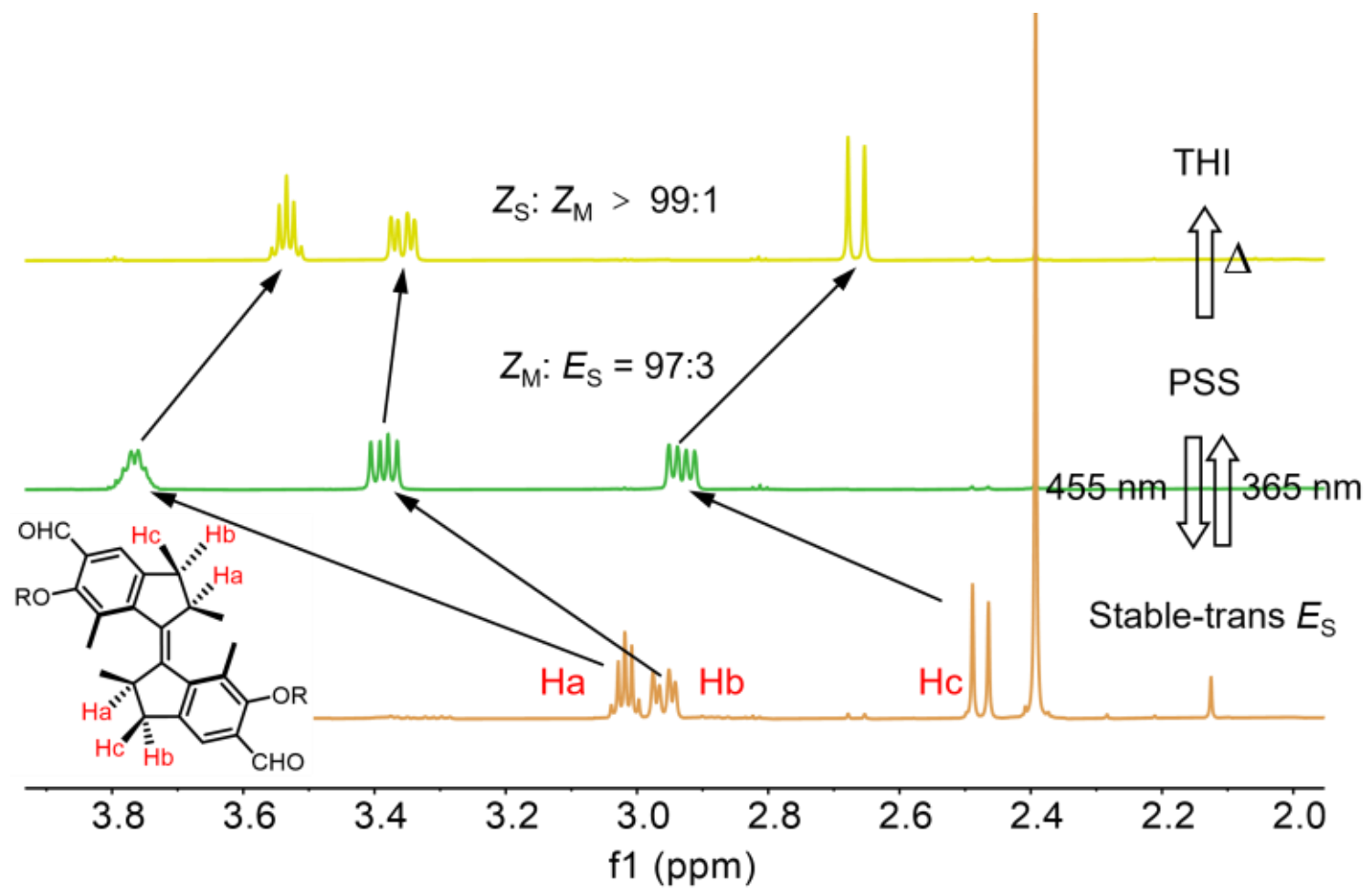

Figure S11 Partial ^{1}H-NMR spectra of MDA in different states.

5 UV-vis studies of motor in solution

UV-vis spectroscopy was used to study the unidirectional four-step rotary cycle of the motor **MDA** and **MMA**, and the 360° rotation included two steps photochemical isomerization process and two steps thermal helical inversion (THI) process (Figure S12 A).

5.1 Photochemical isomerization processes

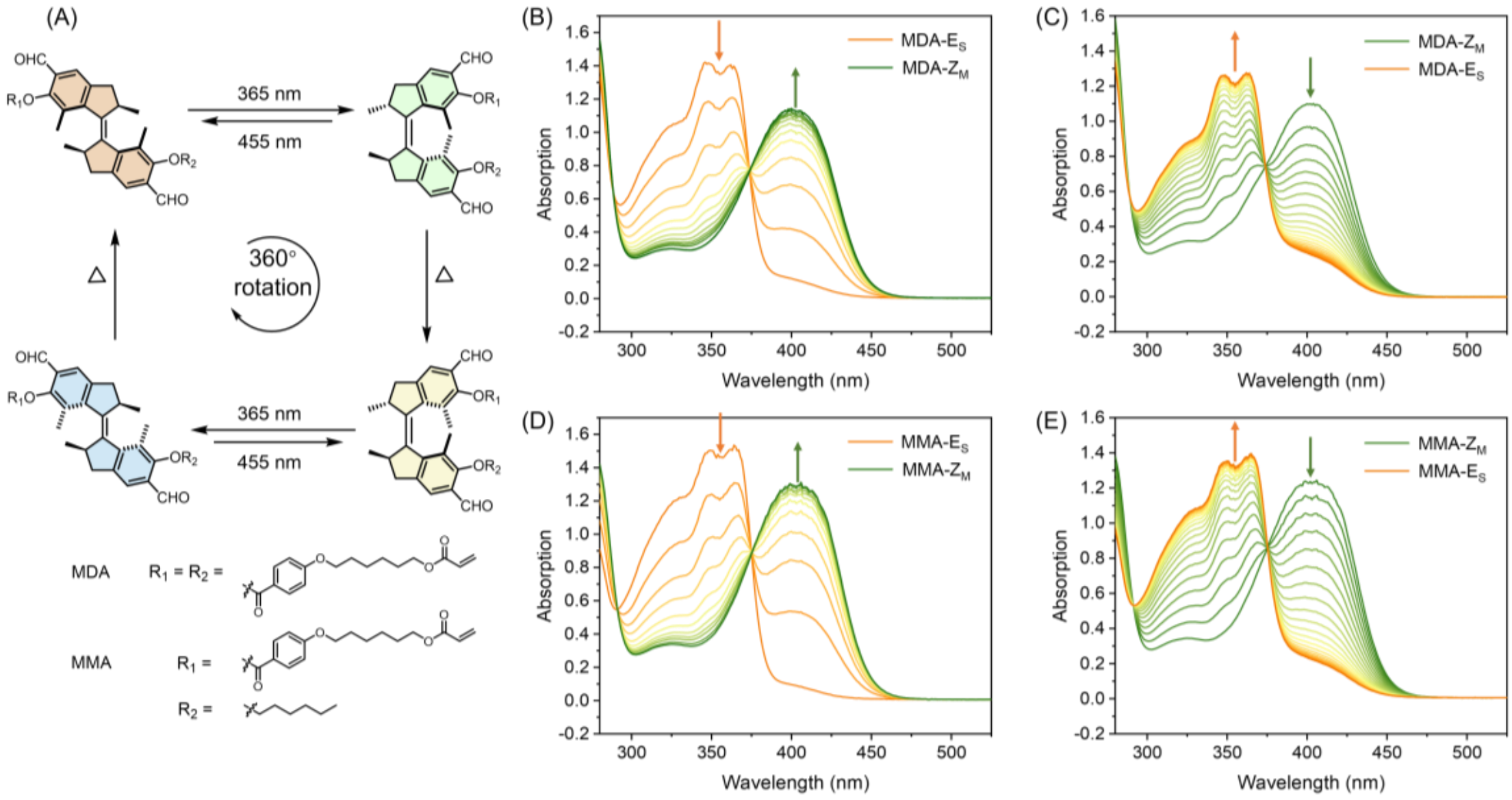

Figure S12 (A) The unidirectional rotary cycle of molecular motor **MDA** and **MMA**. (B) UV-vis spectra changes of E_S-**MDA** upon 365 nm irradiation (5×10^{-5} M, THF, 20 °C). (C) UV-vis spectra of Z_M-**MDA** upon 455 nm irradiation. (D) UV-vis spectra of E_S-**MMA** upon 365 nm irradiation (5×10^{-5} M, THF, 20 °C). (E) UV-vis spectra of Z_M-**MMA** upon 455 nm irradiation.

5.2 Thermal Helical Inversion process

Exposure of the unstable isomers Z_M-**MDA** and Z_M-**MMA** samples to darkness induces the onset of the THI step, leading to the formation of the corresponding stable isomers Z_S-**MDA** and Z_S-**MMA**. Based on this, E_S-**MDA** and E_S-**MMA** were solubilized in toluene at a concentration of 5×10^{-5} M, and UV light was used to convert them into Z_M-**MDA** and Z_M-**MMA**, and then their THI kinetics were measured at different temperatures (70 °C, 75 °C, 80 °C, 85 °C, 90 °C). The absorption of Z_M-**MDA** at 402 nm as well as Z_M-**MMA** at 405 nm are plotted (Figure S13A, C). Using the Eyring equation for the set of rate constants (k) measured at different temperatures, the half-life ($t_{1/2}$) was calculated of the compound **MDA** and **MMA** at room temperature. Furthermore, by plotting ln(k/T) vs 1/T according to the equation, we can calculate $\Delta H^‡$, $\Delta S^‡$, and $\Delta G^‡$. (Figure S13B, D, E)

$$k = \frac{k_B T}{h} e^{-\Delta G^‡/RT}$$

$$\Delta G^‡ = \Delta H^‡ - T\Delta S^‡$$

$$ln\frac{k}{T} = \frac{-\Delta H^‡}{R} \cdot \frac{1}{T} + ln\frac{k_B}{h} + \frac{\Delta S^‡}{R}$$

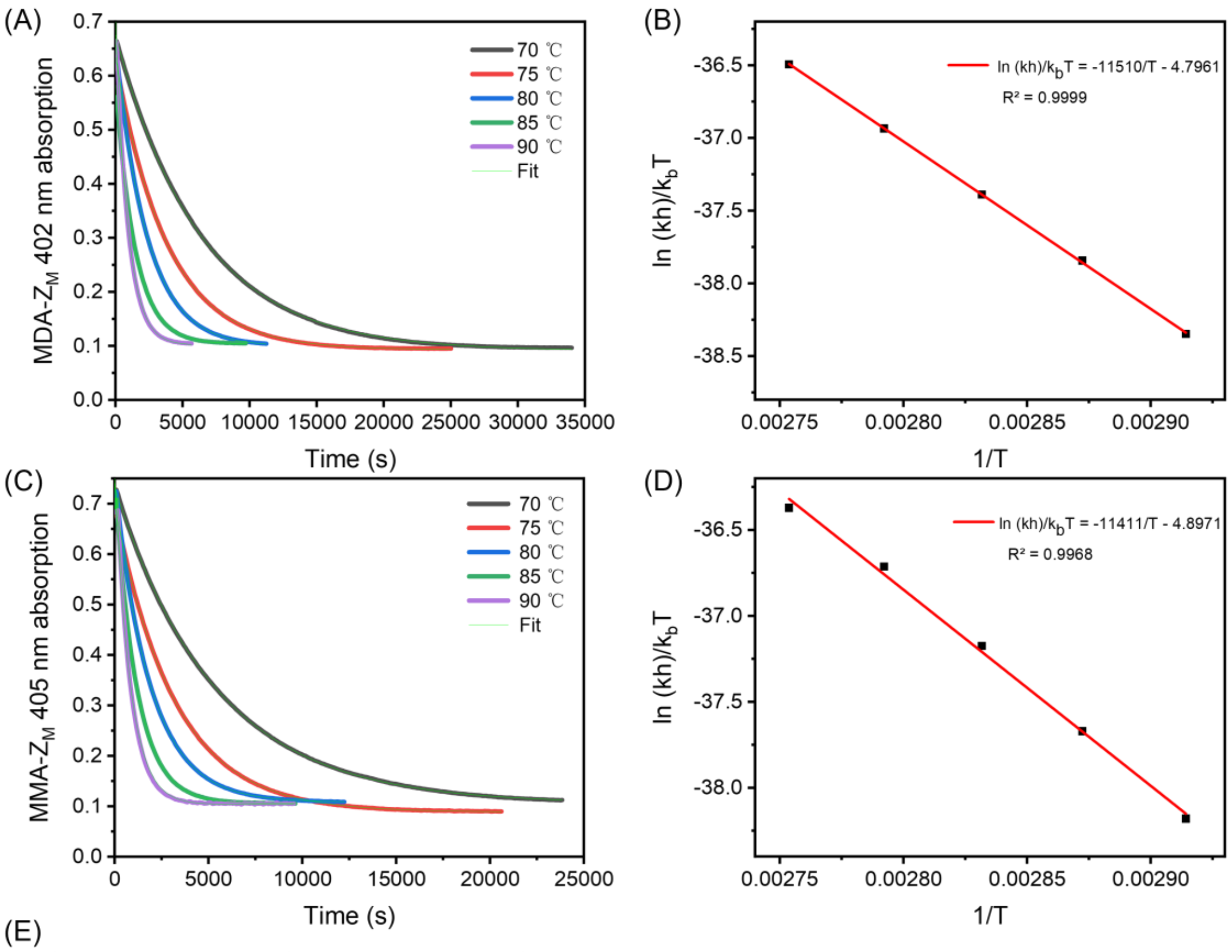

(E)

	$t_{1/2}$ (h)	$\Delta^{\ddagger}G^{o}$ (kJ/mol)	$\Delta^{\ddagger}H^{o}$ (kJ/mol)	$\Delta^{\ddagger}S^{o}$ ($J \cdot K^{-1} \cdot mol^{-1}$)
MDA	437.76	107.37	95.70	-39.88
MMA	345.28	106.81	94.88	-40.72

Figure S13 (A) Time-dependent absorption changes at 405 nm during the THI process of Z_M-**MDA** (5×10^{-5} M, toluene). (B) Eyring plot of THI step from Z_M-**MDA** to Z_S-**MDA** in toluene. (C) Time-dependent absorption changes at 402 nm during the THI process of Z_M-**MMA** (5×10^{-5} M, toluene). (D) Eyring plot of THI step from Z_M-**MMA** to Z_S-**MMA** in toluene. (E) The summary of calculated thermodynamic parameters.

5.3 Photoisomerization quantum yield

In this study, the Ferrioxalate Chemical Actinometry was employed to determine the photon flux[2,3]. A 3 mL aqueous H_2SO_4 solution (0.05 M, prepared by diluting 270 μL of concentrated 98% H_2SO_4 in 100 mL of water) containing $K_3[Fe(C_2O_4)_3]·3H_2O$ (41 mM, 2.014 g in 100 mL) was placed in a 1 cm quartz cuvette and irradiated at 20 °C for a given period in the dark with a 365 nm LED. An aliquot of 100 μL of irradiated solution was then added to a cuvette containing 1.9 mL of an aqueous H_2SO_4 solution (0.5 M, prepared by diluting 2.7 mL of concentrated 98% H_2SO_4 in 100 mL of water), along with phenanthroline (1 g/L, 100 mg in 100 mL) and NaOAc (122.5 g/L). The mixture was allowed to react for 15 min. The cuvette containing the initial solution (an aqueous H_2SO_4 solution with $K_3[Fe(C_2O_4)_3]·3H_2O$) was irradiated again and this step was repeated to get at least 5 exposures. The absorption at λ=510 nm was measured and compared to an identically prepared non-irradiated sample. The concentration of $[Fe(phenanthroline)_3]^{2+}$ complex was calculated using its molar absorptivity (ε =

11100 M^{-1} cm^{-1}) and considering the dilution. The quantity of Fe^{2+} ions (expressed in mmol) was plotted versus time (expressed in seconds), and the formation rate of Fe^{2+} ions at the given wavelength was obtained from the slope of the linear fit to the equation y=ax+b. This rate can be converted into the photon flux (I) by dividing it by the quantum yield of the $[Fe(phenanthroline)_3]^{2+}$ complex at the wavelength of the used LED and by the probability of photon absorption at the given wavelength (Φ^{365nm}=1.20) [4] of the Fe^{3+} complex (approximately 1 in all cases due to the full absorption mode). The calculated molar photon flux values were: $I^{365nm}=2.93\times10^{-5}$ mE/s (current=300 mA), $I^{365nm}=3.97\times10^{-5}$ mE/s (current=500 mA).

These values were derived for single-wavelength light sources. Since quantum yield may exhibit wavelength dependence, this factor represents a potential source of error in the measurements. The solution of the E_S-**MDA** (concentration of 2.52×10^{-5} M in toluene, achieving an absorbance of approximately 0.7 at the irradiation wavelength) was irradiated with UV light at a wavelength of 365 nm, leading to the formation of the Z_M-**MDA**. Spectral data were collected by monitoring the absorption changes at the irradiation wavelength. Subsequently, the decay was fitted by the ODE solver present in COPASI, employing the Levenberg-Marquardt algorithm with randomized initial conditions. Quantum yield was calculated by the below equation:

$$\frac{d[A]}{dt} = \frac{\Phi_{trans-stable \rightarrow cis-metastable} \cdot I \cdot \beta_{trans-stable}}{N_A \cdot V} + \frac{\Phi_{cis-metastable \rightarrow trans-stable} \cdot I \cdot \beta_{cis-metastable}}{N_A \cdot V}$$

Where I is the molar photon flux, N_A is Avogadro's constant, V is the total volume of the irradiated solution (2 mL), and β is the fraction of photons absorbed by either the E_S-**MDA** or Z_M-**MDA** isomer, determined using the PSS ratio and the corresponding molar extinction coefficients for each isomer. To obtain physically reasonable results, the boundaries for the quantum yield values were fixed between 1×10^{-6} and 1. In the testing of the **MDA** motor, different LED currents (300 mA and 500 mA) were selected and tested 4 times in total to ensure data reliability. The average values obtained are presented in Figure S14.

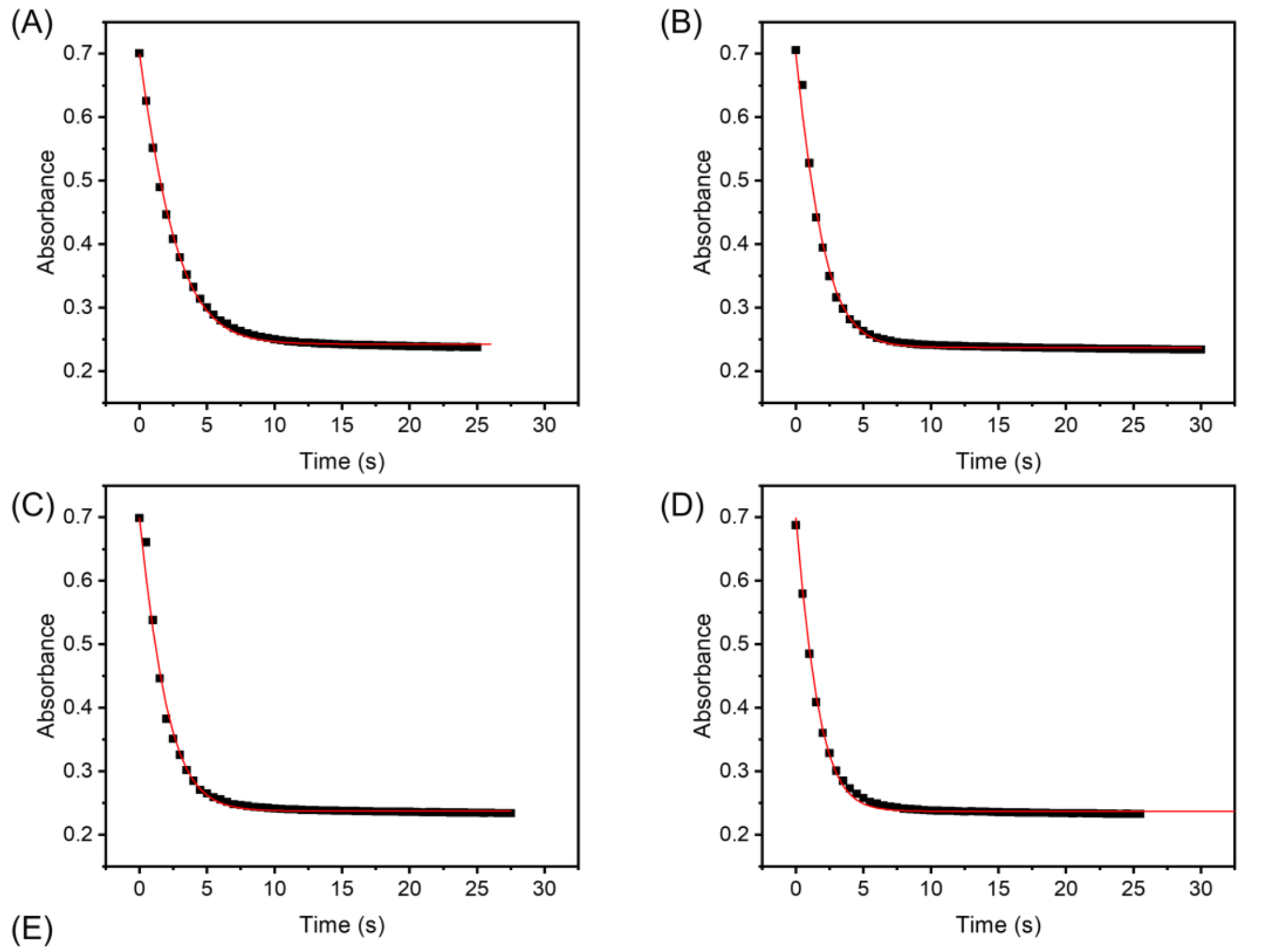

(E)

Motor	$\varepsilon_{trans\text{-}stable}$ ($M^{-1}cm^{-1}$)	$\varepsilon_{cis\text{-}metastable}$ ($M^{-1}cm^{-1}$)	PSS_{365} Trans-stable : cis-metable	$\Phi_{trans\text{-}stable \rightarrow cis\text{-}metastable}$	$\Phi_{cis\text{-}metastable \rightarrow trans\text{-}stable}$
MDA	27743.5	8755.8	3 : 97	71.2%±5.4%	9.2%±1.7%

Figure S14 (A-D) Evolution of the absorption at 365 nm during the irradiation of **MDA** in toluene at 365 nm. The red line represents the fit obtained with the ODE solver from COPASI. Current: (A) 300 mA, (B-D) 500 mA. (E) Molar extinction coefficients of E_S-**MDA** and Z_M-**MDA** at 365 nm, PSS of **MDA** at 365 nm, and photoisomerization quantum yield data in toluene.

6 Preparation of LC cells

Square glass pieces (2 cm × 2 cm) were cut and sequentially washed with anhydrous ethanol and acetone, then wiped with dust-free paper and blown dry with N_2 to clean the glass surface. The glass substrates were spin-coated with a polyimide (PI) solution promoting planar LC alignment for 1 min at 5000 rpm, followed by placing them on a hot stage at 80 °C for 1 h, and then heated to 180 °C overnight to form the PI planar alignment layer. To prepare the PI homeotropic alignment layer, the cleaned glass substrate was first placed on a hot stage at 60 °C to warm it, and then the glass substrate was spin-coated with a homeotropic PI solution (5000 rpm, 1 min), followed by annealing at 80 °C for 1 h, and backing at 200 °C for 1 h for obtaining the glass substrate with the homeotropic alignment layer. Then the planar substrates can be rubbed using a velvet cloth to form an alignment layer. Two glass substrates with the same alignment layer are glued together in parallel with UV-curable adhesive containing 25 μm spacers to obtain a planar LC cell. Moreover, the glass substrate with the rubbed planar alignment layer is directly glued to the homeotropic glass substrate with a spacer of 25 μm to obtain a splayed LC cell.

7 Preparation and characterization of LC mixtures and LCPNs

The clearing points of different liquid crystal mixtures were determined by polarized optical microscopy (POM) and differential scanning calorimetry (DSC) to select the polymerization temperature (Figure S15A-C). The **LCPN 1** mixture was filled into the splayed LC cell at 45 ℃, the **LCPN 2** mixture into the planar LC cell at 50 ℃, and the **LCPN 3** mixture into the planar LC cell at 60 ℃, and the control sample had the same conditions as the **LCPN 2**, and the Azo-**LCPN 1** was the same as the **LCPN 1**. the chemical structures of components and their ratios are shown in Table S1. The mixtures were left at the corresponding temperatures for 30 min to improve molecular alignment, and then polymerized by irradiating with blue light (λ= 470 nm) for 10 min. After the polymerization, the LC cells were annealed at 100 °C for 10 min, cooled to room temperature and opened. the free-standing polymer films were obtained by pilling them of the glass substrates. DSC was used to determine the glass transition temperature (Tg) of the LCPNs. The DSC curve showed that the peak at the position of the clearing point before polymerization disappeared, indicating that the polymer was fully polymerized, as shown in Figure S15D-F. Under POM we can clearly observe that when the cross polarizers are oriented at 45°/135° in respect to rubbing direction of PI coatings, the samples appear bright and homogeneous, while when they are oriented at 0°/90°, the samples appear dark, which correlates with the unidirectional LC alignment of the networks, as shown in Figure S16.

	Structures	LCPN 1 (wt%)	LCPN 2 (wt%)	LCPN 3 (wt%)	Control sample	Azo-LCPN 1 (wt%)
	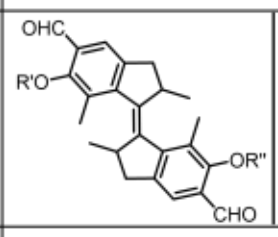 Azo 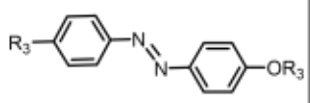	3 (MDA)	3 (MDA)	3 (MDA)	3 (MMA)	3 (Azo)
C6M	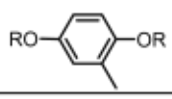	22	22	47	22	22
C6BP	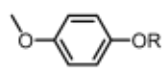	50	50	30	50	50
C6BP6	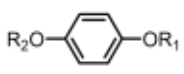	15	20	-	20	15
C6BPN	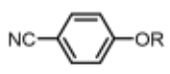	-	-	20	-	-
DB14	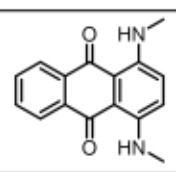	1	1	1	1	1
E7	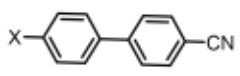	10	5	-	5	10
Irg 819	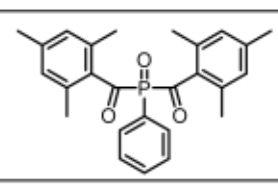	1	1	1	1	1
R_1 = R = 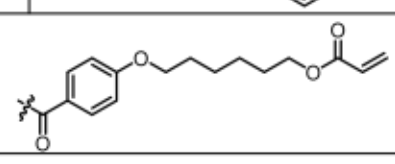 R_2 = R_3 = 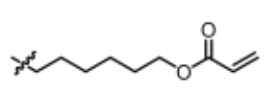 MDA: R'= R''= R; MMA: R'= R, R''= R_2						

Table S1 Structure as well as compositions of liquid crystal photopolymerizable mixtures.

7.1 DSC characterization of LC mixtures as well as LCPNs.

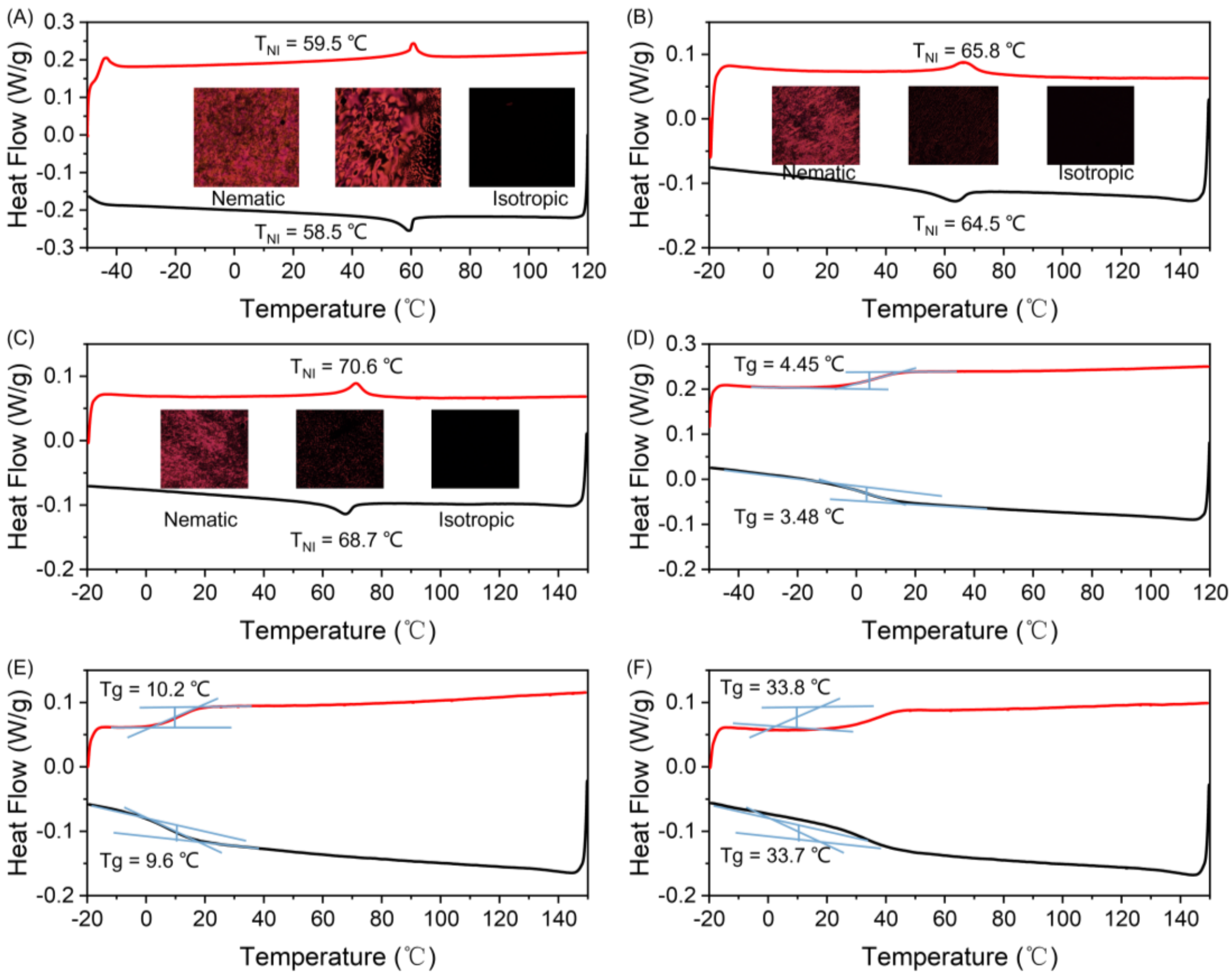

Figure S15 DSC curves of liquid crystal mixtures before and after polymerization. (A-C) DSC curves of liquid crystal mixtures **LCPN1-3** before polymerization. From the curves, the clearing points are 59 °C, 65 °C, and 69 °C, respectively. (D-F) DSC curves of liquid crystal polymers **LCPN 1-3**. The Tg from the curves are 4 °C, 10 °C, and 34 °C, respectively. The red line is the heating curve with a rate of 5 °C/min and the black line is the cooling curve with a rate of -5 °C/min.

7.2 POM images of LCPN 1-3

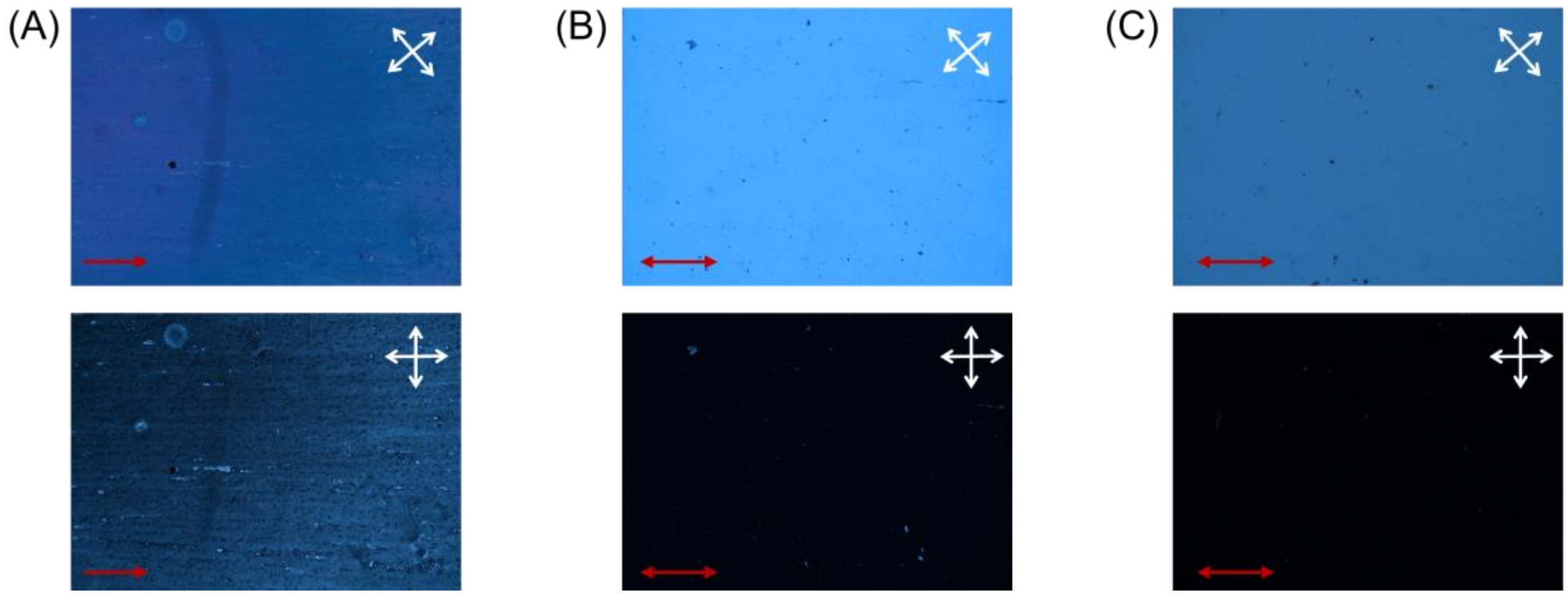

Figure S16 POM images of liquid crystal polymer networks. (A) splay aligned **LCPN 1** film, (B) planar aligned **LCPN 2** film, and (C) planar aligned **LCPN 3** film. The white arrows represent the direction of the polarizer and

the analyzer. The red arrow represents the alignment direction of the liquid crystal molecules. The red unidirectional arrow represents a homeotropic alignment on the other side.

8 UV-vis study of the isomerization behavior of MDA and MMA in LCPN films

Based on the rotational behavior of molecular motors in solution, we have also carried out the same studies of motors in liquid crystal networks, thus demonstrating that the function of molecular motors is maintained in polymers.

8.1 Photochemical isomerization process in LCPNs

The liquid crystal mixtures of **LCPN 2** and the control sample were filled into quartz liquid crystal cells with a parallel alignment and a thickness of 2.5 μm. After polymerization, the samples were annealed at 100 ℃ for 10 min to obtain the desired samples. The prepared LCPN2 and control samples were exposed to UV light at room temperature and the changes in their UV-vis absorption spectra were recorded (Figure S17).

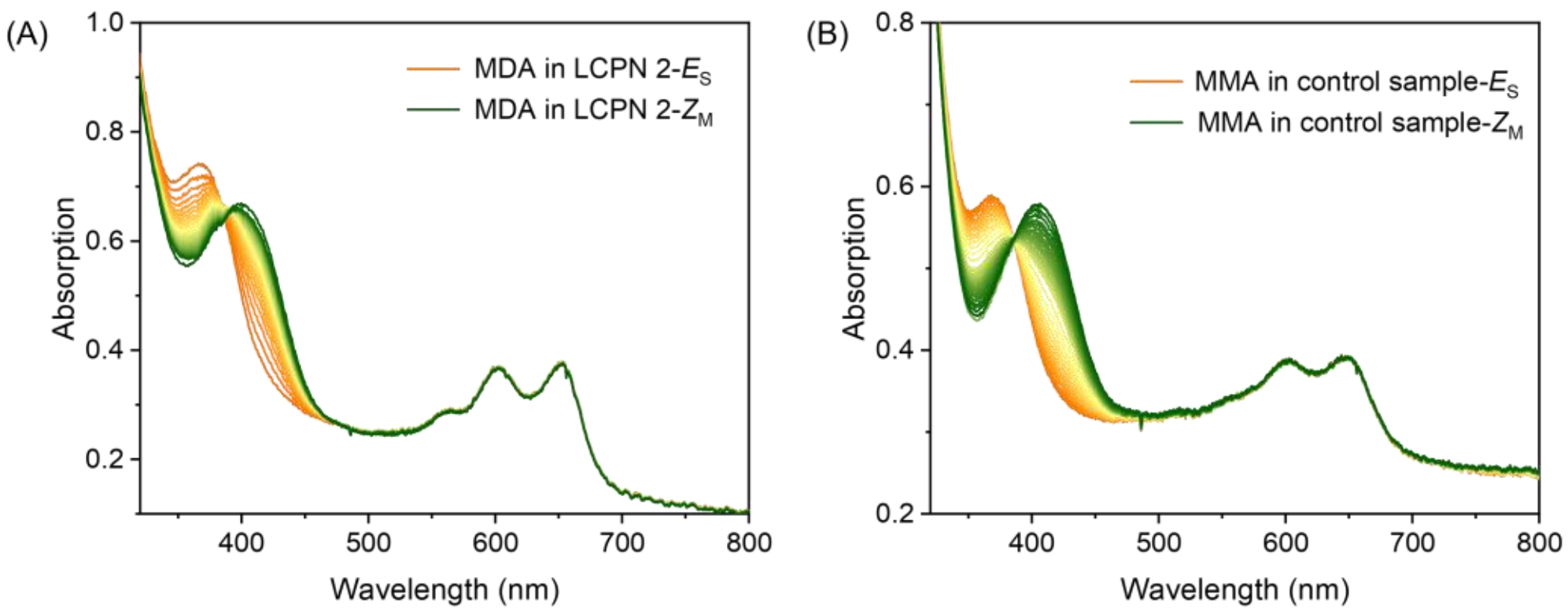

Figure S17 UV-vis absorbance spectra of (A) **LCPN 2** and (B) control sample upon 365 nm irradiation. The absorption band of **LCPN 2** and the control film red-shifted from 365 nm to 400 nm under UV irradiation, similar to the motor's behavior in solution, confirming its rotational motion in the LCN film. In contrast, the absorption bands of DB 14 at 602 nm and 653 nm remained unchanged, indicating its insensitivity to UV light.

8.2 Thermal Helical Inversion process in LCPNs

The THI kinetics of the motors in LCN were measured at different temperatures (100 ℃, 110 ℃, 120 ℃, 130 ℃). The UV-vis absorption spectra of **MDA** as well as **MMA** in the films at 400 nm were recorded (Figure S18A, C). Using the Eyring equation for the set of rate constants (k) measured at different temperatures, we can calculate the half-life ($t_{1/2}$) of **MDA** and **MMA** in the films at room temperature. In addition, by plotting ln(k/T) vs 1/T according to the equation, the $\Delta^‡H°$, $\Delta^‡S°$, and $\Delta^‡G°$ values were calculated (Figure S18B, D, E).

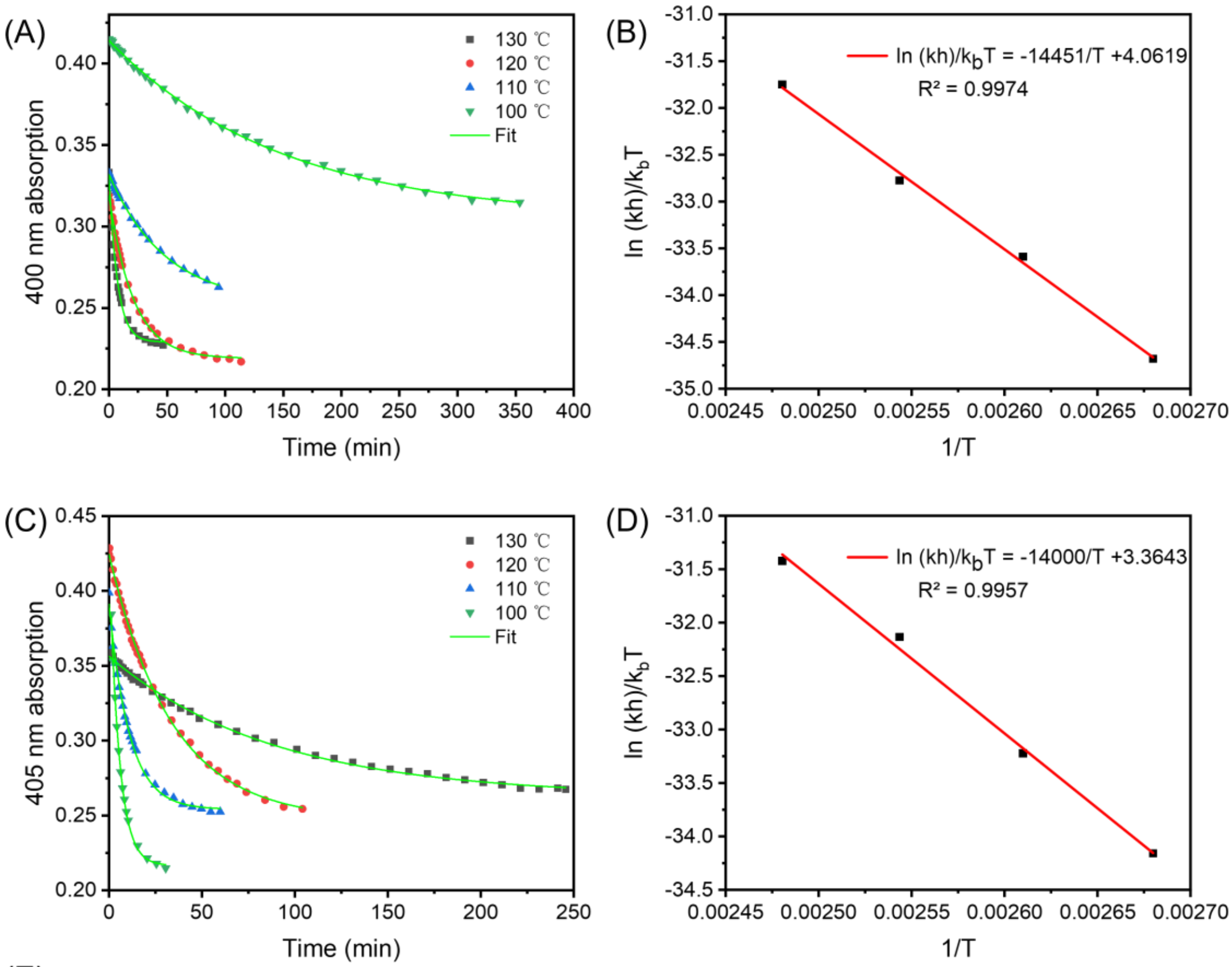

(E)

	$t_{1/2}$ (h)	$\Delta^{\ddagger}G^{o}$ (kJ/mol)	$\Delta^{\ddagger}H^{o}$ (kJ/mol)	$\Delta^{\ddagger}S^{o}$ (J·K^{-1}·mol^{-1})
MDA in LCPN	1451.68	110.25	120.15	33.77
MMA in LCPN	626.94	108.21	116.41	27.97

Figure S18 (A) Time-dependent absorption changes of Z_M-**MDA** in LCPN film at 400 nm during THI process. (B) Eyring plot of THI step from Z_M-**MDA** to Z_S-**MDA** in LCPN. (C) Time-dependent absorption changes of Z_M-**MMA** in LCPN film at 400 nm during THI process. (D) Eyring plot of THI step from Z_M-**MMA** to Z_S-**MMA** in LCPN. (E) The summary of calculated thermodynamic parameters.

9 TD-DFT Calculation for MDA

The ground state structures of motor were optimized for each of the forms involved in the rotary cycle, by using density functional theory with ωB97X-D4 [5] hybrid functional and def2-TZVPP basis set. We performed a vertical excitation calculation for each of the optimized geometries, by using the time-dependent density functional theory method (TD-DFT) without Tamm-Dancoff approximation, with the same functional and basis set using the conductor-like polarizable continuum solvent model CPCM(acetonitrile)[6], considering 30 singlet transitions. Orientations of $S_0 \rightarrow S_1$ transition dipole moments (TDMs) (Figure S19A) were extracted from those calculations to interpret the spectral data on motor alignment in the liquid crystal network (Figure S18). All calculations have been performed using the Orca 5.0.4 package [7]. Two ethoxy substituents of the motor core were used to simply the calculations. Coordinates of four optimized geometries of motor' states are provided in Table S4.

10 Alignment of molecular motor in LCPNs

We investigated the alignment of molecular motor in three states in liquid crystal polymer network by means of linearly polarized light UV-vis spectroscopy. This technique allows estimating the orientation of the electronic transition dipole moment (TDM) in the motor (calculated by TD-DFT, see above) and, consequently, of the molecular motor itself with respect to the orientation of liquid crystal molecules. The 2.5 µm thin **LCPN 1** was prepared inside a quartz LC cell promoting unidirectional molecular alignment (rubbed polyimide layers). E_S-**MDA** as well as the isomerized Z_M-**MDA**, Z_S-**MDA** in the LCPN. For measuring the polarized light absorbance, the spectrometer was equipped with a polarizer (Glan-Taylor prism) mounted on a rotatable stage to change the angle of polarization of the light in 10-degree increments. As shown in Figure S19B, the polarized absorbance of the molecular motors in the E_S state was significantly larger parallel to the LC director than in the perpendicular direction, indicating that the TDM of molecular motor was mainly aligned along the LC alignment axis as evidenced by the polar plot (Figure S19C). TDM of Z_M-**MDA** obtained by 365 nm irradiation, was found to be mainly aligned perpendicular to the LC orientation. TDM in this state of the motor is rotated approx. 90° in respect to rigid motor's halves indicating their orientation along the LC alignment with similar dichroism value as for E_S-**MDA** (Figure S19A-C). Interestingly, Z_S-**MDA** reorients orthogonally probably by the constrained from the main polymer chains of the network, however this realignment was not profound as indicated by very low dichroism value.

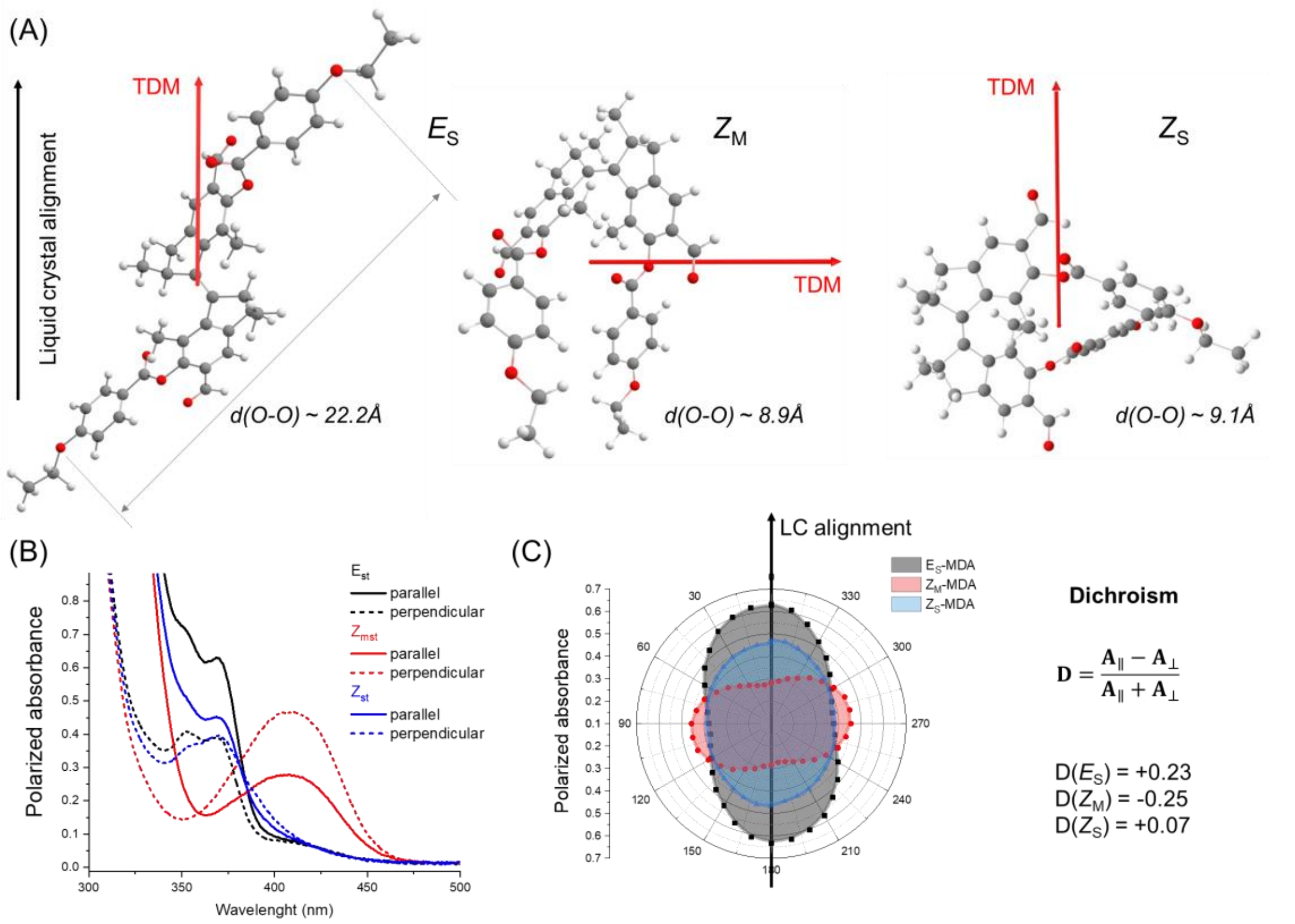

Figure S19 (A) Optimized geometries of molecular motors in different states. Transition dipole moment (TDM) is positioned in respect to LC alignment direction based on polarized absorbance data. After photoisomerization, the distance between the oxygen atoms at both ends of the **MDA** decreases from 22.2 Å to 8.9 Å, and following thermal isomerization, it adjusts to 9.1 Å. (B) Polarized absorbance spectra of molecular motors E_S-**MDA** in unidirectionally aligned **LCPN 1** films before (black line), after exposure to 365 nm UV light (red line) and after subsequent thermal helix inversion (blue line). (C) Angular dependence of polarized absorbance as well as

dichroism values of motor **MDA** in different states. (D) Dichroism was calculated by given equation, where $A_{||}$ and $A_{\perp}$ are the absorbance of light polarized parallel and perpendicular to the direction of the liquid crystal alignment. $|D| = 0$ indicates the absence of any order, and at the other extreme $|D| = 1$ indicates perfect order.

11 Photo-responsiveness behavior of LCPN 1-3

The prepared unidirectionally aligned **LCPN 1**, **LCPN 2**, and **LCPN 3** were cut into strips (2 mm × 10 mm) along the alignment axis. These strips were then laid flat on a glass slide and subjected to UV irradiation. The UV irradiation conditions were as follows: the distance between the UV light source and the samples was fixed at 15 cm, the diameter of the light spot was 4 cm, and the light intensity was 1.2 mW/cm². We investigated the light-driven deformation of the film under UV irradiation in detail. The deformation monitored through the curvature of the ribbon progresses until it reaches the maximum. This maximum curvature arises due to the inhomogeneous stress distribution across the thickness of the ribbon, which is caused by the gradient in the concentration of the Z_M-**MDA** isomer. With continued irradiation, the light penetrates deeper into the material, leading to further isomerization of **MDA** throughout the entire thickness. Once the motor reaches PSS, the associated stress gradient diminishes, resulting in the unbending of the ribbon back to its original shape (Figure S20).

The curvature of the films was recorded by removing them from the slides at every certain time of irradiation. From Figure S21, it can be seen that the curvature of the films **LCPN 1-3** increases to reach the maximum followed by the decrease until almost initial values. During the recording of **LCPN 3**, after each UV irradiation, the sample was removed from the glass slide and placed on a hot stage at 70 ℃ for a certain period. This process softens the network, allowing the accumulated stresses to be exposed into the shape change (Tg of **LCPN 3** is higher than r.t.). The time required for maximum deformation of **LCPN 1-3** is 41 s, 180 s, and 550 s, which can provide a reference for the irradiation duration in the subsequent experiments (Figure S21).

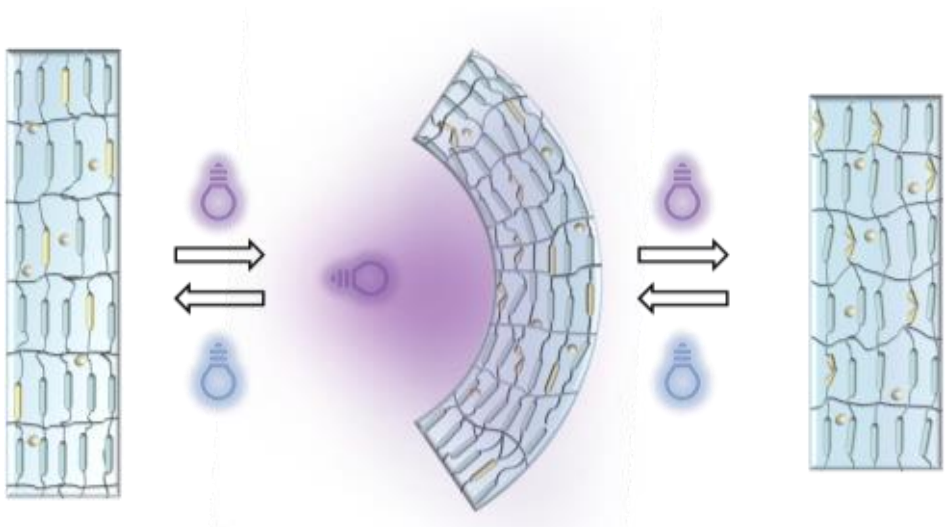

Figure S20 Schematic illustration of the principle of deformation of a photoresponsive liquid crystal film when it is stimulated by light.

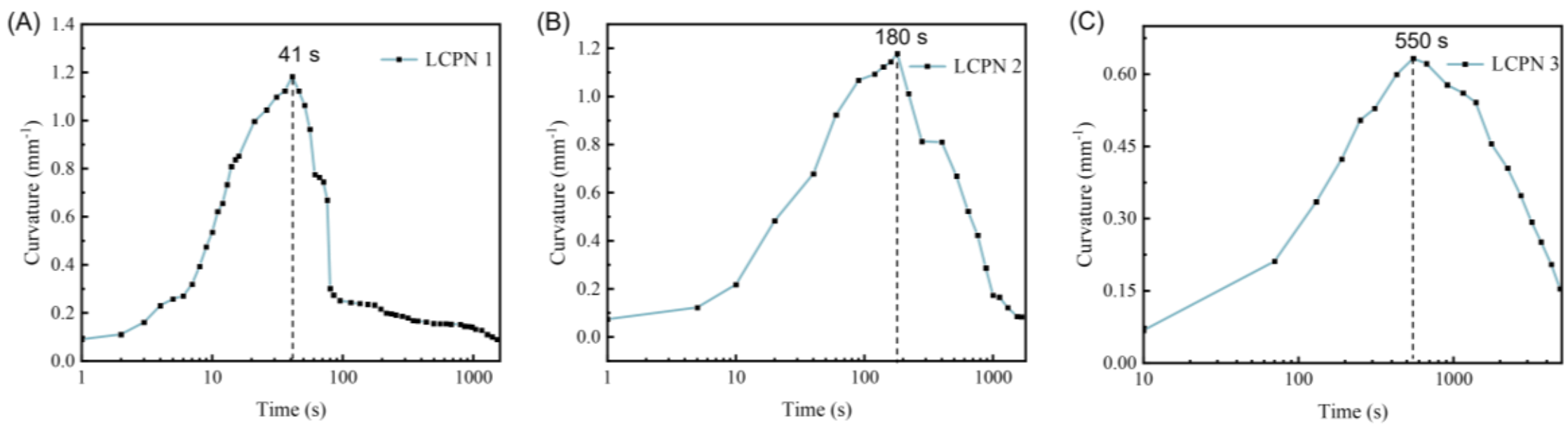

Figure S21 Evolution of the curvature of LCPN films upon irradiation with 365 nm UV light. The intensity of the UV light was 1.2 mW/cm^2.

12 Shape encoding of LCPN 1 and LCPN 2

The photopolymerized LCPN film was cut at a certain angle (θ) with respect to the molecular alignment to form the desired polymer ribbon (Figure S22A). This approach allows us to obtain ribbons with molecular alignment and therefore anisotropic mechanical properties tilted with respect to the long axis of the ribbons which gives an additional leverage to control the polymer shape. For a **LCPN 2** with parallel alignment, when the cutting direction is aligned with the alignment direction (θ = 0°), the film predominantly undergoes uniaxial contraction under external stimuli, resulting in bending deformation. In contrast, when the cutting direction forms an angle with the alignment direction, the contraction within local regions becomes non-uniform, leading to a twisted deformation of the entire film. Figure S22B illustrates the shape coding of ribbons with cutting angles ranging of [0°, 90°] ∪ [-90°, 0°] under UV light. A positive shear angle is defined as the angle at which the ribbon is cut from top to bottom relative to the alignment direction on the low-modulus side, determined by the direction of the light source during polymerization. When the cutting angle θ is within the range of [0°, 90°], the ribbons exhibit a left-handed (LH) helical shape, with the helical pitch increasing as the angle increases. When the cut angle is in the range of [-90°, 0°], the ribbons display a right-handed (RH) helical shape, with the helical pitch decreasing as the angle increases. In these cases, the side exposed to UV light is always on the inside of the springs and experiences larger curvature than the outer side. This phenomenon arises because cutting at different angles alters the stress distribution and deformation mode of the film under light stimulation, affecting the coupling of local anisotropic contraction, thereby leading to helically deformed structures with varying degrees of twisting. At θ~0° or ±90° (the white dotted area in the Figure S22B, the ribbons exhibited achiral shapes. Importantly, each shape of the ribbons can be erased under 455 nm light irradiation, allowing for easy reconfiguration.

In the splay LC configuration, liquid crystal molecules experience unidirectional planar orientation on one of the confining surfaces while on the opposite surface LC molecules align perpendicularly (or homeotropically) to it as schematically depicted in (Figure S22C). Gradual variation in molecular alignment across the film thickness fingerprints mechanical properties in the way that the side of the film with planar alignment has higher elastic modulus compared to opposite side with homeotropic alignment. And the homeotropic side is always positioned on the inner curvature and undergoes the greatest deformation due to its lower modulus. Therefore, the uneven stress distribution originating from splay alignment after photopolymerization induces non-uniform deformation when the film is removed from a solid support, further demonstrating the complex interplay between LC configuration and mechanical behavior. Consequently, when the **LCPN 1** film is cut at different angles (defined as the angle observed from top to bottom relative to the direction of parallel alignment), the internal stress gradient resulting from the molecular arrangement across the thickness caused asymmetric stress release, resulting in various shapes depending on the cutting angle. Notably, a helical shape can be therefore encoded without any chiral molecules involved, where the symmetry breaking element is the mechanical strain gradient across the film thickness. Figure S22D shows that when the cutting angle is in the range of [0°, 90°], the ribbons exhibit a LH helical shape, with the helical pitch increasing as the angle increases. In contrast, for the cutting angle falls within the range of [90°, 180°], the ribbons adopt a RH helical shape, where the helical pitch decreases as the angle increases. At θ = 0° or 90°, the ribbons exhibit achiral shapes and usually shows bending or folding motion.

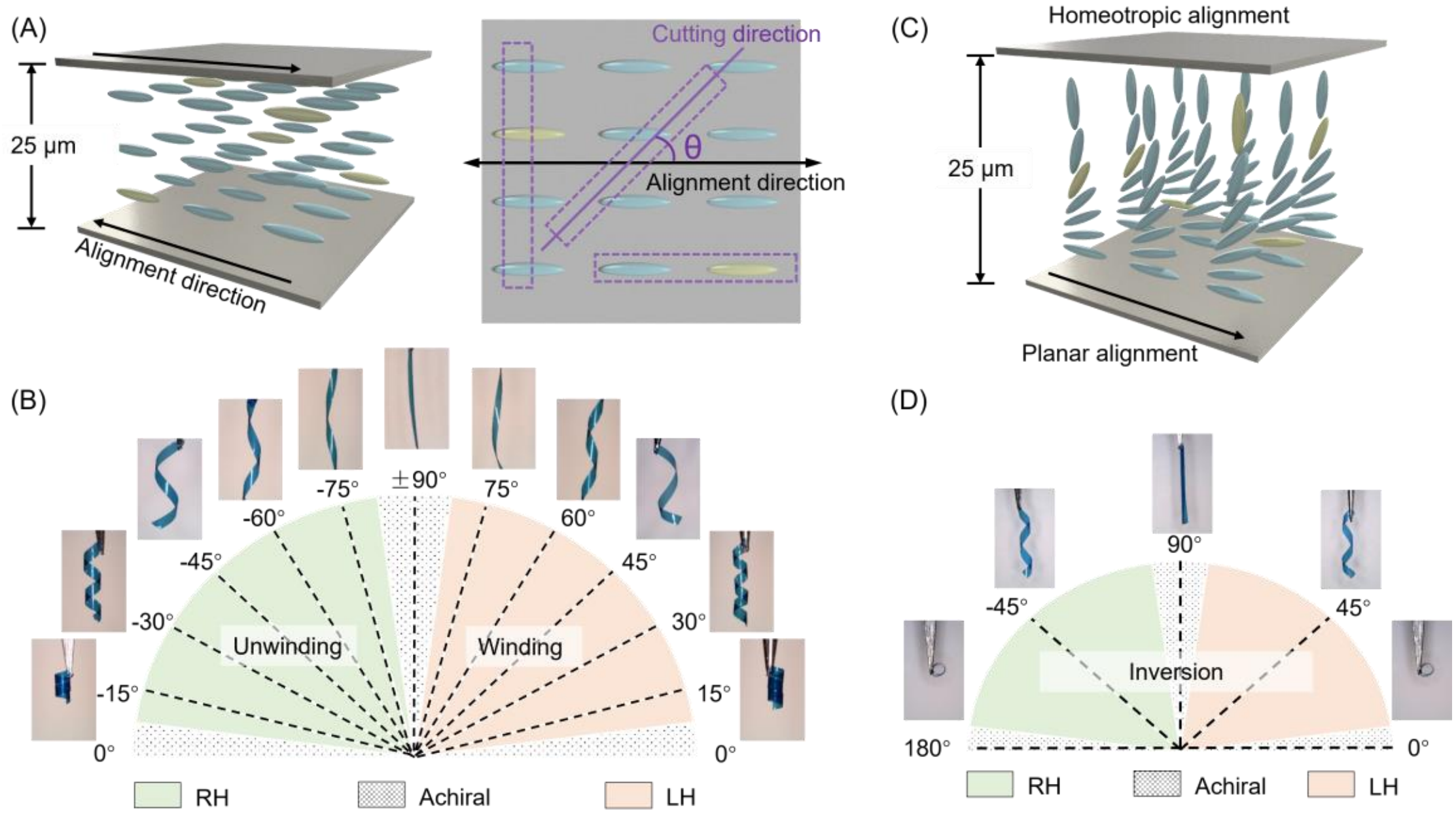

Figure S22 (A) Scheme of the **LCPN 2** with planar unidirectional alignment (left panel). The polymer ribbons were cut out at a specific angle (θ) with respect to the molecular alignment (right panel). (B) The diagram summarizing the shapes and actuation modes of the **LCPN 2** ribbons depending on the cutting angle. To generate shape, each ribbon was exposed to 365 nm UV light. LH and RH correspond to left- and right-handed helical shapes, respectively. The white dotted area denotes to achiral shapes which generally show bending or folding motion. (C) **LCPN 1** film with splayed molecular alignment where molecules have unidirectional planar alignment on one side of the film and vertical (homeotropic) orientation on the opposite side. (D) The diagram summarizing the shapes and actuation modes of the **LCPN 1** ribbons depending on the cutting angle. Here the symmetry is broken by inhomogeneous molecular alignment throughout the film thickness which fully determines the handedness of the helical shapes. The helical shapes are all inversed under the action of red light. LH and RH correspond to left- and right-handed helical shapes, respectively. The white dotted area denotes to achiral shapes.

13 Fatigue test

After 10 s of UV light irradiation, the **LCPN 2** film exhibited bending towards the light source. Upon subsequent exposure to blue light (455 nm) for 30 min, the film recovered to its initial upright state (Figure S23A), the principle of which is shown in Figure S20. Based on this irradiation protocol, the film underwent six consecutive light exposure cycles. The suspended LCPN film demonstrated stable and reversible deformation, with no noticeable permanent deformation or mechanical fatigue observed throughout multiple bending-recovery cycles (Figure S23B). These results highlight its excellent durability and highly repeatable actuation performance.

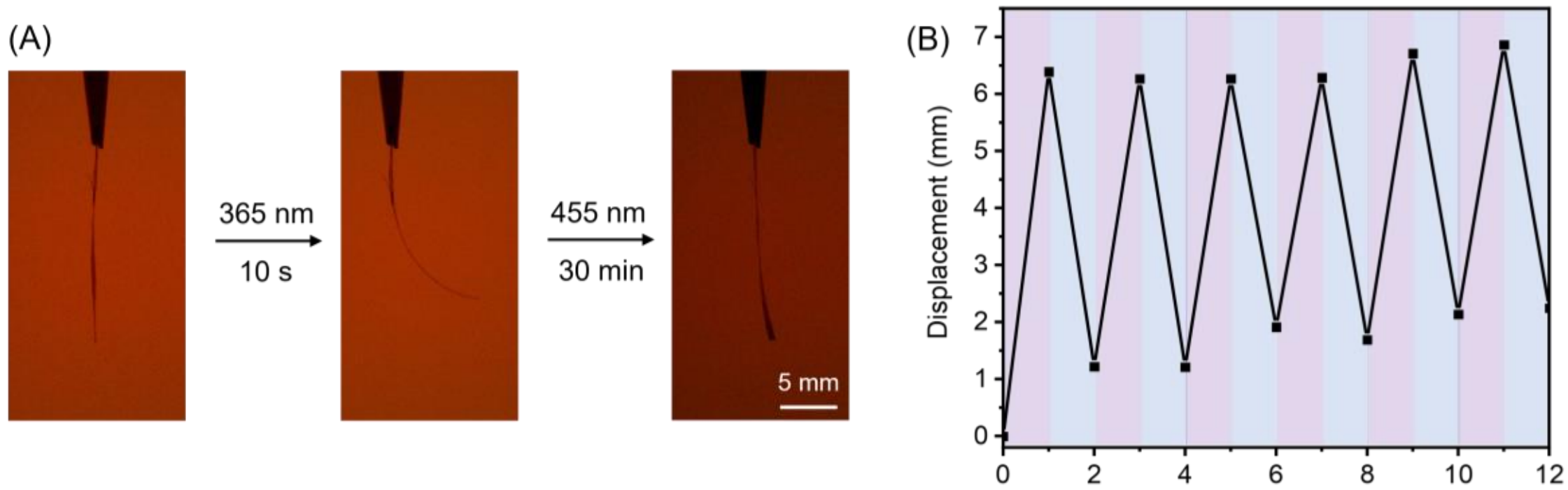

Figure S23 Fatigue test. (A) The actuation and deformation process of the film under UV and blue light irradiation. (B) The displacement of the film tip from its initial position after six UV-blue light cycles. The power densities of the UV and blue light were 1.25 mW/cm², 394.7 mW/cm², respectively.

14 Shape stability

We investigated the shape stability of **LCPN 2** strips (2 mm × 10 mm) cut along LC alignment axis. The film, after being exposed to UV light, was placed at 70 ℃ overnight, and according to the study of the THI process of **MDA** in LCPN (Figure S18 A), the motor **MDA** was transformed from the Z_M state to the Z_S state. The negligible shape change of the ribbon was observed likely due to minor geometrical changes between Z_M and Z_S states and possible internal relaxation of the network caused by heating (Figure S24A). Thermal treatment of the ribbon even with higher temperature (120 ℃) did not cause the shape shifting and ribbon remained unchanged (Figure S24B), confirming that the motor-driven LCPNs possess durable shape retention.

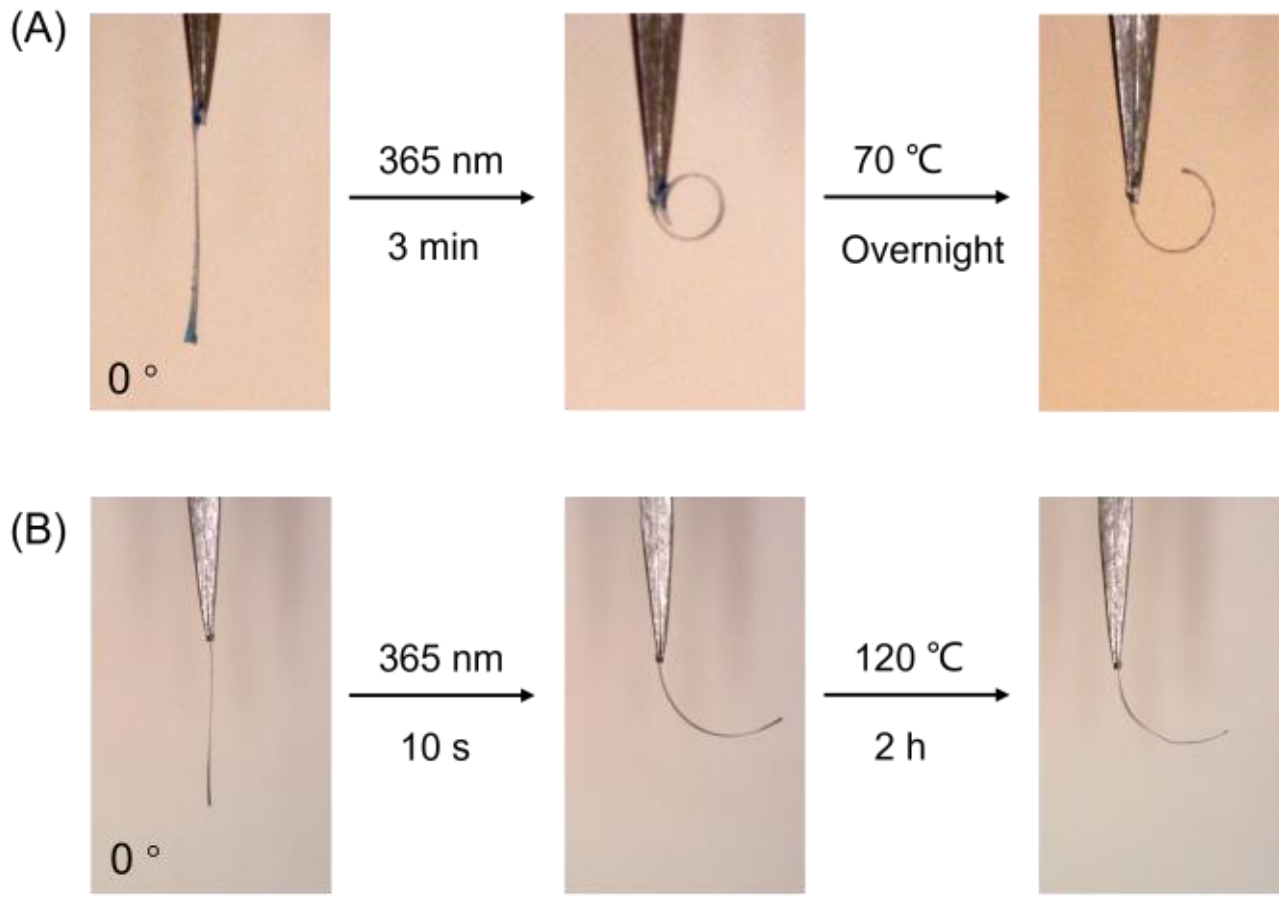

Figure S24 Shape change of the **LCPN 2** film under UV irradiation followed by annealing. The intensity of UV light was 1.2 mW/cm^2.

15 Control experiments

As a control we prepared **LCPN 2** films where **MDA** was replaced by **MMA**. Since **MMA** bearing only one acrylic moiety, it can only be attached to the network as a side group rather than a cross-linker (the **MMA** structure is shown in Figure S25A). We studied these films under the same illumination conditions and observed their photochemical and photothermal responses. As shown in Figure S25B, no shape change was observed after 3 min of UV irradiation using **MMA**. The polymer ribbon cut at angle of 45° in aspect to the alignment direction was exposed to UV light for 3 min. The shape change

was not observed, while the ribbon responded to red light by a rapid and reversible bending toward the light source (Figure S25C). In this experiment, the intensity of UV light used was very low (1.2 mW/cm²), which did not cause a temperature increase in the system. However, when red light was applied, infrared camera measurements confirmed a temperature rise of 30 ℃ in the film (since the film's material composition is the same as **LCPN 2**, it is expected to exhibit a consistent photothermal response under red light irradiation), as shown in Figure S31D. Thus, the covalent embedment of the motor as a cross-linker is necessary to harness motor rotation into macroscopic actuation.

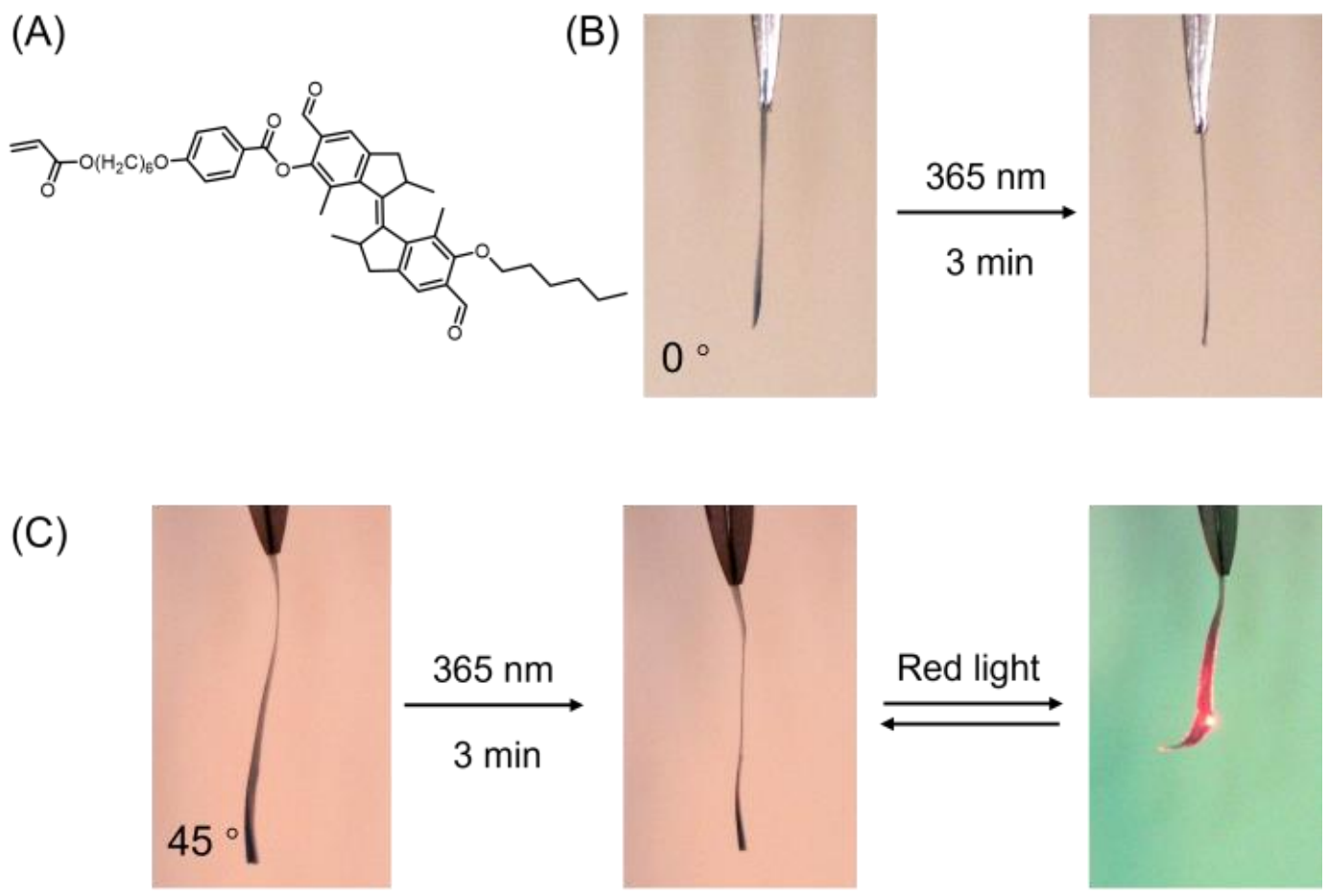

Figure S25 (A) The structure of **MMA**. (B) Photochemical effects in **MMA-LCPN 2** films at 0°. (C) Photochemical and photothermal effects of **MMA-LCPN 2** with a shear angle of 45°.

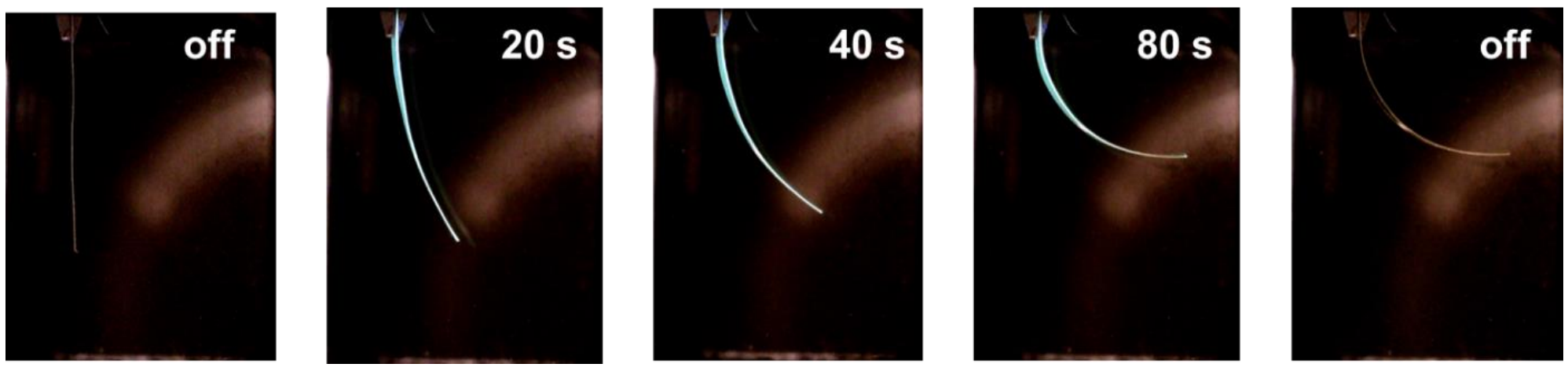

Figure S26. Evolution of the curvature of **LCPN 2** film upon irradiation with 365 nm UV light in water at 5 °C. The intensity of the UV light was 1.2 mW/cm². This experiment additionally proves that the shape shifting the solely originate from photochemical effect (rotation of molecular motors).

16 Light and heat shape encoding process in LCPN 3

For the rigid system **LCPN 3**, regardless of the method used, heat softened the **LCPN 3** system, allowing the motors to isomerize without being constrained by the network. Figure S27A shows that the film, laid flat on a glass slide and pre-heated, achieved significant bending deformation after being exposed to UV light for 9 min and then subjected to further heat treatment. Figure S27B demonstrates the effect of another treatment method: after the film was subjected to both light and heat and then cooled on a glass slide for 10 min, it remained straight when removed from the substrate due to “freezing” stresses in a glassy state of the network. However, subsequent annealing allowed stresses to be revealed yielding similar bending deformation.

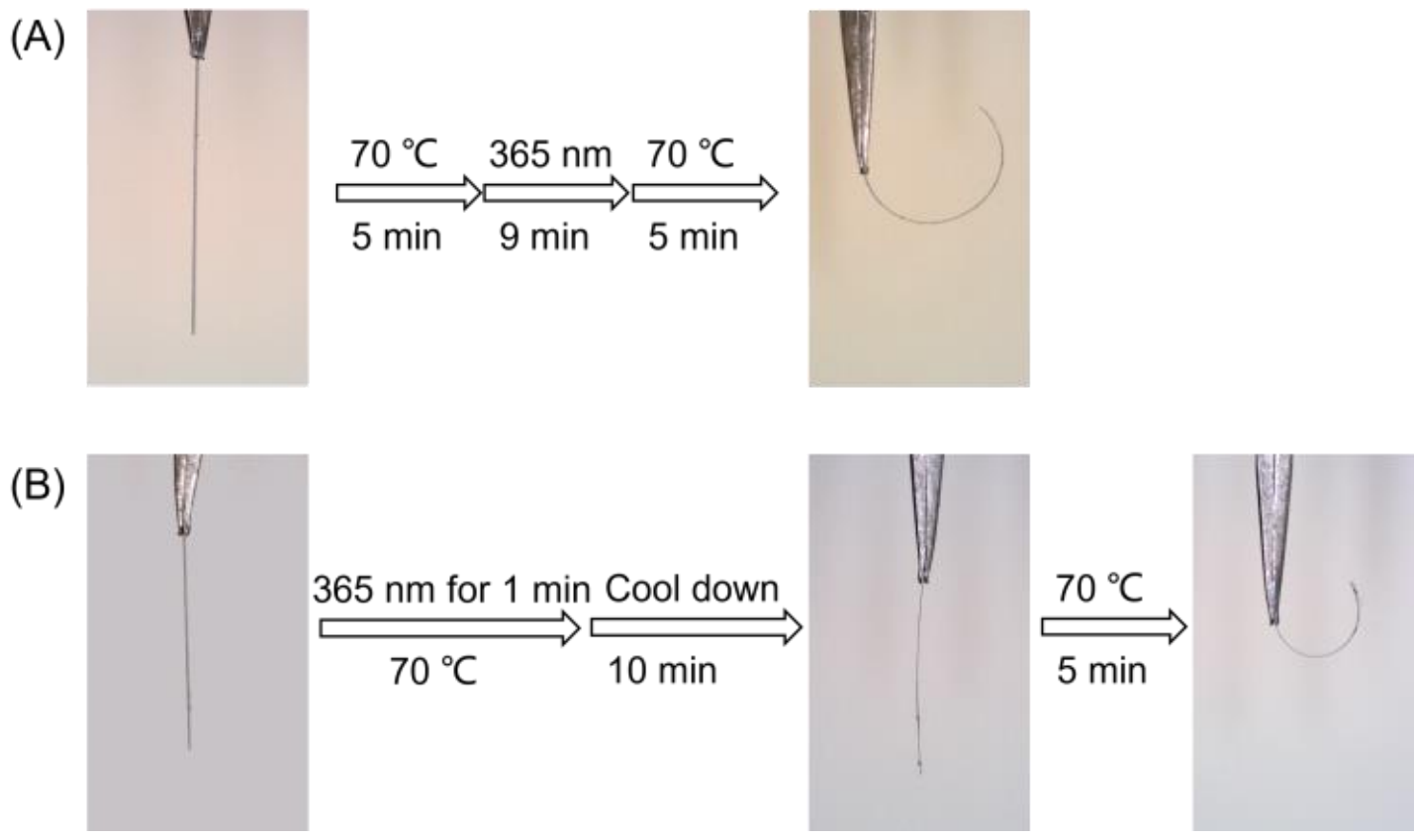

Figure S27 Different thermal treatment methods and their effects on the response of **LCPN 3**.

17 UV-vis spectra of LCPN 3 under UV irradiation

Upon UV irradiation of the rigid **LCPN 3** system, the molecular motors within the film undergo isomerization at a slow rate. Once the system reaches the photostationary state, heating **LCPN 3** to 70 ℃ leads to a rapid increase in the photoisomerized motor content (Figure S28). This phenomenon indicates that thermal activation softens the liquid crystalline network, thereby reducing the constraints imposed on the motors and enabling a greater fraction of them to undergo light-driven isomerization.

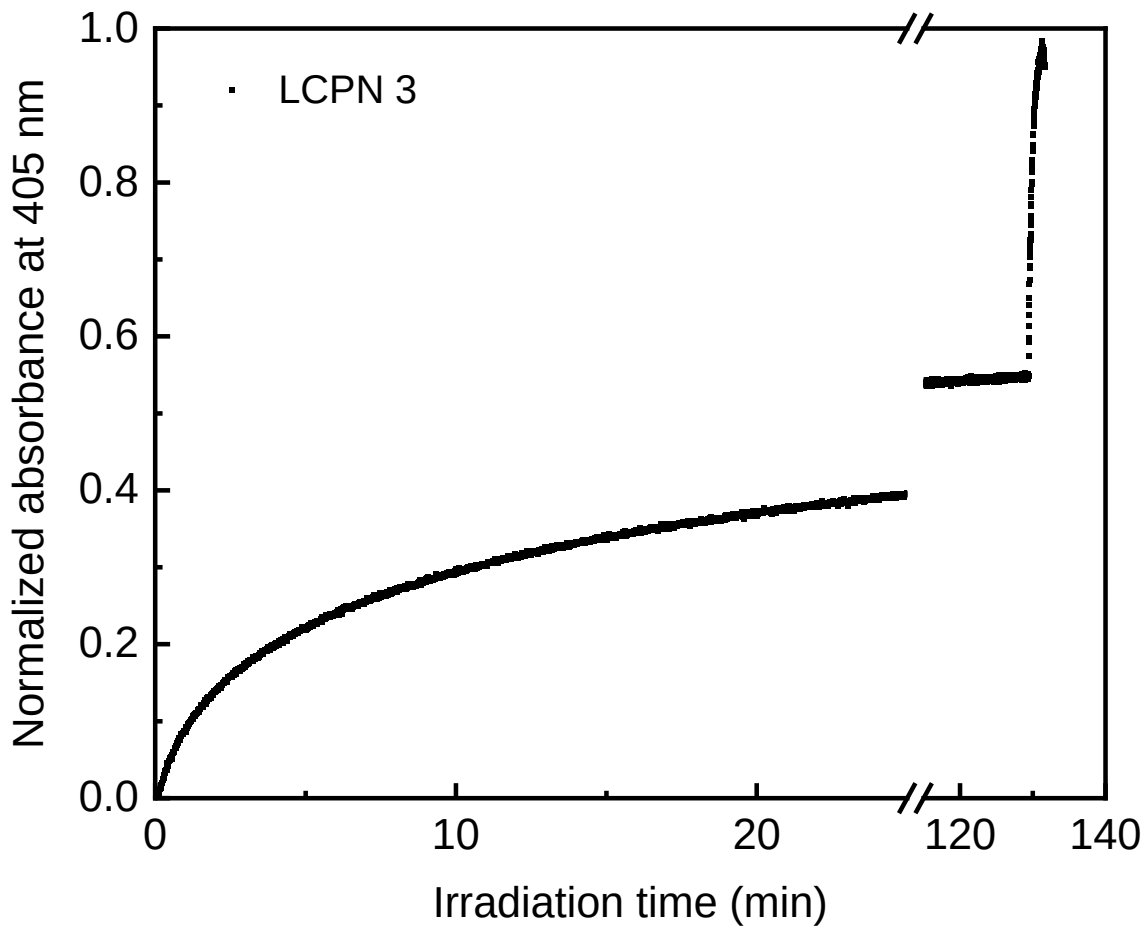

Figure S28 Variation of Z_M-**MDA** formation in **LCPN 3** corresponding to absorbance at 405 nm with UV light (365 nm) irradiation time. The sample was heated to 70 °C after an irradiation time of 130 min.

18 Mechanical properties of LCPNs

Two kinds of mechanical measurements were performed on the motorized LCPNs: i) elastic modulus detervination and ii) measurement of mechanical stress (force) generated by the rotating molecular motors. To measure light generated stress or force the flat LCPN ribbon was clamped between a load cell and a motorized stage, and a pre-strain of about 0.5% was applied. While maintaining a constant strain, the load cell was continuously recording mechanical stress induced by the ribbon exposed to light.

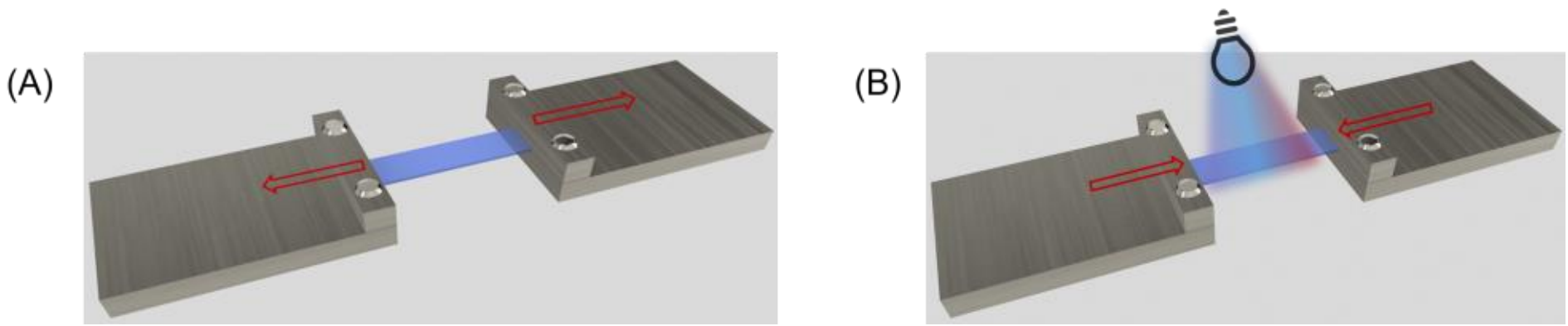

Figure S29 Schematic diagrams of the strain-stress measurements (A) and the measurement of photo-generated stress (B). Strain = constant.

18.1 Elastic moduli of LCPNs

The film is laid flat and clamped at both ends and stretched with a constant speed (50 µm/s) until the film breaks (Figure S29A). Elastic moduli were extracted from the recorded strain-stress curves and gathered in Table S2. All polymer ribbons of 25 µm were cut parallel to LC alignment so that the mechanical stretching was along this direction. Each set of data was tested at least three times, and the average values were then taken to ensure data reliability.

	LCPN 1	LCPN 2	LPCN 3	Azo-LCPN 1	Azo-LCPN 2	Control sample
Modulus (MPa)	52.3±3.4	93±8.6	1022.6±106.1	69	229.9±17.2	115±3.6

Table S2 Elastic moduli of LCPNs containing molecular motors and azobenzene switch as well as control sample (**LCPN 2** with **MMA**).

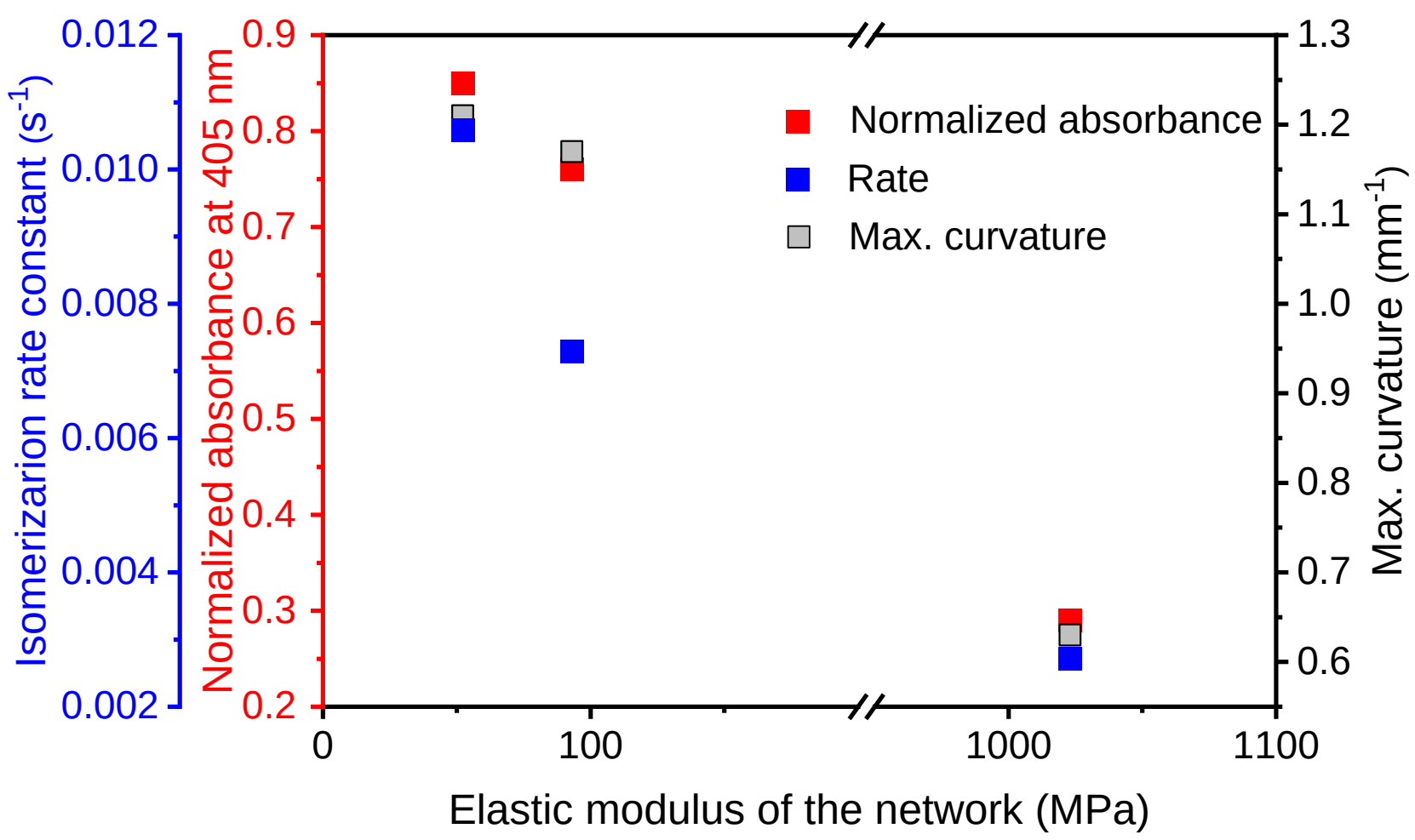

Figure S30 Normalized absorbance plateau (here represents photoconversion of Es state of MDA), isomerization rate constant and maximal curvature of **LCPN 1-3** plotted versus their elastic modulus. Normalized absorbance and rate constants were extracted from the kinetic data provided in Figure 3C, while values of the curvatures were taken from Figure S21. The trends indicated in the figure supports the hypnotises that an increasing network rigidity slows down and hinders the rotation of embedded molecular motors.

18.2 Photo-generated force

The film under investigation was clamped from both side and small strain (~0.5%) was applied. Pre-strained film was first equilibrated until constant force values and then irradiated to light with different wavelengths while monitoring mechanical force. The distance between the light source and the film was 15 cm. The schematic diagram is shown in Figure S29B.

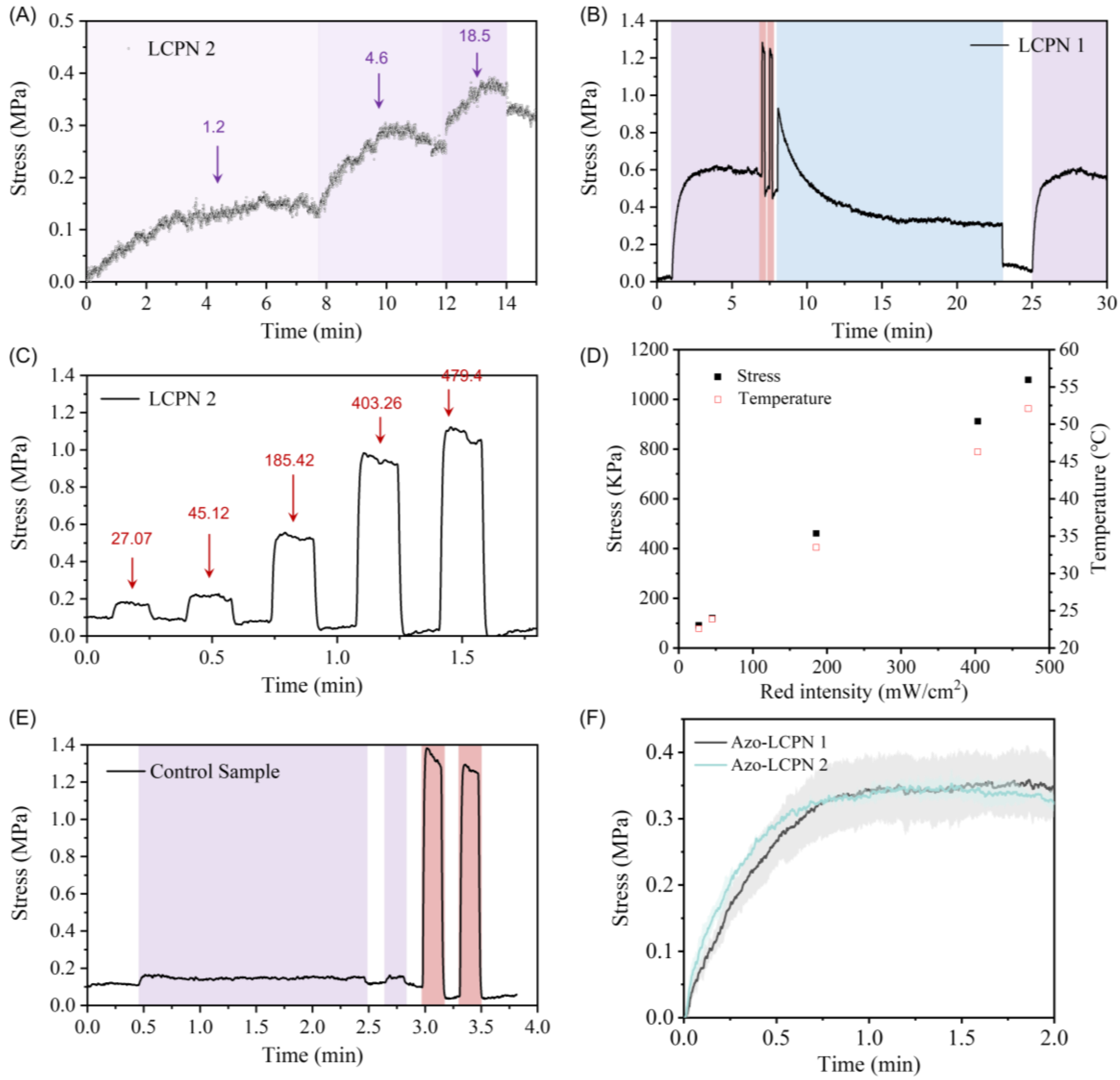

Figure S31 (A) The light-generated mechanical stress of **LCPN 2** as a function of the increasing UV light intensity. The purple-shaded area represents the period of UV exposure, with the specific UV intensities indicated by the numbers. (B) The stress values of **LCPN 1** under different exposure conditions. The purple, red, and blue regions represent the response effects of samples exposed to UV, red, and blue light, respectively. (C) Stress generated by **LCPN 2** upon red light illumination. The numbers are the red light intensity. The higher light intensity results in greater mechanical stress. Upon removal of the red light, the initial stress values restore due to the non-sustaining nature of photo-thermal effects. (D) Trend of the stress values of **LCPN 2** and its surface temperature vs. the red light intensity. (E) Variation of photo-generated mechanical stress upon irradiation of control samples (**LCPN 2** containing **MMA**). A small increase in the stress under UV irradiation is caused by photo thermal effect occurring at the used intensity (18.5 mW/cm^2) of UV light in analogues to the response observed under red light irradiation. (F) Variation of UV light generated stress of Azo-**LCPN 1** and Azo-**LCPN 2** with irradiation time. The gray and blue shaded areas correspond to the standard error. Data indicates that the stiffness of the network has negligible influence on the performance of azobenzene switch. In panels C and E the initial stress values (before irradiation) represent offset values originated from the pre-strain of the ribbons. The unit of light intensity in A and C is mW/cm^2.

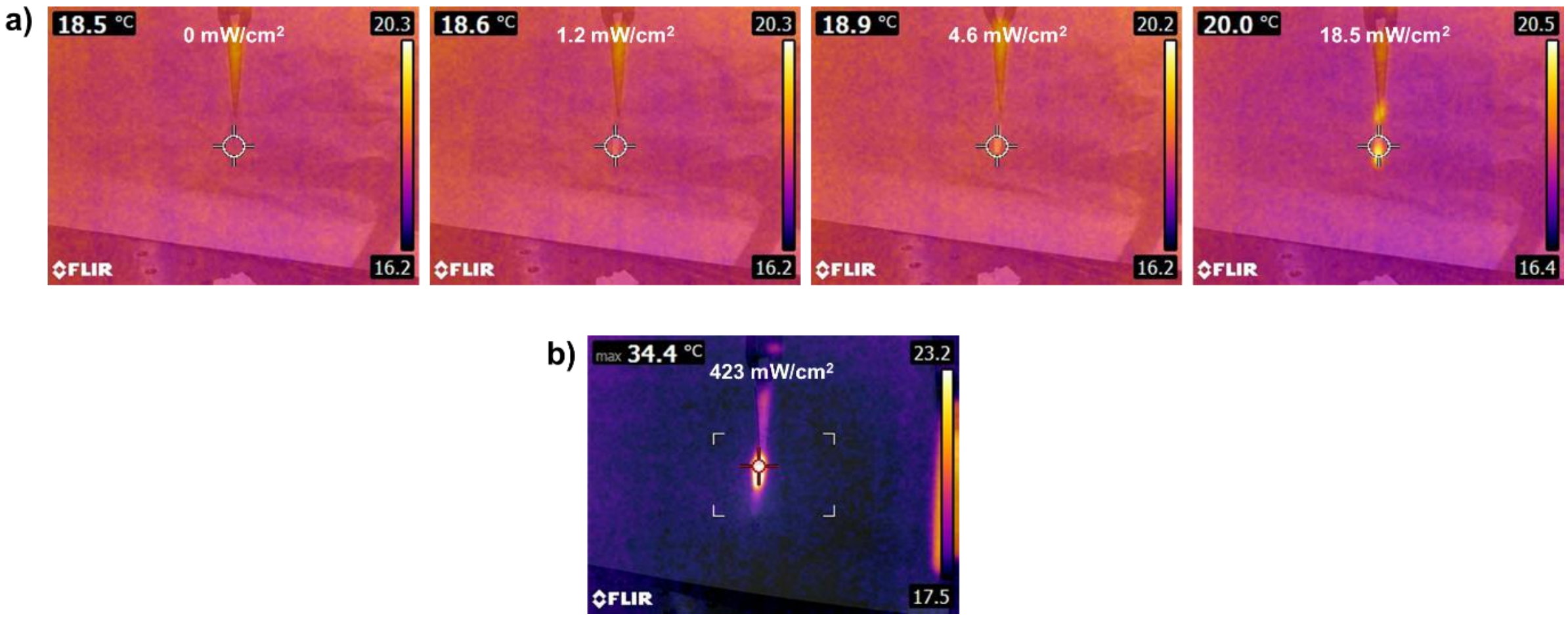

Figure S32 (A) Thermal images of the **LCPN 2** under UV (365 nm) illumination with increasing power. (B) Thermal image of the same film upon irradiation with 455 nm blue light.

UV-photogenerated stress versus time curves for **LCPN 1-3**, Azo-**LCPN 1** and control samples.

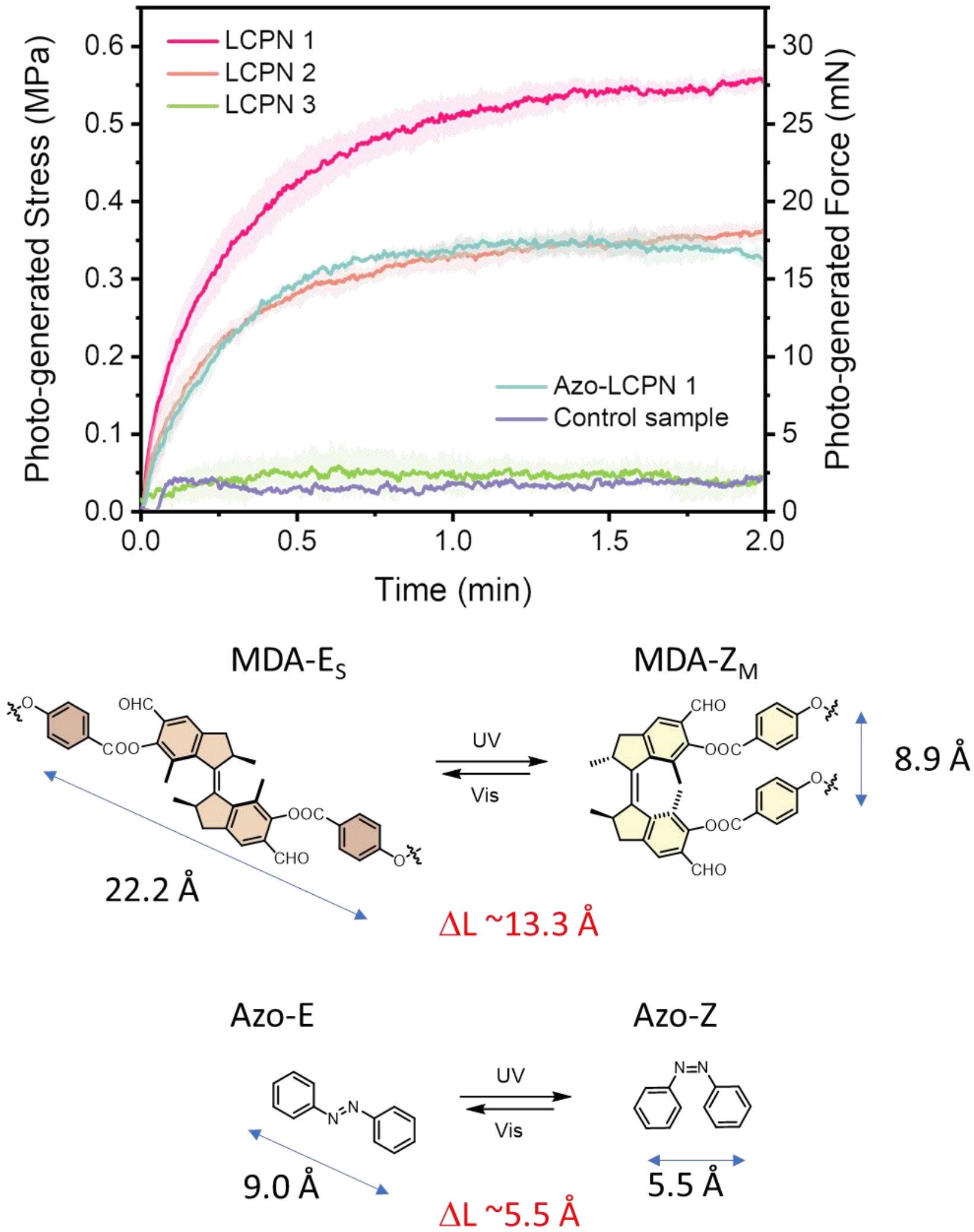

Figure S33 Building up stress/force exerted by the molecular motor rotation in **LCPN 1-3** under UV irradiation. If the motor is embedded in the network not as a covalent crosslinker it fails to exert any mechanical forces (control

sample). An azobenzene-based analog of **LCPN 1** generates comparatively lower mechanical stress. The intensity of UV light was 18.5 mW/cm^2. Bottom panel: change in the molecular length of motor and azobenzene upon reversible switching which accounts for photogenerated mechanical stresses when integrated into LCPNs.

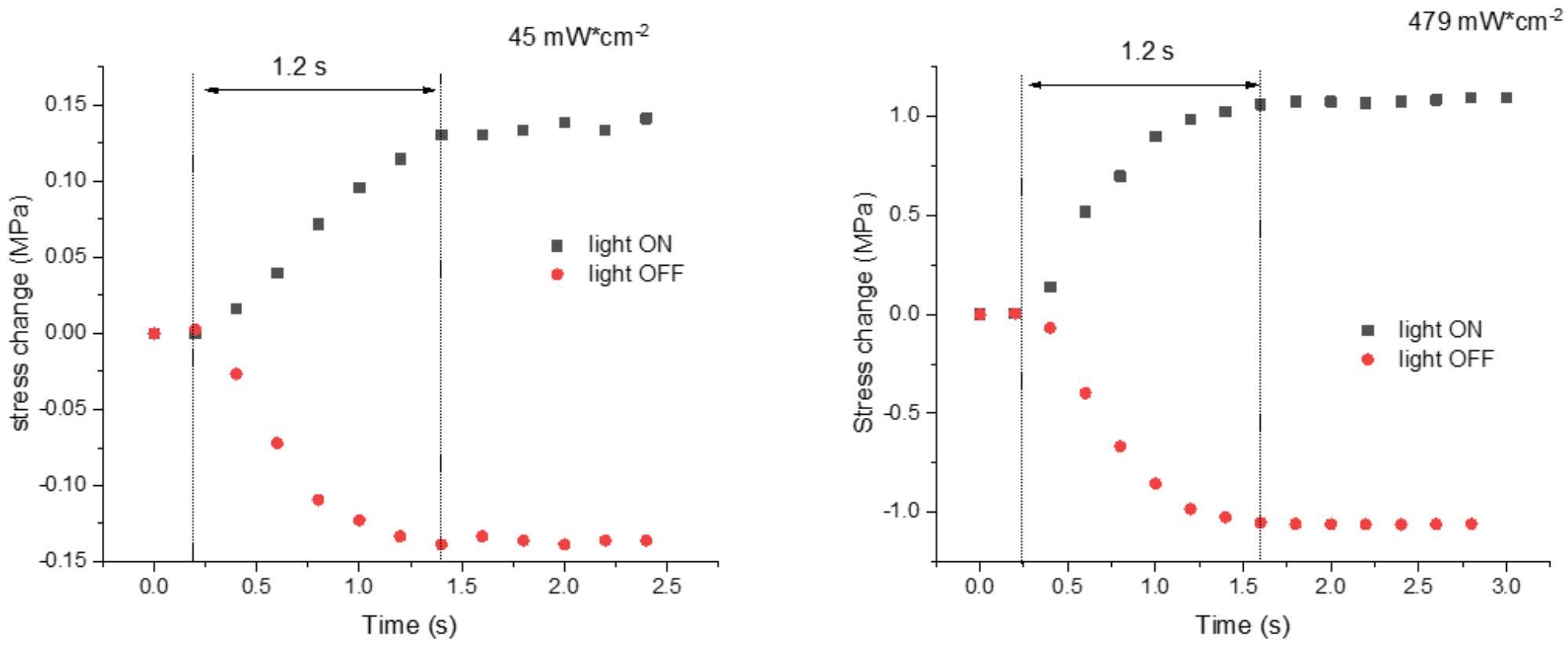

Figure S34. Evolution of the mechanical stress change in LCPN2 upon switching on and off red light of different intensities. The data shows that the stress builds up for both intensities withing the first second and remains stable.

We set UV light intensity at 18.5 mW/cm^2 with the purpose to activate as many motors as possible, while simultaneously generating a low thermal contribution (temperature increase doesn't exceed 1.5^{o}C), which enables the moment of switching on/off the light to be clearly discerned directly on the stress curves. Therefore the apparent photo-generated stress is composed of photo-thermal contribution and photo-chemical contribution. Photo-thermal contribution was estimated by the stress drop upon switching off the light and it was further subtracted from the apparent stress value in order to estimate true stress generated by photochemical effect (Table S3). According to Table S3, it was possible to rank the tested systems (**LCPN 1** > **LCPN 2** > Azo-**LCPN 1** > Azo-**LCPN 2** > **LCPN 3** > Control sample) which indicates that the photo-chemical efficiency and actuating ability of the **MDA** motor are superior to equivalent masses of azobenzene.

	Photo-chemical contribution (KPa)	Photo-thermal contribution (KPa)	Total photo-generated stress (KPa)
LCPN 1	524	36	560
LCPN 2	318	48	366
LCPN 3	2	20	22
Azo-LCPN 1	301	44	345
Azo-LCPN 2	286	29	315
Control Sample	0	40	40

Table S3 Mechanical stress generated by LCPNs. Total measured stress upon UV light (365 nm, 18.5 mW/cm^2) exposure is composed from photochemical and photothermal contributions which can be clearly distinguished on the stress curves.

19 Optimized geometries of four motor states

E_S			
C	-1.06403	-0.46069	0.16032
C	-1.03585	0.27193	-1.03307
C	-2.42555	0.73051	-1.29716
C	-3.30896	-0.30249	-0.61145
C	-2.47949	-0.60069	0.65957
C	-2.79154	1.96686	-1.66413
C	-1.86511	3.15849	-1.86224
C	-2.48637	4.17829	-0.87766
C	-3.95532	3.84474	-0.94416
C	-4.141	2.56211	-1.47552
C	-5.03471	4.61535	-0.57563
C	-6.32785	4.11134	-0.7439
C	-6.48728	2.86122	-1.33991
C	-5.41824	2.06994	-1.74735
C	0.09601	-0.96628	0.70074
C	1.30839	-0.77048	0.0336
C	1.30304	-0.08524	-1.18245
C	0.14578	0.42728	-1.76005
C	-3.46812	-1.58648	-1.43311
C	-1.91375	3.72169	-3.28635
C	0.19879	1.02404	-3.13804
C	-5.67858	0.79766	-2.50277
C	2.51298	-1.34842	0.66325
C	-7.44632	4.95872	-0.28298
H	-4.28267	0.10985	-0.35503
H	-2.68247	-1.5897	1.07371
H	-2.68775	0.14325	1.43455
H	-0.84085	2.91861	-1.58386
H	-2.2772	5.21324	-1.1534
H	-2.10617	4.01074	0.1347
H	-4.9006	5.60629	-0.1537
H	0.08463	-1.52682	1.63018
H	-2.49457	-2.05471	-1.60107
H	-3.92054	-1.39891	-2.40615
H	-4.0963	-2.29964	-0.89436
H	-1.25019	4.58548	-3.37026
H	-2.92703	4.0505	-3.53161
H	-1.61194	2.98553	-4.03042
H	0.82588	0.41478	-3.79025
H	0.61525	2.03318	-3.13114
H	-0.80064	1.0655	-3.5648

H	-6.45426	0.95919	-3.25228
H	-6.02188	-0.00397	-1.84518
H	-4.7727	0.46713	-3.00536
O	3.63643	-1.35922	0.20615
H	2.31117	-1.80922	1.64775
O	-8.61599	4.64245	-0.22548
H	-7.127	5.9658	0.04301
O	-7.76811	2.39668	-1.61681
C	-8.40722	1.76189	-0.60435
O	-7.87063	1.53252	0.45357
O	2.5031	0.14722	-1.84233
C	3.04338	-0.88932	-2.53012
O	2.43446	-1.91227	-2.73387
C	4.41545	-0.60516	-2.99616
C	5.10813	0.53778	-2.61006
C	5.05104	-1.54529	-3.81325
C	6.4174	0.74948	-3.0182
C	6.34967	-1.34677	-4.22879
C	7.04543	-0.19869	-3.8279
H	4.6297	1.26975	-1.97275
H	4.5151	-2.43713	-4.11389
H	6.93428	1.64263	-2.69746
H	6.85285	-2.06985	-4.85947
C	-9.79228	1.38541	-0.95119
C	-10.34855	1.64297	-2.19992
C	-10.56908	0.75345	0.02529
C	-11.65669	1.27691	-2.48623
C	-11.87002	0.39018	-0.24277
C	-12.42296	0.6466	-1.50422
H	-9.75963	2.13476	-2.96321
H	-10.13865	0.55336	0.99863
H	-12.0641	1.48698	-3.46492
H	-12.48181	-0.09707	0.5069
O	-13.70125	0.24938	-1.67502
C	-14.31496	0.43873	-2.95729
H	-13.72521	-0.08158	-3.71803
H	-14.32934	1.50616	-3.19706
C	-15.71706	-0.12264	-2.87391
H	-16.30031	0.40539	-2.1171
H	-15.69286	-1.18458	-2.62213
H	-16.21522	-0.00695	-3.8384
O	8.3168	-0.09584	-4.26915
C	9.09347	1.03765	-3.85682
H	9.13118	1.06827	-2.76391

H	8.61293	1.95295	-4.21482
C	10.47811	0.88042	-4.44452
H	10.43483	0.84418	-5.53474
H	10.95067	-0.0335	-4.07954
H	11.09671	1.7309	-4.15159
$\boldsymbol{E}_{\mathrm{M}}$			
C	3.10045	0.10772	4.77773
C	2.81537	0.42537	3.44281
C	3.98007	1.11327	2.84789
C	5.17403	0.76011	3.74305
C	4.48762	0.56325	5.12107
C	3.94662	2.02736	1.85868
C	2.77691	2.94528	1.48263
C	3.51168	4.26634	1.13234
C	4.8561	3.81717	0.64256
C	5.09991	2.50389	1.06755
C	5.76762	4.49681	-0.12938
C	6.94261	3.84529	-0.51703
C	7.12493	2.51272	-0.15719
C	6.21128	1.79114	0.60802
C	2.18399	-0.54776	5.56429
C	0.96056	-0.92651	5.00259
C	0.73243	-0.67861	3.65181
C	1.65013	-0.03542	2.82369
C	6.28575	1.7972	3.86481
C	1.72163	3.20443	2.55338
C	1.38803	0.07058	1.34794
C	6.41604	0.32018	0.83644
C	-0.05533	-1.60643	5.83452
C	7.95358	4.56256	-1.3236
H	5.5925	-0.20679	3.4437
H	5.02361	-0.14284	5.75657
H	4.45065	1.52367	5.6464
H	2.30033	2.58242	0.56564
H	2.96825	4.86444	0.39987
H	3.62248	4.86928	2.04002
H	5.59798	5.51833	-0.44837
H	2.38548	-0.78189	6.60275
H	6.92251	1.53596	4.71395
H	5.85872	2.78531	4.05514
H	6.92343	1.86911	2.98801
H	1.1107	4.05922	2.25224
H	1.05099	2.36636	2.72182
H	2.2012	3.45187	3.50402

H	0.69574	0.88219	1.11316
H	2.31725	0.25068	0.81083
H	0.94931	-0.85228	0.96888
H	6.78318	-0.16121	-0.06972
H	7.14446	0.12727	1.62724
H	5.47738	-0.15358	1.11677
O	7.81517	5.7075	-1.70456
O	0.11238	-1.8762	7.00674
H	8.87292	4.0084	-1.5687
H	-1.00613	-1.86179	5.34161
O	8.3045	1.87523	-0.54394
C	8.30434	1.22717	-1.73775
O	7.31895	1.15789	-2.43249
O	-0.49959	-1.04104	3.10556
C	-0.61174	-2.27868	2.55875
O	0.31447	-3.05234	2.51683
C	9.62419	0.64776	-2.05672
C	9.73861	-0.18325	-3.17618
C	10.75584	0.92062	-1.29383
C	10.95573	-0.72948	-3.51892
C	11.98961	0.38514	-1.63331
C	12.09285	-0.44597	-2.75051
H	8.85989	-0.39702	-3.77223
H	10.68209	1.56516	-0.42772
H	11.05572	-1.37835	-4.3807
H	12.85297	0.6199	-1.02733
C	-1.97122	-2.53813	2.04495
C	-2.19063	-3.69333	1.28725
C	-3.03799	-1.68345	2.3058
C	-3.44656	-3.98121	0.80059
C	-4.31059	-1.96775	1.83246
C	-4.5187	-3.12129	1.07412
H	-1.36229	-4.36016	1.08152
H	-2.88245	-0.78715	2.892
H	-3.62718	-4.86975	0.20762
H	-5.12255	-1.29114	2.05744
O	13.24154	-1.01846	-3.16535
C	14.44543	-0.73829	-2.43643
H	14.32238	-1.06397	-1.39927
H	14.6258	0.34074	-2.44429
C	15.57342	-1.4844	-3.11279
H	15.68501	-1.16015	-4.14916
H	15.38974	-2.56039	-3.09767
H	16.50842	-1.28515	-2.58558

O	-5.71249	-3.48844	0.56552
C	-6.85379	-2.66108	0.8343
H	-6.67543	-1.66001	0.43049
H	-6.99198	-2.58065	1.91651
C	-8.05132	-3.31087	0.17847
H	-8.21712	-4.31125	0.58275
H	-7.90816	-3.38624	-0.90114
H	-8.94213	-2.70911	0.36869
$\boldsymbol{Z}_S$			
C	-0.43555	-0.48229	5.64634
C	0.1054	-0.18807	4.38385
C	-0.29801	1.18222	4.0199
C	-1.56882	1.45517	4.813
C	-1.29475	0.66544	6.11784
C	0.34257	2.09653	3.28116
C	-0.29526	3.38337	2.77708
C	0.25104	3.44501	1.32881
C	1.60704	2.79455	1.45387
C	1.67195	2.04249	2.63856
C	2.69528	2.89231	0.62077
C	3.89336	2.27221	0.99589
C	3.9626	1.62704	2.22642
C	2.87623	1.50669	3.09254
C	-0.18027	-1.68686	6.25688
C	0.62353	-2.62511	5.59607
C	1.07687	-2.33888	4.31352
C	0.78922	-1.14956	3.64469
C	-2.81439	0.9295	4.09657
C	0.14428	4.60187	3.59142
C	1.15541	-1.00237	2.19504
C	3.07504	0.9058	4.45426
C	0.95219	-3.91212	6.24261
C	5.0718	2.33114	0.10545
H	-1.6848	2.52004	5.01467
H	-2.20957	0.3287	6.60859
H	-0.74111	1.28742	6.8279
H	-1.38265	3.31158	2.78472
H	-0.39014	2.86543	0.65747
H	0.31167	4.46364	0.9419
H	2.65579	3.44874	-0.30825
H	-0.58196	-1.92952	7.23361
H	-2.72507	-0.14309	3.90306
H	-2.95717	1.43613	3.13956
H	-3.70447	1.09183	4.70908

H	1.23328	4.7008	3.58351
H	-0.18127	4.51427	4.63028
H	-0.28321	5.51568	3.17201
H	0.88309	-1.90781	1.64923
H	2.22496	-0.84419	2.05648
H	0.61962	-0.1679	1.74854
H	4.00725	1.27283	4.88684
H	3.13805	-0.18321	4.41147
H	2.25413	1.17092	5.11675
O	0.52221	-4.24695	7.3281
O	5.05786	2.85763	-0.98923
H	1.6313	-4.57846	5.68874
H	5.99278	1.85899	0.48033
O	1.92638	-3.22819	3.65842
C	1.38003	-4.20399	2.88821
O	0.20603	-4.48216	2.9377
O	5.20285	1.15231	2.65439
C	5.41872	-0.18593	2.57812
O	4.6135	-0.94117	2.0885
C	2.38465	-4.83445	2.00951
C	3.68061	-4.33659	1.90651
C	2.00499	-5.92317	1.21716
C	4.59229	-4.89952	1.02599
C	2.90136	-6.49281	0.33977
C	4.20063	-5.97773	0.23054
H	3.98345	-3.48612	2.5019
H	0.99696	-6.31236	1.29266
H	5.58908	-4.48639	0.96189
H	2.61877	-7.3334	-0.2824
C	6.72543	-0.57896	3.13986
C	7.60852	0.34535	3.70853
C	7.08576	-1.9221	3.09041
C	8.82152	-0.0729	4.21208
C	8.30178	-2.35657	3.59369
C	9.17793	-1.42661	4.1582
H	7.34236	1.39319	3.75589
H	6.40304	-2.63738	2.64932
H	9.51493	0.6322	4.65431
H	8.55532	-3.40561	3.5407
O	5.00015	-6.58147	-0.67247
C	6.33061	-6.07351	-0.85197
H	6.88003	-6.16585	0.08973
H	6.27822	-5.0145	-1.12186
C	6.982	-6.8829	-1.95063

H	6.42837	-6.77935	-2.88574
H	7.02331	-7.93992	-1.6809
H	8.00129	-6.52626	-2.11102
O	10.38419	-1.73832	4.67416
C	10.79962	-3.11167	4.66111
H	10.83079	-3.46811	3.62735
H	10.07169	-3.71173	5.21506
C	12.16744	-3.18064	5.30295
H	12.13034	-2.82293	6.33362
H	12.88645	-2.57688	4.74597
H	12.51492	-4.21556	5.30849
$\boldsymbol{Z}_{\mathrm{M}}$			
C	1.22796	0.12357	5.40574
C	0.82334	0.43826	4.10525
C	1.45484	1.70391	3.69274
C	2.63098	1.93832	4.64301
C	2.20399	1.14719	5.90974
C	1.01006	2.55195	2.74506
C	1.86498	3.51365	1.91714
C	1.0869	3.5859	0.57457
C	-0.32067	3.20493	0.93112
C	-0.36012	2.63104	2.20581
C	-1.47073	3.35874	0.18861
C	-2.69453	2.95449	0.7299
C	-2.72247	2.49355	2.04548
C	-1.5846	2.35678	2.83249
C	0.73909	-0.99383	6.04529
C	-0.1559	-1.83337	5.3752
C	-0.46287	-1.55316	4.04239
C	0.05071	-0.45886	3.3549
C	2.89218	3.39794	5.00666
C	3.29724	3.04952	1.66176
C	-0.19165	-0.35445	1.87465
C	-1.74797	2.01181	4.28676
C	-0.71081	-2.96787	6.13934
C	-3.88782	3.05775	-0.13431
H	3.5404	1.47911	4.24082
H	3.05149	0.69868	6.42991
H	1.70881	1.82583	6.61218
H	1.86265	4.51098	2.36996
H	1.50638	2.85863	-0.12851
H	1.14993	4.56862	0.10512
H	-1.44334	3.77785	-0.81217
H	1.02548	-1.22871	7.06549

H	1.97022	3.85843	5.37094
H	3.63409	3.44799	5.80772
H	3.26098	3.99643	4.17682
H	3.28763	2.03883	1.24543
H	3.77177	3.71069	0.93209
H	3.92065	3.04002	2.55285
H	0.53927	0.30624	1.41315
H	-0.10176	-1.3415	1.41876
H	-1.18972	0.02263	1.64841
H	-2.60392	2.55011	4.69503
H	-1.92829	0.94565	4.43675
H	-0.85965	2.28521	4.85159
O	-1.51019	-3.7893	5.74124
O	-4.99155	2.61408	0.10433
H	-3.69477	3.58354	-1.08757
H	-0.33176	-3.02125	7.1764
O	-1.39142	-2.32627	3.36306
C	-1.05047	-3.5702	2.95518
O	0.06778	-4.0112	3.06849
O	-3.95087	2.23258	2.64316
C	-4.40516	0.95962	2.57396
O	-3.77734	0.07458	2.04149
C	-2.20144	-4.27527	2.35318
C	-3.41767	-3.63709	2.12783
C	-2.06341	-5.62364	2.01205
C	-4.49154	-4.32359	1.57952
C	-3.12362	-6.31896	1.47203
C	-4.34839	-5.67401	1.25686
H	-3.53334	-2.58962	2.37363
H	-1.11739	-6.12246	2.184
H	-5.42395	-3.80269	1.41305
H	-3.03206	-7.36687	1.21275
C	-5.71652	0.79564	3.23325
C	-6.4728	1.88718	3.67179
C	-6.21049	-0.4914	3.41936
C	-7.69484	1.68779	4.27778
C	-7.43402	-0.70715	4.03502
C	-8.18498	0.38918	4.46413
H	-6.10336	2.89426	3.52937
H	-5.62514	-1.33879	3.0843
H	-8.29341	2.52529	4.61542
H	-7.78826	-1.71873	4.17328
O	-5.33139	-6.43758	0.73488
C	-6.6224	-5.84754	0.53081

H	-6.53165	-5.01483	-0.17297
H	-6.99521	-5.4593	1.48339
C	-7.53198	-6.92685	-0.01246
H	-7.61161	-7.75539	0.69395
H	-7.1535	-7.30927	-0.96233
H	-8.5297	-6.5157	-0.17759
O	-9.38993	0.2956	5.06462
C	-9.95461	-1.00812	5.26322
H	-9.28391	-1.5981	5.89505
H	-10.05183	-1.50862	4.29527
C	-11.30467	-0.82517	5.91977
H	-11.96557	-0.23278	5.2841
H	-11.20185	-0.32487	6.88456
H	-11.76543	-1.80124	6.08361

Table S4. Optimized geometric structure data for xyz coordinates of four motor states.

20 Supplementary videos 1-9:

Video S1

Winding behavior of UV-exposed **LCPN 2** films (θ = 45 °) stimulated by red light.

Video S2

Unwinding behavior of UV-exposed **LCPN 2** films (θ = -45 °) stimulated by red light.

Video S3

LCPN 1 films (θ = 135 °) undergo RH→LH helical inversion upon red light stimulation.

Video S4

LCPN 1 films (θ = 135 °, exposed to UV light on the planar-aligned side) undergo a winding behavior under red light.

Video S5

LCPN 1 films (θ = 135 °, exposed to UV light on the homeotropic-aligned side) undergo a unwinding behavior under red light.

Video S6

Jumping motion.

Video S7

Rotary motion (side view).

Video S8

Rotary motion (top view).

Video S9

Climbing motion.

21 References

[1] Sheng, J., Danowski, W., Sardjan, A. S., et al. Formylation boosts the performance of light-driven overcrowded alkene-derived rotary molecular motors. *Nat. Chem.* **16**, 1330-1338 (2024).

[2] Stranius, K., Börjesson, K. Determining the photoisomerization quantum yield of photoswitchable molecules in solution and in the solid state. *Sci. Rep.* **7**, 41145 (2017). https://doi.org/10.1038/srep41145

[3] Kuhn, H. J., Braslavsky, S. E., Schmidt, R. Chemical actinometry (IUPAC Technical Report). *Pure Appl. Chem.* **76**, 2105-2146 (2004). http://dx.doi.org/10.1351/pac200476122105

[4] Montalti, M., Credi, A., Prodi, L., Gandolfi, M. T. Handbook of Photochemistry. (CRC Press, 2006).

[5] Najibi, A., Goerigk, L. DFT-D4 counterparts of leading meta-generalized-gradient approximation and hybrid density functionals for energetics and geometries. *J. Comput. Chem.*, **41**, 2562 (2020). https://doi.org/10.1002/jcc.26411

[6] Barone, V., Cossi, M. Quantum calculation of molecular energies and energy gradients in solution by a conductor solvent model. *J. Phys. Chem. A* **102**, 1995-2001 (1998). https://doi.org/10.1021/jp9716997

[7] Neese, F., Wennmohs, F., Becker, U., Riplinger, C. The ORCA quantum chemistry program package. *J. Chem. Phys.* **152**, 224108 (2020). https://doi.org/10.1063/5.0004608
